# Sustained Improvement of Arterial Stiffness and Blood Pressure after Long-Term Rosuvastatin Treatment in Patients with Inflammatory Joint Diseases: Results from the RORA-AS Study

**DOI:** 10.1371/journal.pone.0153440

**Published:** 2016-04-19

**Authors:** Eirik Ikdahl, Silvia Rollefstad, Jonny Hisdal, Inge C. Olsen, Terje R. Pedersen, Tore K. Kvien, Anne Grete Semb

**Affiliations:** 1 Preventive Cardio-Rheuma Clinic, Department of Rheumatology, Diakonhjemmet Hospital, Oslo, Norway; 2 Section of Vascular Investigations, Oslo University Hospital Aker, Oslo, Norway; 3 Department of Rheumatology, Diakonhjemmet Hospital, Oslo, Norway; 4 Centre of Preventive Medicine, Oslo University Hospital, Ullevål, Oslo, Norway; 5 Faculty of Medicine, University of Oslo, Oslo, Norway; University of Bologna, ITALY

## Abstract

**Objective:**

Patients with inflammatory joint diseases (IJD) have a high prevalence of hypertension and increased arterial stiffness. The aim of the present study was to evaluate the effect of long-term rosuvastatin treatment on arterial stiffness, measured by augmentation index (AIx) and aortic pulse wave velocity (aPWV), and blood pressure (BP) in IJD patients with established atherosclerosis.

**Methods:**

Eighty-nine statin naïve IJD patients with carotid atherosclerotic plaque(s) (rheumatoid arthritis n = 55, ankylosing spondylitis n = 23, psoriatic arthritis n = 11) received rosuvastatin for 18 months to achieve low-density lipoprotein cholesterol goal ≤1.8 mmol/L. Change in AIx (ΔAIx), aPWV (ΔaPWV), systolic BP (ΔsBP) and diastolic BP (ΔdBP) from baseline to study end was assessed by paired samples t-tests. Linear regression was applied to evaluate associations between cardiovascular disease (CVD) risk factors, rheumatic disease specific variables and medication, and ΔAIx, ΔaPWV, ΔsBP and ΔdBP.

**Results:**

AIx, aPWV, sBP and dBP were significantly reduced from baseline to study end. The mean (95%CI) changes were: ΔAIx: -0.34 (-0.03, -0.65)% (p = 0.03), ΔaPWV: -1.69 (-0.21, -3.17)m/s^2^ (p = 0.03), ΔsBP: -5.27 (-1.61, -8.93)mmHg (p = 0.004) and ΔdBP -2.93 (-0.86, -5.00)mmHg (p = 0.01). In linear regression models, ∆aPWV was significantly correlated with ΔsBP and ΔdBP (for all: p<0.001).

**Conclusions:**

There is an unmet need of studies evaluating CVD prevention in IJD patients. We have shown for the first time that long-term intensive lipid lowering with rosuvastatin improved arterial stiffness and induced a clinically significant BP reduction in patients with IJD. These improvements were linearly correlated and may represent novel insight into the pleiotropic effects by statins.

**Trial Registration:**

ClinicalTrials.gov NCT01389388

## Introduction

Patients with inflammatory joint diseases (IJD), including rheumatoid arthritis (RA), ankylosing spondylitis (AS) and psoriatic arthritis (PsA), have an increased risk of atherosclerotic cardiovascular disease (CVD) [1].

Hypertension (HT) is common in patients with IJD and is one of the most important predictors of CVD in the general population [2,3]. Considering the increased CVD risk in IJD patients, and that HT confers the same relative risk for CVD events in IJD patients as in the general population [4], the number of deaths attributable to HT may be relatively higher in this patient group compared to non-IJD subjects [5]. Furthermore, higher blood pressure (BP) levels are correlated with the presence of subclinical atherosclerosis and increased arterial stiffness [6, 7].

Arterial stiffness reflects the cumulative effect of traditional and novel CVD risk factors on the large arteries [8], and is increased in IJD patients compared to the general population [9–11]. The augmentation index (AIx) and the aortic pulse wave velocity (aPWV) are measures of arterial stiffness that have been shown to independently predict CVD in the general population [12,13].

The effect of statins on lipid levels in patients with IJD is well known [14,15]. We have recently reported in the the **RO**suvastatin in **R**heumatoid **A**rthritis, **A**nkylosing **S**pondylitis and other inflammatory joint diseases, the **RORA-AS** study, that rosuvastatin treatment over 18 months induced carotid plaque regression in IJD patients [16]. Short-term statin therapy has previously been reported to reduce arterial stiffness in IJD patients [17,18]. In addition, statin treatment has been shown to improve arterial stiffness in non-IJD individuals with established atherosclerotic disease [19]. However, to our knowledge, the effect of long-term intensive statin therapy on arterial stiffness in IJD patients with established atherosclerotic disease has not yet been evaluated. Furthermore, statins have been shown to have antihypertensive properties in other high CVD risk patients [20,21], but the antihypertensive effect of statins has not been ascertained in patients with IJD.

In this study we aimed firstly to evaluate if long-term (18 months) rosuvastatin therapy would reduce arterial stiffness, systolic (sBP) and diastolic (dBP) BP in IJD patients with established atherosclerosis, and if reduction in these parameters were correlated. Secondly, we explored if baseline levels of rheumatic disease specific variables and CVD risk factors were predictors of improvement in arterial stiffness and BP. Thirdly, we aimed to assess whether the changes in these variables during the study period were correlated with the changes in arterial stiffness and BP.

## Materials and Methods

The study design of the RORA-AS study has previously been described [16]. All patients signed a written informed consent form and the study was approved by the Norwegian South East Regional Health committee and registered with ClinicalTrials.gov Id: NCT01389388. The **E**uropean **U**nion **D**rug **R**egulating **A**uthorities **C**linical **T**rials (EudraCT) number is 2008-005551-20.

Rosuvastatin therapy was initially given in 20 mg dose once daily (o.d.), except for patients aged >70 years, who received an initial dose of 5 mg o.d. The dose was doubled every fortnight, until low-density lipoprotein cholesterol (LDL-c) goal (≤ 1.8 mmol/l) or maximal rosuvastatin dose (40 mg o.d) was reached. Patients remained on rosuvastatin therapy for 18 months. Traditional CVD risk factors, including lifestyle variables and the presence of established CVD were recorded. In addition, laboratory analyses and rheumatic disease activity measures were obtained. To identify carotid plaque(s) (CP) and measure carotid intima-media thickness (c-IMT), bilateral B-mode ultrasonography examinations were performed according to recommendations [22].

Brachial BP was measured using an Omron M7 after 5 minutes rest in a supine position. If the sBP was ≥140 mmHg or the dBP was ≥90 mmHg, three BP were recorded and the mean of the last two measurements were calculated. Analyses on sBP and dBP included only data from patients not taking antihypertensive medication or on stable antihypertensive therapy during the study.

The Sphygmocor apparatus (Atcor, West Ryde, Australia) is the most commonly used device for estimating arterial stiffness [23], and was used to obtain the arterial stiffness parameters AIx and aPWV. By applanation tonometry, the apparatus equalizes the arterial circumferential pressure to obtain accurate pressure waveforms. Several recordings were made in each patient and the recordings considered to have the highest quality according to predetermined requirements were selected for further analyses [AtCor Medical, technical notes, http://atcormedical.com/technicalnotes.html) (accessed June 2014)]. Patients suffering from atrial fibrillations were excluded from this analysis.

AIx was derived by applying a validated transfer system to recordings of the arterial pressure waves at the radial artery. By definition, AIx is the change in pressure between the second and first systolic peaks as a percentage of the pulse pressure, and was standardized to a heart rate of 75 beats per minute, as described by Pauca *et al*. [24]. To determine aPWV, pulse pressure waveforms were recorded at the carotid and femoral artery. Pulse wave transit time from the heart to these two recording sites was calculated by relating to the R wave in a simultaneously recorded electrocardiogram as the reference frame. The distances from the carotid and femoral recording sites to the sternal notch was obtained by using a measuring tape. The Sphygmocor software calculated aPWV from the pulse wave transit time from the heart to either the carotid or the femoral artery and the distances from the carotid and femoral recording sites to the sternal notch. This is the standard methodology for obtaining aPWV and AIx and has been used in several other studies published from our research group [9,25].

### Statistics

Descriptive statistics are expressed as numbers (%) for dichotomized variables, and mean±standard deviation (SD) and median and interquartile range (IQR) for normally and non-normally distributed continuous variables, respectively. Diagnosis groups were compared using Analysis of Variance (ANOVA), and Chi-square tests as appropriate. Non-normally distributed variables were logarithmically transformed before conducting these analyses. Number of CP across the various IJD diagnoses was compared using the non-parametric Kruskal-Wallis test.

The changes in AIx (ΔAIx), aPWV (ΔaPWV), sBP (ΔsBP) and dBP (ΔdBP) from baseline to study end were analyzed using paired-samples t-tests. Analyses were performed for RA, AS and PsA patients, separately and for all patients as a combined IJD group.

Both unadjusted and adjusted linear regression analyses were applied to evaluate for significant correlations between the changes in arterial stiffness (ΔAIx and ΔaPWV) and BP levels (ΔsBP and ΔdBP). Furthermore, we also explored if these dependent variables could be predicted by the baseline levels of AIx, aPWV, sBP or dBP. The adjusted linear regression analyses included age and gender as covariates, in addition to change in/ initiation of antihypertensive medication for analyses of arterial stiffness as dependent variables. We attempted to evaluate if the possible correlations between baseline BP and ∆BP variables could be results of regression towards the mean effects by performing additional mixed model analyses with random intercept, random slope and slope-baseline interaction covariates. We were able to perform these additional analyses since BP had been measured at 3 months in addition to being evaluated at baseline and at study end. However, since arterial stiffness was only measured at baseline and study end we were not able to construct mixed model analyses for ∆AIx and ∆aPWV.

Additional unadjusted linear regression analyses were performed to 1) evaluate if ΔAIx, ΔaPWV, ΔsBP and ΔdBP could be predicted by baseline demographic data (age, gender), use of biologic disease modifying anti-rheumatic drugs (bDMARDs), synthetic disease modifying anti-rheumatic drugs (sDMARDs), nonsteroidal anti-inflammatory drugs (NSAIDs), inflammation [C-reactive protein (CRP), erythrocyte sedimentation rate (ESR)], rheumatic disease activity [disease activity score in 28 joints (DAS28), ankylosing spondylitis disease activity score (ASDAS)] or LDL-c, and 2) evaluate correlations of ΔAIx, ΔaPWV, ΔsBP and ΔdBP with area under the curve (AUC) inflammation (ESR, CRP) and change in c-IMT, CP height, inflammation (CRP, ESR), rheumatic disease activity (DAS28, ASDAS), LDL-c, body mass index (BMI) and lifestyle parameters (physical activity, smoking habits, alcohol consumption), during the study period.

Model assumptions and validity were assessed using residuals. Two-sided p-values < 0.05 were considered significant. Statistical analyses were performed using SPSS version 21 (IBM SPSS Statistics for Windows, Armonk, NY).

## Results

**Fig 1:**
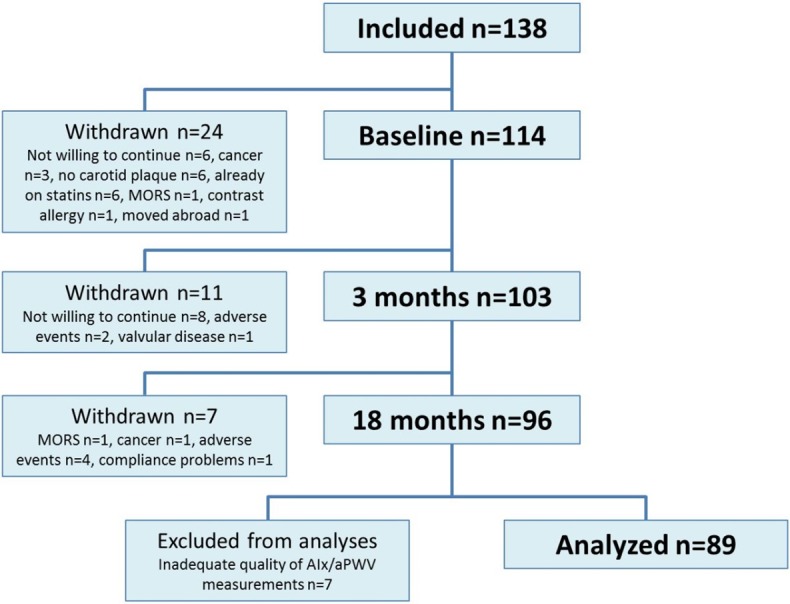
Flow chart of the ROsuvastatin in Rheumatoid Arthritis, Ankylosing Spondylitis and other inflammatory joint diseases (RORA-AS) study. Of the 138 patients who were initially included in the study, 24 patients withdrew. Ninety-six patients completed 18 months of treatment with rosuvastatin. Adequate quality of AIx/aPWV measurements was obtained in 89 patients who were included in the analyses.

A flow chart of the RORA-AS study is shown in Fig 1. Patients with AS, RA and PsA were well matched concerning baseline characteristics, except physical activity (p = 0.002) and for the expected gender differences (p = 0.01), use of prednisolone (p = 0.02) and synthetic disease modifying anti-rheumatic drugs (sDMARDs) (p = 0.01) (Table 1). Antihypertensive medication was either initiated or changed during the study period in 18 patients, and these patients were excluded from further statistical analyses related to sBP and dBP. The mean rosuvastatin dose given at 18 months was 30 mg o.d.

**Table 1:** Baseline patient characteristics.

	RA	AS	PsA	All	RA/AS/PsA p-value
Number, n (%)	55 (62)	23 (26)	11 (12)	89 (100)	-
Age, median (IQR)	62.0 (57.0–68.0)	59.0 (53.0–64.0)	60.0 (52.0–64.0)	61.0 (56.0–67.0)	0.07
Sex, female n (%)	41 (75)	15 (35)	6 (55)	55 (62)	0.004
Disease duration, median (IQR) (years)	16.0 (6.5–22.3)	22.0(16.0–30.0)	13.0(2.0–30.0)	16.0(8.0–26.0)	0.11
Physically active, n (%)	24 (44)	18 (78)	2 (18)	44 (49)	0.002
**CVD risk factors, mean (SD)**					
Smoke, n (%)	11 (20)	3 (13)	3 (27)	17 (19)	0.59
BMI (kg/m^2^)	25.0 (2.9)	25.2 (2.7)	26.1 (3.7)	25.2 (2.9)	0.55
TC (mmol/L)	6.43 (1.24)	6.19 (0.90)	6.62 (1.07)	6.39 (1.13)	0.54
HDL-c(mmol/L)	1.78 (0.48)	1.53 (0.45)	1.60 (0.51)	1.69 (0.49)	0.09
TG (mmol/L), median (IQR)	1.2 (0.9–1.6)	1.4 (0.9–1.9)	1.1 (0.7–2.9)	1.2 (0.9–1.8)	0.67
LDL-c (mmol/L)	3.95 (0.96)	3.97 (0.86)	4.32 (1.00)	4.00 (0.94)	0.48
sBP (mmHg)	142.6 (19.8)	144.8 (14.2)	147.4 (24.6)	143.7 (19.0)	0.71
dBP (mmHg)	82.7 (8.9)	85.2 (8.2)	87.9 (10.8)	84.0 (9.0)	0.16
**Co morbidities**					
Hypertension, n (%)	31 (56)	16 (70)	6 (55)	53 (60)	0.52
Diabetes, n (%)	3 (6)	2 (9)	0 (0)	5 (6)	0.59
CVD, n (%)	6 (11)	2 (9)	0 (0)	8 (9)	0.51
Number of CP, median (range)	1 (1–5)	1 (1–3)	2 (1–3)	1 (1–5)	0.34
**Inflammatory markers**					
ESR (mm/h), mean (SD)	15.8 (10.3)	12.9 (9.8)	13.9 (5.7)	14.8 (9.7)	0.47
CRP (mg/L), median (IQR)	3.0 (1–4)	1 (1–3)	3 (2–6)	2 (1–4)	0.22
**Medication, n (%)**					
Prednisolone	21 (38)	2 (9)	2 (18)	25 (28)	0.02
NSAIDs	21 (38)	12 (52)	5 (46)	38 (43)	0.52
sDMARDs	36 (66)	5 (22)	9 (82)	50 (56)	0.002
bDMARDs	15 (27)	8 (35)	5 (46)	28 (32)	0.53
Anti-HT medication	16 (29)	4 (17)	2 (18)	22 (25)	0.48

RA: rheumatoid arthritis, AS: ankylosing spondylitis, PsA: psoriatic arthritis, n: number, IQR: Inter-quartile range, SD: standard deviation, Physically active: Physically active ≥ 1 per week, BMI: body mass index, TC: total cholesterol, HDL-c: high-density lipoprotein cholesterol, TG: triglycerides, LDL-c: low-density lipoprotein cholesterol, sBP: systolic blood pressure, dBP: diastolic blood pressure, Hypertension: >140/90 mmHg, self-reported hypertension and/or on antihypertensive medication, BP: blood pressure, CVD: cardiovascular disease (including myocardial infarction, percutaneous coronary intervention, coronary artery bypass graft surgery, cerebral ischemic stroke, transient ischemic attack), CP: carotid plaque(s), ESR: erythrocyte sedimentation rate, CRP: c-reactive protein, NSAIDs: non-steroidal anti-inflammatory drugs, sDMARDS and bDMARDs: synthetic and biologic disease modifying anti-rheumatic drugs, Anti-HT medication: Anti-hypertensive medication (beta receptor antagonists, calcium channel antagonists, angiotensin converting enzyme inhibitors, angiotensin II receptor antagonists, diuretics), SD: standard deviation, IQR: interquartile range.

**Fig 2:**
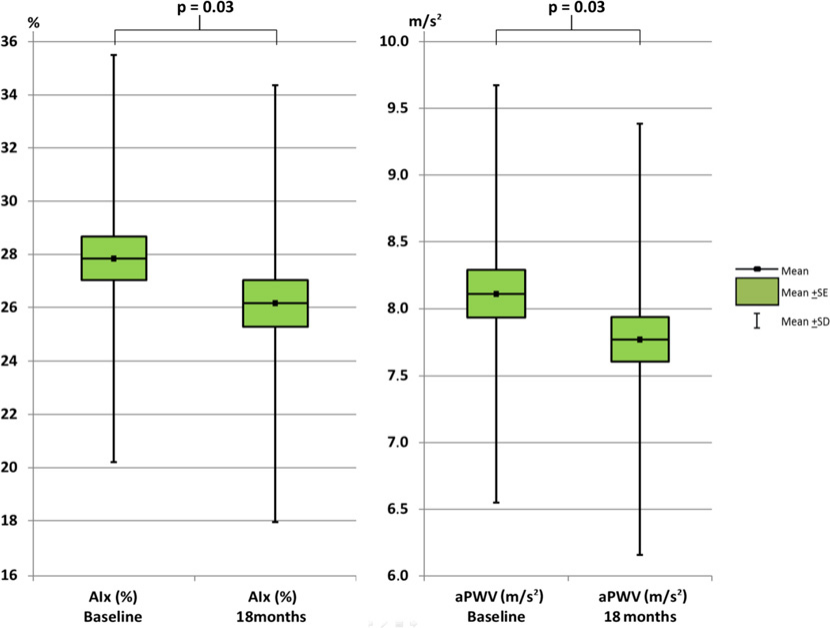
Change in aortic pulse wave velocity (aPWV) and augmentation index (AIx) after 18 months rosuvastatin therapy. SE: standard error of the mean, SD: standard deviation of the mean.

**Fig 3:**
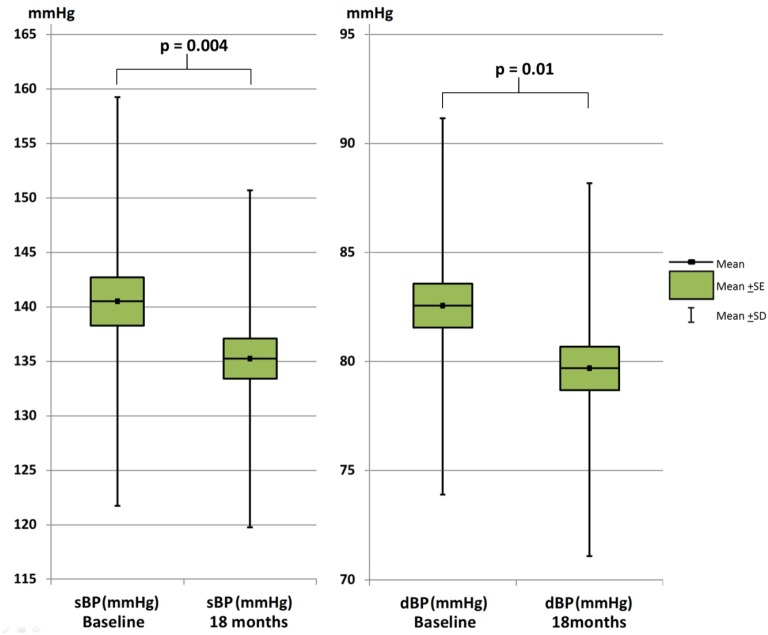
Change in systolic (sBP) and diastolic (dBP) blood pressure after 18 months rosuvastatin therapy. SE: standard error of the mean, SD: standard deviation of the mean.

Arterial stiffness improved significantly after 18 months of intensive lipid lowering with rosuvastatin (Fig 2). The mean (95% confidence interval [CI]) reduction in AIx and aPWV was -0.34 (-0.03, -0.65) % and -1.69 (95% CI: -0.21, -3.17) m/s^2^, respectively. Brachial BP was also significantly improved as the mean (SD) sBP was reduced by -5.27 (95% CI: -1.61, -8.93) mmHg and dBP was reduced by -2.93 (-0.86, -5.00) mmHg (Fig 3). The trends towards decreasing AIx, PWV, sBP and dBP were present in all diagnose groups (S1 Table).

The adjusted linear regression analyses with ∆AIx and ∆aPWV as dependent variables (Table 2) indicated that ∆AIx and ∆aPWV was predicted by the baseline AIx and aPWV levels, respectively, in that patients with higher arterial stiffness at baseline experienced a more substantial reduction during the study (p<0.001). However, since AIx and aPWV were only measured twice, it was not possible to evaluate if this association could be a consequence of the regression towards the mean phenomenon. Furthermore, the linear regression analyses displayed that ∆aPWV was longitudinally correlated with ∆sBP (p<0.001) and ∆dBP (p = 0.002), indicating that a more substantial reduction in aPWV was correlated with a larger BP lowering effect. For AIx, the linear regression analyses did not show significant correlations with sBP or dBP.

**Table 2:** Adjusted linear regression analyses with change in augmentation index (ΔAIx), pulse wave velocity (ΔaPWV), systolic (∆sBP) and diastolic (∆dBP) blood pressure as dependent variables.

	∆AIx (%)	∆aPWV (m/s^2^)	∆sBP (mmHg)	∆dBP (mmHg)
	β (95% CI)*p-value	β (95% CI)* p-value	β (95% CI)† p-value	β (95% CI)†p-value
**Baseline Aix** (%)	-0.53 (-0.74, -0.32) p<0.001	-0.34 (-1.47, 0.79) p = 0.55	-0.02 (-0.58, 0.54) p = 0.95	0.08 (-0.24, 0.39) p = 0.63
**Baseline aPWV** (m/s^2^)	-0.01 (-0.06, 0.04) p = 0.67	-0.44 (-0.66, -0.22) p<0.001	-5.22 (-8.04,-2.40) p<0.001	-2.73 (-4.32, 1.14) p = 0.001
**Baseline sBP** (mmHg)	-0.02 (-0.11, 0.07) p = 0.73	-0.01 (-0.03, 0.01) p = 0.25	-0.55 (-0.72, -0.39) p<0.001	-0.24 (-0.34, -0.14) p<0.001
**Baseline dBP** (mmHg)	-0.13 (-0.31, 0.04)p = 0.14	0.001 (-0.03, 0.04)p = 0.95	-0.71 (-1.09, -0.32) p<0.001	-0.56 (-0.75, -0.36) p<0.001
**∆AIx**(%)			0.01 (-0.50, 0.53) p = 0.96	-0.02 (-0.31, 0.27)p = 0.90
**∆aPWV**(m/s^2^)			8.41 (5.88, 10.94)p<0.001	4.04 (2.51, 5.58)p<0.001
**∆sBP**(mmHg)	0.004 (-0.09, 0.10) p = 0.93	0.04 (0.02, 0.06) p<0.001		
**∆dBP**(mmHg)	-0.02 (-0.19, 0.15) p = 0.84	0.05 (0.02, 0.08) p = 0.002		

* Adjusted for age, sex and change of/initiation of antihypertensive therapy during the study period.

† Adjusted for age and gender. Excluding patients for whom antihypertensive therapy was changed/initated.

Δ: change during study period, CI: confidence interval, aPWV: aortic pulse wave velocity, AIx: Augmentation index, sBP: systolic blood pressure, dBP: diastolic blood pressure.

For ∆sBP and ∆dBP, the adjusted multiple linear regression analyses (Table 2) showed an association with the baseline sBP, dBP and aPWV levels (p ≤ 0.001 for all), indicating that the antihypertensive effect by statins could be larger in patients with higher baseline levels of aPWV and sBP/dBP. The mixed model analyses indicated that this was not a result of the regression towards the mean phenomenon (p<0.001, data not shown). However, this statistical approach is not absolute and the potential influence of the latter phenomenon cannot be completely disregarded.Moreover, the adjusted regression analyses revealed that ∆sBP and ∆dBP were correlated with ∆aPWV (p < 0.001 for all), further underlining the close relationship between the reduction in arterial stiffness and BP levels.

Estimates from the unadjusted linear regression analyses with ∆AIx, ∆aPWV, ∆sBP and ∆dBP as dependent variables (S2 Table and S3 Table) did not differ considerably from the estimates from the adjusted analyses.

Additional unadjusted linear regression analyses revealed that age was the only significant baseline predictor for change in arterial stiffness or BP levels, as increasing age could predict ∆aPWV (p = 0.01) and ∆dBP (p = 0.03) (S2 Table). In unadjusted linear regression analyses evaluating ΔAIx, ΔaPWV, ΔsBP and ΔdBP as dependent variables in relation to AUC inflammation (ESR, CRP) and change in c-IMT, CP height, inflammation, rheumatic disease activity, LDL-c, BMI and lifestyle parameters did not reveal statistically significant correlations (S3 Table).

## Discussion

The main findings in the present study were that BP and arterial stiffness were significantly improved in IJD patients with established atherosclerosis who received intensive lipid lowering with rosuvastatin over 18 months. Further, we found a significant correlation between the observed reduction in aPWV and BP levels, irrespective of change in antihypertensive medication. Our results also showed that the reduction in BP seen in IJD patients treated with rosuvastatin was larger in patients with higher baseline BP.

Previous studies have reported a significant reduction in AIx and aPWV in RA patients following short-term (12 weeks), low- and moderate-dose primary CVD preventive treatment regimens with simvastatin and atorvastatin [17,18]. However, the beneficial changes in the structure of the vascular wall have been shown to be greater shortly after statin treatment initiation compared to long-term statin treatment [26]. We show for the first time that long-term statin treatment in IJD patients has a sustained beneficial effect on arterial stiffness.

Furthermore, our study is the first to indicate a potentially clinically significant antihypertensive effect by statins in patients with IJD.Two previous meta-analyses have reported a modest, but clinically important antihypertensive effect by statins in other high CVD risk populations [20,21]. Our analyses also indicated that baseline sBP and dBP levels could be significant predictors of the reductions in sBP and dBP in our patients, as the analyses showed that patients with higher baseline BP levels experienced a greater antihypertensive effect of rosuvastatin. Although one cannot definitively exclude the possibility that this was due to a regression towards the mean effect, our results are in line with previous studies in the general population where statins did not substantially reduce the BP in normotensive individuals [21,27].

We did not detect a correlation between BP lowering and inflammatory variables in our patients. Furthermore, we did not detect a correlation between the improvement in arterial stiffness or BP with LDL-c reduction in our study, which is in line with previous reports [18,19,21].

It has been hypothesized that the antihypertensive effects by statins is related to their ability to reduce arterial stiffness [28,29]. Our results are supportive of this hypothesis, in that we observed a strong correlation between the improvement in aPWV and the reduction of sBP, independent of alterations in antihypertensive therapy. The association between increasing BP and arterial stiffening has been well established [30]. In this regard, hypertension has traditionally been presented as the cause of, rather than the result of arterial stiffness. However, it has also been shown that arterial stiffness may antedate and contribute to the development of hypertension [31], thus creating a vicious cycle of increasing BP and progressive arterial stiffening. Further studies are warranted to elucidate if decreasing arterial stiffness represent a mechanism by which statins exert their BP lowering effect.

The change in AIx during the study was not correlated with or predicted by traditional CVD risk factors, rheumatic disease variables or medication use. Although AIx and aPWV are both indices of arterial stiffness, they cannot be regarded as interchangeable measures [32]. While aPWV is directly related to the aortic stiffness, AIx depends upon aPWV in addition to other variables, including ventricular function, vascular diameter and compliance of small muscular arteries and arterioles [33–35]. Compounding the various properties of AIx and aPWV, it has been argued that the two indices can be influenced differently by the same drug (e.g. statins) [32], and our results are in line with this suggestion.

The relatively low number of patients in the study was a consequence of that the statistical strength of the RORA-AS study was calculated on the basis of its primary end-point, namely CP height reduction. Nevertheless, the current study is to our knowledge the largest study to evaluate the effect of statins on arterial stiffness in patients with RA and the first to evaluate this matter in patients with AS or PsA. Results from a control group in a randomized designed trial would also have been advantageous. However, the ethical implications of giving a placebo to patients with established atherosclerosis over the course of 18 months precluded this. On the other hand, as arteries become stiffer with increasing age [36], it is not likely that the reduction in arterial stiffness was a result of natural aging progress. Thus, a placebo group would possibly have revealed an even larger difference in arterial stiffness. Furthermore, we did not detect any correlations between AIx, aPWV or BP and other factors known to influence physical properties of the arterial tree and BP levels (i.e. smoking, alcohol consumption, BMI and physical activity) [3,35]. Thus, it is not likely that our results are caused by the patients adapting a healthier lifestyle during the study period. Finally, it should be noted that the BP measurements were not obtained by ambulatory BP monitoring (ABPM), which could have provided more accurate BP measurements and reduces the risk of white coat hypertension. However, the BP measurements were standardized and potential environmental influences minimized (as described in the methods) to reduce the risk of bias to the measurements.

In conclusion, IJD patients with established atherosclerosis experienced a reduction of arterial stiffness and a clinically important BP reduction after intensive lipid lowering with rosuvastatin for 18 months. In addition, we have shown that the improvement of aPWV and reduction in BP levels were correlated; this may provide novel insight into the mechanisms responsible for the pleiotropic effects by statins.

## Supporting Information

S1 File The ROsuvastatin in Rheumatoid Arthritis, Ankylosing Spondylitis and other inflammatory joint diseases (RORA-AS) Study Protocol in English. (PDF)

S2 File The ROsuvastatin in Rheumatoid Arthritis, Ankylosing Spondylitis and other inflammatory joint diseases (RORA-AS) Study Protocol in original language (Norwegian). (PDF)

S3 File The Transparent Reporting of Evaluations with Nonrandomized Designs (TREND) checklist for the RORA-AS study. (PDF)

S1 Table Change in augmentation index (AIx), aortic pulse wave velocity (aPWV), systolic (sBP) and diastolic (dBP) blood pressure after 18 months rosuvastatin therapy. RA: Rheumatoid arthritis, AS: Ankylosing spondylitis, PsA: Psoriatic arthritis, Paired samples t-test by diagnose.
(DOCX)

S2 Table Evaluation for predictors of change in the dependent variables augmentation index (ΔAIx), aortic pulse wave velocity (ΔaPWV), systolic (ΔsBP) and diastolic (ΔdBP) blood pressure. Δ: Change from baseline to study end, aPWV: aortic pulse wave velocity, AIx: Augmentation index, sBP: Systolic blood pressure, dBP: Diastolic blood pressure, bMARDs: Biological disease-modifying anti-rheumatic drugs, sDMARDs: synthetic disease-modifying anti-rheumatic drugs, NSAIDs: Nonsteroidal anti-inflammatory drugs, CRP: C-reactive protein, ESR: Erytrocyte sedimentation rate, DAS28: Disease activity score 28 joints, ASDAS: Ankylosing spondylitis disease activity score, LDL-c: Low-density lipoprotein cholesterol, Unadjusted linear regression analyses.
(DOCX)

S3 Table Evaluation for variables correlated with change in the dependent variables augmentation index (ΔAIx), aortic pulse wave velocity (ΔaPWV), systolic (ΔsBP) and diastolic (ΔdBP) blood pressure. Δ: Change from baseline to study end, AIx: Augmentation index, aPWV: aortic pulse wave velocity, sBP: systolic blood pressure, dBP: diastolic BP, BMI: Body mass index, Anti-HT: Antihypertensive medication (beta receptor antagonist, calcium channel antagonists, angiotensin converting enzyme inhibitors, angiotensin II receptor antagonists, diuretics), AntiHT change: New antihypertensive medication, drug switch or dose adjustment, Physical activity: ≥ 1 per week, reported on patient questionnaire, CRP: C-reactive protein, AUC: Area under the curve, ESR: Erytrocyte sedimentation rate, DAS28: Disease activity score 28 joints, ASDAS: Ankylosing spondylitis disease activity score, LDL-c: Low-density lipoprotein cholesterol, c-IMT: Carotid intima-media thickness, CP: Carotid plaque. Unadjusted linear regression analyses.
(DOCX)
