# Supplementary material for: Sustained Improvement of Arterial Stiffness and Blood Pressure after Long-Term Rosuvastatin Treatment in Patients with Inflammatory Joint Diseases: Results from the RORA-AS Study
Source: PLoS One. 2016 Apr 19;11(4):e0153440. doi: 10.1371/journal.pone.0153440 (PMC4836743; doi:10.1371/journal.pone.0153440)
Supplement: S1 File — (PDF) [file pone.0153440.s001.pdf]

Cholesterol plaques in carotid and coronary arteries and the effect of Rosuvastatin in rheumatoid arthritis, ankylosing spondylitis and other inflammatory joint diseases

RORA AS-study

English Version 060711

Eudract nr 2008-005551-20
Sponsor's protocol Code Number 1957
REK 2009/2219
SLV 09/090799-11

AG Semb[1], E Stranden [2], S Provan[1], A Eirheim[1], I Holme[3], TR Pedersen[3], S Nicholls[4], NE Kløw[5], TK Kvien[1]

[1] Department of Rheumatology, Diakonhjemmet Hospital, Oslo, Norway
[2]Circulation Physiology Laboratory, Oslo Vascular Centre, Oslo University Hospital, Aker, Oslo, Norway
[3]Department of Preventive Medicine, Oslo University Hospital, Ullevål, Oslo, Norway
[4]Department of Cardiovascular Medicine, Cleveland Clinic, Cleveland, Ohio, USA
[5]Department of Cardiac Medicine and Radiology, Oslo University Hospital, Ullevål, Oslo, Norway

Project leader:

AG Semb, MD, PhD, consultant cardiologist, Leader Preventive Cardio-Rheuma Unit, Department of Rheumatology, Diakonhjemmet Hospital

Co-workers:

1. **Diakonhjemmet sykehus**

Department of rheumatology:

prosjektansvarlig Anne Grete Semb	a-semb@diakonsyk.no/anne.semb@yahoo.no
prosjekt koordinator Anne-Kari Brun	
sonografør Anne Eirheim	a-eirhe@diakonsyk.no
avd. overlege Hilde Berner Hammer	h-hammer@diakonsyk.no
dr Sella provan	s-provan@diakonsyk.no
lege/stipendiat Inger Jorid Berg	inger.berg@diakonsyk.no
prof. Tore Kvien	t.k.kvien@medisin.uio.no
Silvia Rollefstad	scrollef@student.uio.no

2. **Oslo University Hospital (OUS) - Ullevål**

a. ***Institutt for preventive medicine***:

prof. Terje Pedersen	t.r.pedersen@medisin.uio.no
prof. Ingar Holme	Ingar.Holme@oslo-universitetssykehus.no

b. ***Radiologisk avd.:***

prof NE Kløw	Kløw Nils-Einar@oslo-universitetssykehus.no
radiograf Mona Svanteson	Mona.svanteson@oslo-universitetssykehus.no
lege Martin Søkjer	martin.sokjer@uus.no

3. **OUS - Aker**

Sirkulasjonsfysiologisk seksjon - Oslo Vaskulære Senter

prof Einar Stranden	Einar.Stranden@medisin.uio.no
fysiolog Jonny Hisdal	jonny.hisdal@medisin.uio.no

4. **OUS - Rikshospitalet**

a. ***Dep. of Nevrology:***

prof David Russel	David.Russell@medisin.uio.no
stip./dr Kristin Evensen	kristin.evensen@ oslo-universitetssykehus.no

b. ***Dep. of radiology and nuclær medicine:***

stip./dr Kjersti Johnsrud	Kjersti.Johnsrud@oslo-universitetssykehus.no
fysiker Arne Skretting	Arne.Skretting@oslo-universitetssykehus.no
fysiker Ingerid Skjei Knudtsen	Ingerid.Skjei.Knudtsen@oslo-universitetssykehus.no
Head of division, MD, PhD Jan Gunnar Fjeld	jan.gunnar.fjeld@ oslo-universitetssykehus.no

c. ***Intervention Center:***

overlege Per Kristian Hol	Per.Kristian.Hol@oslo-universitetssykehus.no
	23070138/23070137/*Mob.: +47 9300 8044*
fysiker Fredrik Courivaud	'Frederic.Courivaud@rr-research.no'

d. ***Inst. of Internal medical research***:

prof. Bente Halvorsen	Bente.Halvorsen@rr-research.no
seksjonsoverlege Pål Aukrust	pal.aukrust@oslo-universitetssykehus.no
stip./dr Tonje Skarpengland	Tonje.Skarpengland@rr-research.no

5. Oslo Universitet -*Institutt for Medisinske basalfag*

a. Department of Physiology

prof. Guro Valen	guro.valen@medisin.uio.no
lege/stipendiat Petter K. Risøe	p.k.risoe@medisin.uio.no
lege/stipendiat Marthe Bliksøen	martbl@medisin.uio.no

b. Department of Anatomy

Senior reseacher Maria Krudtaa Dahle	m.k.dahle@medisin.uio.no

c. Department of Nutrition

prof. Hilde Nebb	h.i.nebb@medisin.uio.no
stipendiat Peng Lei (ernæring/fysiologi)	pengl@medisin.uio.no

6. International collaborators:

a. USA

Stephen J. Nicholls MBBS PhD FRACP FACC
Assistant Professor of Molecular Medicine
Heart and Vascular Institute
Cleveland Clinic
9500 Euclid Avenue, Mail Code JJ-65
Cleveland OH 44195
Ph: 1-216-444-2678
Fa: 1-216-445-5769
Email: nichols1@ccf.org
Assistant: Jessica Fox, phone 1-216-444-2678,
Email FOXJ5@ccf.org

b. United Kingdom

George D. Kitas, MD, PhD, FRCP
Consultant Rheumatologist
Director of Research and Development
Clinical Director - Ambulatory Medicine
Dudley Group of Hospitals NHS Trust (Teaching)
Russells Hall Hospital , North Block, Clinical Research Unit

Dudley, West Midlands DY1 2HQ
United Kingdom
Tel. No. +44-1384-244842 (PA: Mrs Linda Bull)
Fax No. +44-1384-244808
E.mail: gd.kitas@dgoh.nhs.uk or g.d.kitas@bham.ac.uk

- Professor of Clinical Rheumatology,
  Epidemiology Unit, University of Manchester
- Professor of Behavioural Rheumatology
  Health and Life Sciences, University of Birmingham
- Professor of Rheumatology and Musculoskeletal Health
  Research Institute in Healthcare Sciences, University of Wolverhampton

c. Hungaria

prof. Zoltán Szekanecz
Head Department of Rheumatology
University of Debrecen
Medical and Health Sciences Center,
Debrecen,
Hungary
Email: szekanecz@gmail.com

Summary

Patients with rheumatoid Arthritis (RA) and Ankylosing Spondylitis (AS) are at greater risk of developing cardiovascular disease. The reason(s) for this have not been well investigated, but there is a general understanding that systemic inflammation plays a part in the increased cardiovascular morbidity and mortality. In spite of the increased risk in these patients, they have only recently been included as a high risk patient group in cardiovascular prevention guidelines.

We have carried out a cardiovascular study of RA and AS patients, as well as patients with arthritis for the first time. We have demonstrated cholesterol plaques in the carotid artery in some of these patients. Plaques in the carotid artery represent a risk for development of cerebral stroke and are significantly associated with myocardial infarction. These plaques, which are asymptomatic and do not cause haemodynamically significant narrowing, diameter reduction (i.e. operation is not indicated), are vascular atheromatous disease. Therefore, according to prevailing cardiovascular guidelines (SCORE 2007), these patients shall have secondary prevention with a lipid lowering agent with the LDL-cholesterol goal of 1.8 mmol/L and HDL-cholesterol ≥ 1.0 mmol/L for men and ≥ 1.1 mmol/L for women.

Statins are cholesterol-lowering drugs, and have been shown to reduce the risk of cardiovascular disease significantly. In addition, reduction in the size of coronary plaques has been induced by statins, when the LDL has been reduced to 1.6-1.8 mmol/l. Plaques in the carotid or coronary arteries have not previously been treated and characterised in patients with RA, AS and other inflammatory forms of arthritis.

The aim of this study is to treat patients with cholesterol plaques in the carotid artery with cholesterol-lowering medication, in the form of Rosuvastatin for 18 months, and characterise the effects on the plaques in the carotid and coronary arteries. In addition, we want to clarify the connection between plaques in the carotid and coronary arteries in patients with RA, AS and other inflammatory forms of arthritis.

Background

Patients with Rheumatoid Arthritis (RA) and Ankylosing Spondylitis (AS) are at a greater risk of developing cardiovascular disease.[1-4] The increased risk cannot be explained by traditional risk factors for cardiovascular disease.[1,3,5] It is generally accepted that systemic inflammation plays a part in the increased cardiovascular morbidity and mortality. In spite of this, these patients are only now included as a high risk patient group in the cardiovascular prevention guidelines (CV). (ref)

In addition to a risk of cardiovascular disease that is about 3 times higher than in the general population, RA patients more often have silent angina pectoris and silent myocardial infarction, which in addition is more often fatal than in the general population.[2] It is therefore important to establish markers for subclinical arteriosclerosis, which can predict CV risk and future CV events.

The intima media thickness (IMT) in the carotid artery, measured by B mode ultrasound, is a useful non-invasive surrogate marker for general atherosclerosis and coronary disease [6]. This can provide early information about atherosclerosis in the subclinical phase in high risk individuals.[7] Gonzalez-Juanatey has recently reported that IMT of the carotid is a good predictor of the development of CV events in patients with RA without known CV disease and without traditional CV risk factors.[8] In this study, cholesterol plaques were found 5 times as often in those who developed a CV event. Carotid plaques are a strong indicator of future CV events and represent the presence of advanced atherosclerosis.[9]

Statin medication is obligatory in CV disease, and reduction of LDL to1.6-1.8 mmol/L with statins has resulted in a significant reduction of CV events.[10,11] This low LDL level is necessary to reduce the progression of the atheroma, reduce the total burden of the atheroma (total amount of cholesterol deposited) and induce atheroma volume regression in the coronary arteries.[12] In a total of 1455 patients treated with high dose statin, so that LDL cholesterol was reduced to 1.6 – 1.8 mmol/l, a regression of the cholesterol plaques in the coronary arteries has been demonstrated. In the ASTEROIDE trial[13], 40 mg Rosuvastatin reduced the volume of coronary atheroma, while the same medication did not reduced IMT in the METEOR study[14].The latter study included patients with a low CV risk. This will be in contrast to our patient population who has a high risk of CV disease.

Cholesterol plaques in the carotid or coronary arteries have not previously been characterised or treated in patients with Rheumatoid Arthritis, Ankylosing Spondylitis and other inflammatory forms of arthritis. Furthermore, to our knowledge, there is no report on the association between carotid plaques and coronary plaques in these patients.

We have carried out B mode ultrasound imaging of the carotid artery and broad CV risk stratification in 150 patients with RA and 160 patients with AS, as well as some patients

with arthritis for the first time, in a cross section analysis of the cardiovascular status. During this investigation, we have visualized cholesterol plaques in the carotid artery in some of these patients.

Asymptomatic cholesterol plaques in the carotid artery, which do not cause haemodynamically important narrowing of the diameter (i.e. operation is not indicated), are not included in the guidelines for prophylaxis of cardiovascular disease. By characterising cholesterol plaques in the carotid artery, and investigating the association between them and plaques in coronary arteries, it will be possible to evaluate whether a non-invasive scan, such as B mode ultrasound imaging of the carotid artery with plaque evaluation, can be used as part of risk factor evaluation for future CV events in patients with RA, AS and first time arthritis.

Rosuvastatin is water soluble and therefore comparable with pravastatin. Rosuvastatin is not metabolised via cytochrome P450 CYP3A4, but is taken up in the liver slightly more than pravastatin. The clinical relevance of the fact that Methotrexate uses the same transport system (OATP) (shown in isolated cell experiments) is not known.[15,16] Furthermore, this transport system (OATP) has broad substrate specificity and transports bile acids, sulphates, glucoronised conjugates, thyroid hormones, peptides, and medicinal products such as Rosuvastatin, Methotrexate and rifampicin.

In the TARA study,[17] there were more patients who also took Methotrexate who were allocated to the group receiving Atorvastatin than those who received placebo. A larger number of patients who received Atorvastatin remained until the end of the study than those who received placebo, indicating positive effects of Atorvastatin as well as good tolerability of the treatment. Atorvastatin also had the same frequency of adverse effects as the placebo in this study. In particular, there was no significant increase in liver or muscle enzymes in patients treated with Atorvastatin.

Taking the safety data of Rosuvastatin into consideration, in 4 large prospective, placebo controlled, multicentre, international clinical studies, 10 mg Rosuvastatin was used daily in the Corona Study [18] , 20 mg Rosuvastatin in the JUPITER study[19], and 40 mg Rosuvastatin was used in both the METEOR[14] and ASTEROIDE[13] studies. There was no increase in adverse effects or other safety parameters in those who received Rosuvastatin compared to placebo in any of these studies. Considering available data and clinical experience, there is no reason to expect serious adverse effects or events when treating RA patients with Rosuvastatin.

In addition to reducing CRP and having an anti-inflammatory effect in patients with RA, statins seem to have the same lipid-lowering effect as in the general population. In the TARA study [17] the lipid reductions were: Total-cholesterol (T-chol): 32 % and LDL: 49 %.

Van Doornum[20] has reported a reduction of LDL by 42.4% on 20 mg Atorvastatin, while 40 mg Simvastatin[21] reduced the total cholesterol by 21% and LDL by 33.3%. In the IDEAL study, there was a reduction of all the lipids immediately after use of 80 mg Atorvastatin or 20-40 mg Simvastatin in patients who had had myocardial infarction, whether or not they had RA (Semb, submitted data). This in spite of the fact that RA patients had significantly lower total cholesterol and LDL than those who did not originally have RA.

Rosuvastatin in our planned study will be given as well as other RA medication, which will be registered. Special caution will be shown when the patients are taking warfarin or other vitamin K antagonists, as well as antacids.

Objective of the study

1. Evaluate cholesterol plaque regression and plaque characteristics in the carotid and coronary arteries after reduction in LDL cholesterol to 1.6-1.8 mmol/l during 18 months treatment with 20 mg Rosuvastatin, possibly titered up to 40 mg, in patients with Rheumatoid Arthritis, Ankylosing Spondylitis and other inflammatory joint diseases.
2. Evaluate the association between cholesterol plaques in the carotid artery (assessed using B mode ultrasound), and in the coronary arteries, assessed by CT, in patients with Rheumatoid Arthritis, Ankylosing Spondylitis or other inflammatory joint diseases.

Recruiting procedure

1) The patients have all had asymptomatic cholesterol plaques in the carotid artery demonstrated by a previous examination carried out by the project leader, Dr Semb and Anne Eirheim, ultrasonographer, at the Rheumatology Outpatient Clinic. These patients will be contacted and asked whether they are interested in taking part in the investigation, and they will be given an opportunity of meeting the project leader and project nurse at the Rheumatology Outpatient clinic for more detailed information. These patients have also been examined as regards joints, activity of their disease, health status, and stiffness and previous endothelial function of their arteries. An ECG has been taken and their BP measured, and they have also answered a “heart” questionnaire with a depth interview regarding cardiovascular symptoms and risk factors. All the patients who will be invited to take part in the study have gone through the procedures described in the screening procedure in the section "Study design".
2) At the information meeting the patients will receive oral and written information with the possibility of signing a declaration of informed consent to take part in the study. The patients will be given a signed copy of the informed consent. The patients will also be informed that if they do not wish to take part in the study, this will have no

consequences for them. That is, they will have the same access to examination, evaluation, and follow-up by a rheumatologist and a cardiologist. The advantage of taking part in the study is that they receive a more thorough examination and follow up of their cardiovascular status than usually occurs in outpatient consultations.

Study design

Participants in the study will visit the Rheumatology Outpatient Clinic at Diakonhjemmet Hospital for one whole day (or two half days) and receive a full clinical examination, consisting of:

Measurement of disease activity: The participants answer questionnaires and their disease activity will be evaluated and measured, using the routine procedures for following up patients with rheumatic diseases (see Appendices 1a and 1b).

Measurement of health status: The participants answer questionnaires (see Appendices 1a and 1b).

We will also focus especially on ***assessments of possible pathology related to the heart and blood vessels.***

- Questionnaire related to factors that may be related to the occurrence of cardiovascular disease in the patient or in his/her family
- Blood tests (about 20 ml)
- ECG
- Examination of the finger tip by non-invasive peripheral arterial tone (PAT) (ITAMAR medical)
- Measurement of blood pressure, -stiffness and –flow in the radial and femoral artery. Pressure waveform measurements are obtained non-invasively by applanation tonometry.
- Ultrasound of the carotid artery with focus on atherosclerotic plaques obtained non-invasively.
- Transthoracic echocardiogram with doppler.
- Coronary CT for evaluation of atherosclerotic plaques in the coronary arteries.

Patients with atherosclerotic plaques in the coronary arteries will be offered:

- Coronary angography at Ullevål University Hospital, Oslo. The examination will include intra-vascular ultrasound (IVUS)

Patients who have signed an informed consent form will start to take 20 mg Rosuvastatin, 1 tablet a day. Patients aged over 70 years will be given an initial dose of Rosuvastatin of 5 mg, 1 tablet a day. The dose of Rosuvastatin is titered up every 14 days until an LDL level of 1.6-1.8 mmol/l has been reached. In participants aged below 70 years, the dose will be titered up to 40 mg x 1, if an LDL concentration of 1.6-1.8 mmol/l has not been reached after 14 days. In participants aged over 70 years, the titering up will consist of doubling the dose every 14 days until a concentration of LDL of 1.6-1.8 mmol/l has been reached, i.e. 10 mg x 1, 20 mg x 1, and 40 mg x 1. The objective is that all the participants should have reached 1.6-1.8 mmol/l 3 months after the start of the study. The participants will remain on Rosuvastatin medication for a total of 18 months.

**Location**	**Procedure**	**Screening**	Baseline **Visit 1**	Week 2-4-6 **Visit 2-3-4**	Month 3 **Visit 5**	Month 6 **Visit 6**	Month 12 **Visit 7**	Month 18 **Visit 8**
DHH	Blod prøver	X	X	X	X	X	X	X
DHH	Crestor 20mg X 1, (5mg x1 if >70 years)		start	Evt. opptitrering av Crestor dosen	Evt. opptitrering av Crestor dosen			
DHH	Assessment of rheumatic disease activity		X					X
DHH	Questionnaires concerning<br>1) CVD<br>2) Quality of life		X		X			X
DHH	ECG	X	X		X			X
DHH	Blood samples		X		X			X
DHH	Carotid ultrasound	X	X		X			X
DHH	transthoracic echocardiogram	X	X		X			X
DHH	Blood pressure	X	X		X			X
DHH	Vascular biomarkers		X					X
DHH	Ultrasound examination of joints		X					X
DHH	Flow mediated dilation		X					X

UUS	(Coronary angiographyt with IVUS)		X					X
RUS	PET scan, MRI art carotis		X					X
DHH	Recording of adverse events			X	X	X	X	X

DHS: Diakonhjemmet Hospital, Oslo, Norway; UUS: Ullevål University Hospital, Oslo, Norway; RUS: Rikshospitalet University Hospital, Oslo, Norway.

All the results will be unidentified before they are analyzed/processed blinded to the analyser/processor. The clinical relevant data will also become part of the patient's case record. In this way, the results will represent a status that can be used for later evaluation of whether the health condition has been changed.

Inclusion criteria

1. Women and men with RA, AS and other inflammatory forms of arthritis, aged 35-80 years.
2. Cholesterol plaques demonstrated in carotid artery by ultrasound.
3. Informed consent.

Exclusion criteria

1. Concomitant statin treatment
2. Contraindication to statin treatment.
    - Hypersensitivity to statins
    - Liver disease with ASAT/ALAT ≥ twice the upper normal limit
    - Previous statin-induced myopathy or severe hypersensitivity reactions to other statins
    - Raised creatinine (because of contrast medium)
    - Pregnancy or breast feeding
    - Fertile women who do not use contraceptives
    - Cyclosporine treatment
    - Treatment with medicinal products that have a known interaction with Rosuvastatin

- Uncontrolled hypothyroidism defined as TSH > 1.5 times ULN at the first visit (because of the connection between myopathy and hypothyroidism with statin treatment)
- Creatinine clearance < 30 ml/min and <60 ml/min with a Rosuvastatin dose of 40 mg per day

3. Secondary hyperlipidemia
    - Primary hyperthyroidism
    - Nephrotic syndrome, creatinine > 2 mg/dl
    - Uncontrolled diabetes mellitus (HbA1C > 10 %)
    - Plasma Triglycerides > 6.8 mmol/l
4. Other diseases or treatment that reduces the safety, or treatment with Rosuvastatin which would interfere with the end points of the study
    - Heart failure: NYHA class III B/IV
    - Haemodynamically significant valve defects
    - Established statin treatment
    - Gastrointestinal disease/treatment that can give malabsorption of Rosuvastatin
    - Cancer
    - Severe psychiatric disease
    - Life-threatening ventricular arrhythmias
    - Other medication that increases the risk of rhabdomyolysis
    - Known abuse of alcohol
    - Participation in other studies

Study procedures

1. Blood tests:

20 ml venous blood will be taken at the times shown in the “flow chart”. The following parameters will be analysed in blood:

- Creatine kinase, ASAT, ALAT
- Hb, Hct, SR, CRP, white blood cells, thrombocytes, electrolytes, proBNP, creatinine, GFR, uric acid,
- Lipid status: total cholesterol, HDL, TG, LDL, Apolipoprotein B, Apolipoprotein A-1, Apolipoprotein (a)
- Blood samples for analysis of biomarkers/Rheumatoid Factor/anti-CCP

In addition, a uristix test will be carried out at the same time

2. ***Disease activity measurement as regards joint disease:*** The participants answer questionnaires and their disease activity will be measured, as used in the routine follow-up of patients with rheumatic diseases (see Appendices 1a and 1b).

   a. 28 joint count (entered by a doctor or nurse in Go Treat It (GTI), which is an IT based reporting tool.
   b. The investigator's total assessment of the disease activity (entered in GTI by the person carrying out the evaluation).
   c. BASDAI (patient's self report in GTI).

3. ***Health status:*** The participants answer questionnaires (see Appendices 1a and 1b).

   1. MHAQ (patient's self report in GTI)
   2. BASFI (patient's self report in GTI)
   3. Pain, fatigue, morning stiffness, patient's total assessment (patient's self reporting in GTI)
   4. Examination of disease activity of the joint disease

4. ***Questionnaire on cardiovascular symptoms, diseases and risk factors*** (see Appendix 2)

5. Exination of peripheral vascular function and morphology:

a. Peripheral arterial tone (PAT) technology (ITAMAR medical) permits non-invasive evalutation of the cardiovascular system and autonomic nervous system. The PAT signal is a proprietary technology used for non-invasively measuring arterial tone changes in peripheral arterial beds. The PAT signal is measured from the fingertip by recording finger arterial pulsatile volume changes. Based on PAT Technology, the noninvasive EndoPAT system comprises a measurement apparatus that supports a pair of modified plethysmographic bio-sensors.
b. Measurement of arterial pressure, -stiffness and –flow can be obtained by applanation tonometry at the radial and femoral artery. The measurement is performed non-invasively with a probe that is applied gently to the skin. The Sphygmacor apparatus will be utilized. Measurement of the blood flow in the brachial artery after 5 min occlusion by a blood pressure cuff that is inflated. Radial artery diameter is measured before and after the occlusion by ultrasound measurements.

6. ***B mode ultrasound imaging of carotid artery:*** Standardized examination with recorded angles of turning the head in relation to the mid-line of the body and the angle of the probe to

the neck. Intima media thickness is measured in the common carotid artery (CCA) 1 cm above the defined start of the carotid bulb, covering 1 cm. Images will also be taken of plaques in the CCA, bulb and internal carotid artery. The following will be assessed:

1) ***Plaque height and area.*** The plaque height will be measured as the height from the leading edge of the atherosclerotic protrusion in to the lumen to the leading edge of the media. Ultrasound measurements will be evaluated by the Artery Measurement System (AMS) program. The line of the atherosclerotic height will be drawn perpendicular to the artery wall. One will make sure that there is a sharp image of the IMT both in the far wall and the near wall at both baseline and at the 18 months visits, in addition to ensure that the images are similar and from the same segment. Digital standardized plaque area assessments as described by Gray-Weale and Nicolaides,[24] and Nicolaides[25], where we will use the AMS program[26]
2) ***Plaque morphology.***
    a. Plaque class: AMS program (Artery Measurement System)
    b. Gray Scale Median value (AMS program)[24-26]
3) ***Intima media thickness*** assessed by AMS program.

7. **Transthoracic echocardiogram:** To be performed by standardized recordings and measurements with 2D, M-Mode and Doppler (Appendix 3).

8. **Multidetector CT (MDCT) angiography:** MDCT of the heart for detection of coronary atherosclerotic plaques will be performed at Ullevål University Hospital in which prof NE Kløw will be the resposible project leader. The MDCT angiography method is described in more detail in appendix 4. One will evaluate:
    1. Coronary calcium score (CCS)
    2. Coronary atherosclerotic plaques
    3. Coronary stenses

    The MDCT angiography has three possible outcomes:
    1. Normal coronary arteries
    2. Coronary plaques
    3. Coronary stenosis

The analyses of the MDCT angiography will be performed at Ullevål University Hospital.

9. **Coronary angiography and intravascular ultrasound (IVUS)**

(The procedure is attached as appendix 5). This procedure will be performed at the Dept. Cardiology / Radiology at Ullevål University Hospital, Oslo in which prof. NE Kløw will be the responsible project leader.The analyses of the IVUS data will be performed at the Cleveland Clinic in which prof. S. Nicholls will be the resposible project manager. If the conclusion of the CT examination is coronary stenosis, suspicion of coronary stenosis or poor image quality, the patients will be offered selective coronary angiography with IVUS. The atherosclerotic burden will be assessed and the patients will be offered a follow-up selective coronary angiography or MDCT after 18 month rosuvastatin therapy (at study end).

The patients will not receive interventions as a part of the coronary angiography procedure. However, the patients will be continously and individually monitored with regards to the necessity of this intervention.

Analyses of the IVUS data will be performed at the Cleveland Clinic

Primary end points:

Carotid artery plaques: Reduction of plaque height, area (GSM), volume (MRI) and change of the plaque morphology to less vulnerable for rupture after 18 months with 40 mg Rosuvastatin daily.

Secondary end points:

The effect of 18 months treatment with Rosuvastatin on:

1. **Disease activity and Health status**
    i. **Disease activity** will be measured by: 28-swollen-joint count, AIMS2, BASDAI
    ii. **Health status** will be measured by MHAQ, BASFI, Pain VAS, Fatigue VAS, life quality (HRQoL)
2. **Lipoprotein components:** Lipids, apolipoproteins, magnitude and functional measurements of these, for example of HDL
3. **Vascular and other biomarkers/inflammation parameters**

Tertiary end points:

1. **Coronary artery plaques:** Change in the area or volume of coronary plaques after 18 month rosuvastatin therapy as measured by CT and IVUS in patients with coronary plaques at the start of the study.

Handling the medicinal product

All the study medication will be provided by AstraZeneca. This includes production, packing and issuing. Dispensing to the patient and storing of the study medication will be carried out by the Hospital Pharmacy.

Compliance

The patient will be given the necessary number of tablets by the pharmacy at each visit. At the following visits, surplus medication will be returned to the investigator. The compliance is calculated as a percentage from the number of tablets returned. More than 80 % compliance is required for the patient to be included in the per-protocol group.

Data collection

All the data collection will be recorded in the case report forms (CRF), which are written independently of normal case records according to GCP. Quality control of collected data will be carried out internally at The Department of Rheumatology by the study personnel.

Reporting adverse effects

Registration of adverse events (AE) will be carried out at each visit, and the patient will also be encouraged to make direct contact with the person responsible for the project or the study nurse and tell them if an adverse effect should arise. The patients will be interviewed regarding adverse effects by using questions such as: "Have you had any health problems since your last visit?" The data will be documented in CRF and reported in accordance with the guidelines. The investigator will report to The Norwegian Medicines Agency (SLV) (with a copy to AstraZeneca) as quickly as possible, but within 7 days if unexpected severe adverse events are suspected (SUSAR), and within 15 days if unexpected adverse events are suspected once the investigator has gained knowledge of the event.

Study size, statistical analyses and duration of study

A 4.2% reduction of the plaque area (carotid artery) compared with the baseline can be expected after treatment with Rosuvastatin for 2.5 years compared with a placebo group.[27] Several intravascular ultrasound studies have shown that reduction of LDL with statins delays the progression of atherosclerosis and can induce regression[12] after 18 months statin treatment, if the LDL level is reduced to 1.6-1.8 mmol/L.

Patients with cholesterol plaques in the carotid artery have vascular atherosclerotic disease and, according to Norwegian and international guidelines (both European and

American), they should have secondary prophylactic treatment where the desired LDL level is 1.8 mmol/L. In view of this, it is unethical to include a placebo group in this study.

The study requires 75 patients to detect a change of 4.2 % area with SD= 13.0 % with 80 % safety at the 5 % level of significance. The reality of a standardized effect of 3.1 seems reasonable in view of the results of the ASTEROID study, which found a net difference of 3.25 SD units between the treatment and placebo groups. This will then have 80% power for detecting a 4.2 % reduction in plaque area in the carotid artery in 100 patients, if the standard deviation (SD) of the percent change from start to end of the study (18 months treatment) is less than 3.1 times the mean percent reduction. An interim check of the SD after half of the study period has elapsed will be carried out to see whether the assumption (Ratio SD/effect=3.1) is satisfactory. Missing data will be handled as follows: Patients who do not follow the protocol completely will be included in the intention-to-treat analysis group (ITT). Examples of cases where this may be relevant include compliance below 80 %, protocol irregularities, failure to attend visits, etc. If patients drop out of the study, the last observation carried forward (LOCF) technique will be used in the intention-to-treat analysis of these patients. Patients who follow the protocol completely will be included in the per-protocol analysis.

One hundred patients will be recruited continuously from on-going studies and followed prospectively, in an open, blinded evaluation of the end points study design, where plaque evaluation in both the carotid and coronary arteries will be carried out blinded as regards both patient identification and investigation sequence. The following will be used:

- Paired examinations to compare changes in the plaques, lipids and secondary a nd tertiary end points.
- Bivariable examinations of associations between changes in plaques and secondary variables.
- Longitudinal analyses (demographic variables in "generalized estimating equations) (GEE) to examine longitudinal independent associations between plaques and secondary end points.
- Skew distributed variables will be examined after logarithmic transformation.
- We will use "intention to treat" analysis for patients who do not follow the protocol completely, and the last-observation-carried-forward method for calculating the end point.

Ethical assessments

The study will be carried out according to The Helsinki Declaration (WMA, 1964), and approval has been applied for from the Regional Ethical Committee (REK). The patients will be given written information about this in the Information sheet for participation in the project.

The patient population to be investigated are initially a high risk population with 2-3 times higher risk of developing a CV event than the general population. There will therefore be a clinical indication for invasive cardiovascular examination of these patients. There is no knowledge of the association between carotid artery plaques and coronary plaques in patients with inflammatory joint disease. Initially, all the participants in the study will be offered MCT coronary angiography. A MCT scan is reported to be of high diagnostic safety in diagnosing coronary stenosis in 50 % of patients with chest pain but no known cardiovascular disease.[22;23] Patients in whom coronary plaques and stenoses are demonstrated are referred for coronary angiography, which includes intravascular ultrasound imaging (IVUS).

Termination of participation in the study

1. Withdrawal of informed consent.
2. Each time that CK is found to be >5 x ULN (upper limit of normal) and/or is accompanied by muscular discomfort/pain/soreness and/or serious weakness, even when the CK is <5 x ULN.
3. Persistent ASAT/ALAT >3 x ULN at two measurements with at least 48 hours in between.
4. If the patient's health deteriorates, as assessed by the investigator, who requires the study medication to be discontinued.
5. If an adverse effect develops that the investigator considers to make it necessary to discontinue the study medication.
6. Pregnancy.
7. If the investigator considers this to be necessary.

Insurance

All the patients will be insured via Medical Product Liability Insurance.

Ethics and concession from the Data Inspectorate and Biobank

The Regional Committee for Medical Research Ethics, the personal safety deputy, the Norwegian medicines Agency and The Norwegian Data Directorate have approved of the

protocol. The study has been approved for a BioBank and has been registered in clinicaltrials.gov

Progress – milestones – responsibility

This study has been initiated by the investigators, and the investigators have all the rights to the data material and publication rights of these.

	Data collection:
January 2010	First patient in
July 2012	Last patient in
December 2013	Last patient visit
January-March 2014	Data processing, analysis, and publication of the material

Reference List

(1) Del Rincon I, Williams K, Stern MP, Freeman GL, Escalante A. High incidence of cardiovascular events in a rheumatoid arthritis cohort not explained by traditional cardiac risk factors. Arthritis Rheum 2001; 44(12):2737-2745.

(2) Maradit-Kremers H, Nicola PJ, Crowson CS, Ballman KV, Gabriel SE. Cardiovascular death in rheumatoid arthritis: a population-based study. Arthritis Rheum 2005; 52(3):722-732.

(3) Maradit-Kremers H, Crowson CS, Nicola PJ, Ballman KV, Roger VL, Jacobsen SJ et al. Increased unrecognized coronary heart disease and sudden deaths in rheumatoid arthritis: a population-based cohort study. Arthritis Rheum 2005; 52(2):402-411.

(4) van Halm V, Peters MJ, Voskuyl AE, Boers M, Lems WF, Visser M et al. Rheumatoid arthritis versus diabetes as a risk factor for cardiovascular disease, a cross sectional study. The CARRE Investigation. Ann Rheum Dis 2008.

(5) Dessein PH, Joffe BI, Veller MG, Stevens BA, Tobias M, Reddi K et al. Traditional and nontraditional cardiovascular risk factors are associated with atherosclerosis in rheumatoid arthritis. J Rheumatol 2005; 32(3):435-442.

(6) Simons PC, Algra A, Bots ML, Grobbee DE, van der GY. Common carotid intima-media thickness and arterial stiffness: indicators of cardiovascular risk in high-risk patients. The SMART Study (Second Manifestations of ARTerial disease). Circulation 1999; 100(9):951-957.

(7) Pignoli P, Tremoli E, Poli A, Oreste P, Paoletti R. Intimal plus medial thickness of the arterial wall: a direct measurement with ultrasound imaging. Circulation 1986; 74(6):1399-1406.

(8) Gonzalez-Juanatey C, Llorca J, Martin J, Gonzalez-Gay MA. Carotid Intima-Media Thickness Predicts the Development of Cardiovascular Events in Patients with Rheumatoid Arthritis. Semin Arthritis Rheum 2008.

(9) Roman MJ. Preclinical carotid atherosclerosis in patients with rheumatoid arthritis. . Moeller E, Davis A, Paget SA, Crow MK, Lockshin MD, editors. Ann Intern Med 144, 249-256. 2006.
Ref Type: Generic

(10) Pedersen TR, Faergeman O, Kastelein JJ, Olsson AG, Tikkanen MJ, Holme I et al. High-dose atorvastatin vs usual-dose simvastatin for secondary prevention after myocardial infarction: the IDEAL study: a randomized controlled trial. JAMA 2005; 294(19):2437-2445.

(11) LaRosa JC, Grundy SM, Waters DD, Shear C, Barter P, Fruchart JC et al. Intensive lipid lowering with atorvastatin in patients with stable coronary disease. N Engl J Med 2005; 352(14):1425-1435.

(12) Nicholls SJ, Tuzcu EM, Sipahi I, Grasso AW, Schoenhagen P, Hu T et al. Statins, high-density lipoprotein cholesterol, and regression of coronary atherosclerosis. JAMA 2007; 297(5):499-508.

(13) Nissen SE, Nicholls SJ, Sipahi I, Libby P, Raichlen JS, Ballantyne CM et al. Effect of very high-intensity statin therapy on regression of coronary atherosclerosis: the ASTEROID trial. JAMA 2006; 295(13):1556-1565.

(14) Crouse JR, III, Raichlen JS, Riley WA, Evans GW, Palmer MK, O'Leary DH et al. Effect of rosuvastatin on progression of carotid intima-media thickness in low-risk individuals with subclinical atherosclerosis: the METEOR Trial. JAMA 2007; 297(12):1344-1353.

(15) Nezasa K, Higaki K, Takeuchi M, Nakano M, Koike M. Uptake of rosuvastatin by isolated rat hepatocytes: comparison with pravastatin. Xenobiotica 2003; 33(4):379-388.

(16) Marzolini C, Tirona RG, Kim RB. Pharmacogenomics of the OATP and OAT families. Pharmacogenomics 2004; 5(3):273-282.
(17) McCarey DW, McInnes IB, Madhok R, Hampson R, Scherbakov O, Ford I et al. Trial of Atorvastatin in Rheumatoid Arthritis (TARA): double-blind, randomised placebo-controlled trial. Lancet 2004; 363(9426):2015-2021.
(18) Kjekshus J, Apetrei E, Barrios V, Bohm M, Cleland JG, Cornel JH et al. Rosuvastatin in older patients with systolic heart failure. N Engl J Med 2007; 357(22):2248-2261.
(19) Ridker PM, Danielson E, Fonseca FA, Genest J, Gotto AM, Jr., Kastelein JJ et al. Rosuvastatin to Prevent Vascular Events in Men and Women with Elevated C-Reactive Protein. N Engl J Med 2008.
(20) Van Doornum S., McColl G, Wicks IP. Atorvastatin reduces arterial stiffness in patients with rheumatoid arthritis. Ann Rheum Dis 2004; 63(12):1571-1575.
(21) Hermann F, Forster A, Chenevard R, Enseleit F, Hurlimann D, Corti R et al. Simvastatin improves endothelial function in patients with rheumatoid arthritis. J Am Coll Cardiol 2005; 45(3):461-464.
(22) Budoff MJ, Dowe D, Jollis JG, Gitter M, Sutherland J, Halamert E et al. Diagnostic performance of 64-multidetector row coronary computed tomographic angiography for evaluation of coronary artery stenosis in individuals without known coronary artery disease: results from the prospective multicenter ACCURACY (Assessment by Coronary Computed Tomographic Angiography of Individuals Undergoing Invasive Coronary Angiography) trial. J Am Coll Cardiol 2008; 52(21):1724-1732.
(23) Ostrom MP, Gopal A, Ahmadi N, Nasir K, Yang E, Kakadiaris I et al. Mortality incidence and the severity of coronary atherosclerosis assessed by computed tomography angiography. J Am Coll Cardiol 2008; 52(16):1335-1343.
(24) Gray-Weale AC, Graham JC, Burnett JR, Byrne K, Lusby RJ. Carotid artery atheroma: comparison of preoperative B-mode ultrasound appearance with carotid endarterectomy specimen pathology. J Cardiovasc Surg (Torino) 1988; 29(6):676-681.
(25) Nicolaides AN, Shifrin EG, Bradbury A, Dhanjil S, Griffin M, Belcaro G et al. Angiographic and duplex grading of internal carotid stenosis: can we overcome the confusion? J Endovasc Surg 1996; 3(2):158-165.
(26) Geroulakos G, Ramaswami G, Nicolaides A, James K, Labropoulos N, Belcaro G et al. Characterization of symptomatic and asymptomatic carotid plaques using high-resolution real-time ultrasonography. Br J Surg 1993; 80(10):1274-1277.
(27) Spence JD, Eliasziw M, DiCicco M, Hackam DG, Galil R, Lohmann T. Carotid plaque area: a tool for targeting and evaluating vascular preventive therapy. Stroke 2002; 33(12):2916-2922.
