# Supplementary material for: Sustained Improvement of Arterial Stiffness and Blood Pressure after Long-Term Rosuvastatin Treatment in Patients with Inflammatory Joint Diseases: Results from the RORA-AS Study
Source: PLoS One. 2016 Apr 19;11(4):e0153440. doi: 10.1371/journal.pone.0153440 (PMC4836743; doi:10.1371/journal.pone.0153440)
Supplement: S2 File — (PDF) [file pone.0153440.s002.pdf]

Kolesterolavleiring i arteria carotis og koronar arterier og effekten av Rosuvastatin ved revmatoid artritt, ankyloserende spondylitt og andre inflammatoriske artritter

RORA-AS studien

Versjon 06.07.11

Eudract nr 2008-005551-20
Sponsor's protocol Code Number 1957
REK 2009/2219
SLV 09/090799-11

AG Semb[1], E Stranden [2], S Provan[1], A Eirheim[1], I Holme[3], TR Pedersen[3], S Nicholls[4], NE Kløw[5], TK Kvien[1]

[1]Revmatologisk avdeling, Diakonhjemmet sykehus,
[2]Sirkulasjonsfysiologisk laboratorium, Oslo Vaskulære Senter, Aker Universitets Sykehus
[3]Avdeling for preventiv Medisin, Ullevål Universitets sykehus
[4]Department of Cardiovascular Medicine, Cleveland Clinic, Cleveland, Ohio, USA
[5]Hjertemedisinsk og Radiologisk avdeling, Ullevål Universitets sykehus

Innhold

Prosjektledelse og samarbeidspartnere 3
Signatur side 4
Sammendrag 5
Prosjektbeskrivelse 6
- Studiens hensikt 8
- Rekrutterings prosedyre 8
- Studie design 9
- Tidsforløp 10
- Inklusjonskriterier 11
- Eksklusjonskriterier 11
- Studieprosedyrer 12
  - Blodprøver 12
  - Sykdomsaktivitetsmål vedr ledd sykdom appendiks 1a +1b
  - Helsestatus appendiks 1a 13
  - Spørreskjema vedr hjertekar -symptomer, -sykdom og -risikofaktorer Appendiks 2 13
  - Undersøkelser vedr perifer blodkars funksjon 13
  - B-Mode ultralyd undersøkelse av arteria carotis 13
  - Transthorakal ekkokardiografi Appendiks 3 14
  - Multidetektor CT koronar angiografi Appendiks 4 14
  - Koronar angiografi og intravaskulær ultralyd (IVUS) Appendiks 5 14

Primære endepunkter 15
Sekundære endepunkter 15
Tertiære endepunkter 15
Legemiddelhåndtering 16
Complience 16
Datainnsamling 16
Rapportering av bivirkninger 16
Studiestørrelse, statistiske analyser og studiens varighet 16
Etiske vurderinger 17
Avslutning av deltagelse i studiet 18
Forsikring 19
Etikk og konsesjon av Datatilsynet og Biobank 19
Fremdrift - milepæler - ansvarsforhold 19
Referense liste 20

Appendiks 1a +b 22
Appendiks 2 43
Appendiks 3 49
Appendiks 4 50
Appendiks 5 51
Appendiks 6 53
Appendiks 7 55

Endringsprotokoller FMD 56
- Ultralyd ledd 57
- MRI art. carotis og cerebrum, FDG-PET scan 58

Pasient informasjon - 1 61
Pasient informasjon - 2 67

Prosjektleder:

AG Semb, overlege dr med, senior forsker og hjertespesialist ved revmatologisk avd., Diakonhjemmet sykehus

Samarbeidspartnere:

1. S Provan, lege, stipendiat, Revmatologisk avdeling, Diakonhjemmet sykehus
2. A Eirheim, prosjektsykepleier og ultralyd sonografør, Revmatologisk avd., Diakonhjemmet sykehus
3. E Stranden, professor, Sirkulasjonsfysiologisk laboratorium, Oslo Vaskulære Senter, Aker Universitets Sykehus
4. Ingar Holme, professor, statistiker, Avdeling for Preventiv Medisin, Ullevål Universitets Sykehus
5. NE Kløw, professor, avd. sjef, M Brekke, overlege, Radiologisk avdeling og O Johansen, overlege, Hjertemedisinsk avdeling, Ullevål Universitets Sykehus
6. TR Pedersen, professor/avdelingssjef, Avdeling for Preventiv Medisin, Ullevål Universitets Sykehus
7. TK Kvien, professor/avdelingssjef, Revmatologisk avdeling, Diakonhjemmet sykehus
8. S Nicholls, Department of Cardiovascular Medicine, Cleveland Clinic, Cleveland, Ohio, USA
9. Professor Guro Valen, Fysiologisk Institutt, Universitet i Oslo
10. Professor Hilde Nebb, Ernæringsfyiologisk institutt, UiO
11. Professor David Russel, Nevrologisk Institutt, Oslo Universitets Sykehus - Rikshospitalet

Signaturside

Dato/sted:________________ Dato/sted:______________

Signatur:________________ Signatur:________________

A.G. Semb E. Stranden

Dato/sted:________________ Dato/sted:________________

Signatur:________________ Signatur:________________

T.R. Pedersen I. Holme

Dato/sted:________________ Dato/sted:________________

Signatur:________________ Signatur:________________

S. Provan T.K. Kvien

Dato/sted:________________ Dato/sted:________________

Signatur:________________ Signatur:________________

A. Eirheim N.E. Kløw

Diakonhjemmet Sykehus

Kort sammendrag

Pasienter med inflammatorisk leddsykdom har forhøyet risiko for hjerte-kar sykdom. Vi har identifisert kolesterolavleiringer (plakk) i halsens pulsåre (arteria carotis) hos pasientene med inflammatorisk leddsykdom. Arteria carotis plakk representerer en risiko for hjerneslag og er signifikant assosiert med hjerteinfarkt. Slike plakk, er å oppfatte som kolesterolavleirings sykdom. I følge gjeldende retningslinjer for forebygging av hjerte-kar sykdom skal disse pasientene ha lipidsenkende behandling med mål for LDL på 1.8 mmol/L. Statiner er kolesterolsenkende medikasjon og er vist å signifikant redusere risikoen for hjerte-karsykdom. I tillegg har reduksjon av størrelsen på koronare plakk vært indusert av rosuvastatin, når LDL har vært redusert til 1.8 mmol/l i 18 mnd. Vi ønsker å karakterisere sammenhengen mellom plakk i arteria carotis og koronare arterier hos pasienter med inflammatoriske leddsykdom. I tillegg vil vi behandle pasienter med kolesterolavleiring i halsen pulsåre med kolesterolsenkende medisin i form av rosuvastatin i 18 måneder, og karakterisere effektene på plakkene i arteria carotis og koronare arterier

Sammendrag

Pasienter med Revmatoid Artritt (RA) og Behkterew har en forhøyet risiko for hjerte-kar sykdom. Årsaken(e) til dette er ikke godt kartlagt, men det er en generell forståelse av at den systemiske betennelsen er delaktig i den forhøyete hjerte-kar sykeligheten og dødeligheten. Til tross for den forhøyete kardio-vaskulære risiko hos disse pasientene, finnes det ikke retningslinjer for hjerte-kar prevensjon for dem.

Vi har utført en hjerte-kar undersøkelse av RA og Behkterew pasienter, samt pasienter med førstegangs artritt. I denne forbindelse har vi avdekket kolesterolavleiringer (plakk) i halsens pulsåre (arteria carotis) hos noen av pasientene. Arteria carotis plakk representerer en risiko for hjerneslag og er signifikant assosiert med hjerteinfarkt. Slike plakk, som er asymptomatiske, og som ikke gir hemodynamisk betydningsfulle forsnevringer i form av diameter reduksjon (dvs. skal ikke opereres), er å oppfatte som kolesterolavleirings sykdom. Disse pasientene skal derfor i følge gjeldende retningslinjer for forebygging av hjerte-kar sykdom skal ha primær prevensjon. Med andre ord, de skal ha lipidsenkende behandling med mål for LDL-kolesterol på 1.8 mmol/L og HDL-kolesterol for menn > 1.0 mmo,l/L, og kvinner > 1.1 mmol/L.

Statiner er kolesterolsenkende medikasjon og er vist å signifikant redusere risikoen for hjerte-karsykdom. I tillegg har reduksjon av størrelsen på koronare plakk vært indusert av rosuvastatin, når LDL har vært redusert til 1.6-1.8 mmol/l i 18 mnd. Plakk i halsens pulsårer eller koronare arterier har ikke tidligere vært behandlet og karakterisert hos pasienter med Revmatoid Artritt, Behkterew og andre inflammatoriske artritter.

Vi ønsker å karakterisere sammenhengen mellom plakk i arteria carotis og koronare arterier hos pasienter med Revmatoid Artritt, Behkterew og andre inflammatoriske artritter. I tillegg vil vi behandle pasienter med kolesterolavleiring i halsen pulsåre som ikke står på statin med kolesterolsenkende medisin i form av rosuvastatin i 18 måneder, og karakterisere effektene på plakkene i arteria carotis og koronare arterier.

Prosjektbeskrivelse

Bakgrunn: Pasienter med Revmatoid Artritt (RA) og Behkterew har en forhøyet risiko for hjerte-kar sykdom. [1-4] Den økte risikoen kan ikke forklares av tradisjonelle risikofaktorer for hjerte-karsykdom.[1;3;5] Det er en allment akseptert generell forståelse at den systemiske betennelsen (inflammasjon) er delaktig i den forhøyete hjerte-kar -sykeligheten og -dødeligheten. Til tross for dette er disse pasientene ikke tatt hensyn til i forebyggende retningslinjer for kardio-vaskulær (KV) sykdom. Verken primær eller sekundær prevensjon.

I tillegg til en ca 3 ganger høyere risiko for hjerte-kar sykdom enn den generelle befolkingen, har RA pasienter oftere stille angina pektoris samt oftere stumme hjerteinfarkter som oftere er fatale, enn den generelle befolkningen.[2] Det er derfor viktig å etablere markører for subklinisk aterosklerose som kan predikere KV risiko og fremtidige KV hendelser.

Intima media tykkelse (IMT) av arteria carotis, målt ved B-mode ultralyd, er en nyttig, ikke invasiv surrogat markør for generell ateromatose og koronar sykdom.[6] Dette kan gi tidlig informasjon om ateromatose i subklinisk fase hos høyrisiko individer.[7] Gonzalez-Juanatey har nylig rapportert at carotis IMT er var en god prediktor for utvikling av KV hendelser hos pasienter med RA uten kjent KV sykdom og uten tradisjonelle KV risikofaktorer.[8] I denne studien var kolesterolplakk 5 ganger hyppigere hos de som utviklet KV hendelse. Carotis plakk er en sterk prediktor for fremtidig KV hendelser og representerer tilstedeværelsen av avansert aterosklerose.[9] Rosuvastatin er rapportert å redusere IMT hos pasienter med hyperkolesterolemi og asymptomatisk arteria carotis sykdom.[10]

Arteria carotis plakk representerer en risiko for hjerneslag og er signifikant assosiert med hjerteinfarkt. Slike plakk, som er asymptomatiske, og som ikke gir hemodynamisk betydningsfulle forsnevringer i form av diameter reduksjon (dvs. skal ikke opereres), er å oppfatte som kolesterolavleirings sykdom. Disse pasientene skal derfor i følge gjeldende retningslinjer for forebygging av hjerte-kar sykdom skal ha primær prevensjon. Med andre ord, de skal ha lipidsenkende behandling med mål for LDL-kolesterol på 1.8 mmol/L og HDL-kolesterol for menn > 1.0 mmo,l/L, og kvinner > 1.1 mmol/L.

Statin medikasjon er obligatorisk ved KV sykdom og reduksjon av LDL til 1.6-1.8 mmol/L med statiner har vist signifikant reduksjon av KV hendelser.[11;12] Dette lave nivået av LDL er nødvendig for å redusere progresjonen av atheromet, redusere den totale byrden av atheromet (totale mengden av kolesterol avleiret) og indusere atheroma volum regresjon i koronare arterier.[13] I til sammen 1455 pasienter behandlet med høydose statin, slik at LDL kolesterol ble redusert til 1.6-1.8 mmol/l, er det vist en regresjon av kolesterolavleiringen i hjertets kransårer. I Asteroide studien[14] reduserte 40 mg rosuvastatin koronart atheroma volum, mens samme medikasjon ikke reduserte IMT i METEOR studien.[15] Sistnevnte studie inkluderte pasienter med lav KV risiko. Dette vil være i kontrast til vår pasient populasjon, som har høy risiko for KV sykdom.

Kolesterolavleiring (plakk) i arteria carotis eller koronar arteriene har ikke tidligere vært karakterisert eller behandlet hos pasienter med Revmatoid Artritt, Behkterew og andre

inflammatoriske artritter. Videre finnes det ikke kunnskap om sammenhengen mellom arteria carotis plakk og koronare plakk hos disse pasientene.

Vi har utført B-mode ultralyd undersøkelse av arteria carotis og bred KV risiko stratifisering av 150 pasienter med RA og 150 pasienter med Behkterew samt noen pasienter med førstegangs artritt i en tverrsnittsanalyse vedr. hjerte-kar status. Disse studiene er avsluttet. Vi har også undersøkt pasienter med inflammatorisk leddsykdom på en forebyggende hjerte-revmatologisk poliklinikk på Diakonhjemmet sykehus. Vi har avdekket asymptomatiske kolesterolavleiringer i arteria carotis hos noen av pasientene. Studiedeltagere vil bli rekruttert blant disse pasientene.

Asymptomatiske kolesterolavleiringer i arteria carotis som ikke gir forsnevringer i form av diameter reduksjon og/eller som ikke er hemodynamisk betydningsfulle (dvs. skal ikke opereres), er ikke tatt med i retningslinjer vedrørende forebygging av hjerte-kar sykdom. Ved å karakterisere kolestreolplakk i arteria carotis og undersøke sammenhengen mellom dem og plakk i koronare arterier, vil man kunne evaluere om den non invasive undersøkelse som B-mode ultralyd av halspulsårer og plakk evaluering vil kunne brukes som ledd i en risiko faktor evaluering for fremtidige KV hendelser hos pasienter med RA, Behkterew og førstegangs artritter.

Rosuvastatin filtreres via nyrene. Definering av nedsatt nyrefunksjon vil derfor være viktig. Kronisk nyresykdom og nyresvikt har vært dårlig definerte begreper, men det foreligger nå internasjonal konsensus om nye definisjoner, hvor det anbefales at nyrefunksjonen ikke beregnes ut i fra creatinine clearance, men glomerulær filtrasjons hastighet (GFR) beregnes ut fra formler basert på plasma kreatinin, basert på Modification of Diet in Renal Disease-studien (MDRD formelen). Ingen av de 100 pasientene som har fått påvist plakk i arteria carotis og som skal inngå i RORA studien har kreatinin (50-90 µmo/L) eller GFR utenfor normal området. Dersom kreatinin stiger og GFR faller til <60 ml/min/1.73 m^2 ved 2 målinger med minst 48 timer mellom når pasienten bruker rosuvastatin i studien, vil pasienten bli tatt ut av studien.

Rosuvastatin er vannløselig, og derfor sammenlignbart med pravastatin. Rosuvastatin metaboliseres ikke via cytokrome P450 CYP3A4, men taes opp i leveren noe mer enn pravastatin. Den kliniske relevansen av at Methotrexate bruker det samme transport (OATP) systemet (vist i isolerte celle forsøk) er ikke kjent.[16,17] Dessuten, har dette transport systemet (OATP) en bred substratspesifisitet og transporterer gallesyrer, sulfater, glukoroniserte konjungater, thyroidea hormoner, peptider, medikamenter som rosuvastatin, Methotrexate, og rifampicin.

I TARA studien,[18] var det flere pasienter som også tok Methotrexate som var allokert til gruppen som fikk Atorvastatin enn de som fikk placebo. Flere pasienter som fikk Atorvastatin var med til studiens slutt enn de som fikk placebo, som indikerer positive effekter av Atorvastatin samt god tolerabilitet av behandlingen. I tillegg, hadde Atorvastatin samme bivirkningsfrekvens som placebo i dette studiet. Det var spesielt ingen signifikant økning i lever eller muskelenzymer hos Atorvastatin behandlete pasienter.

Tatt i betraktning sikkerhetsdata av rosuvastatin i 4 store prospektive, placebo kontrollerte, multisenter, internasjonale kliniske studier; i Corona studien[19] ble 10 mg rosuvastatin anvendt, i

JUPITER studien[20] 20 mg rosuvastatin, mens både i METEOR [15] og ASTEROIDE[14] studien ble 40 mg rosuvastatin brukt daglig. Det var ingen økning av adverse hendelser eller andre sikkerhets parametere i noen av studiene hos dem som fikk rosuvastatin vs. placebo. Tatt i betraktning tilgjengelig data og klinisk erfaring, er det ikke grunn til å forvente alvorlige bivirkninger eller hendelser ved å behandle RA pasienter med rosuvastatin

I tillegg til at statiner reduserer CRP og virker betennelsesdempende hos pasienter med RA, synes statiner å ha samme lipidsenkende effekt som i den generelle befolkningen. I TARA studien [18], var lipidreduksjonene: Total-Kolesterol (T-Kol): 32 % og LDL: 49 %. Van Doornum[21] har rapportert reduksjon av LDL med 42.4 % på 20 mg Atorvastatin, mens 40 mg Simvastatin[22] reduserte Total-Kolesterol med 21 % og LDL med 33.3 %. I IDEAL studien var reduksjonen av alle lipidene lik etter bruk av 80 mg Atorvastatin eller 20-40 mg Simvastatin hos pasienter som hadde hatt et hjerteinfarkt enten de hadde RA eller ikke (Semb, submitterte data). Dette tiltross for at RA pasientene hadde signifikant lavere total kolesterol og LDL, enn de som ikke hadde RA i utgangspunktet.

Rosuvastatin i vår planlagte studie, vil bli gitt på toppen av annen RA medikasjon, som vil bli registrert. Spesiell forsiktighet vil bli utført når pasientene bruker warfarin eller andre vitamin K antagonister, samt antacida.

Studiens hensikt:

1. Evaluere kolesterolplakk -regresjon og -karakteristika i arteria carotis og en koronararterie etter reduksjon i LDL kolesterol til 1.6-1.8 mmol/L under 18 måneders behandling med Rosuvastatin hos pasienter med Revmatoid Artritt, Behkterew og andre inflammatoriske artritter.
2. Evaluere sammenhengen mellom kolesterolplakk i arteria carotis (med B-mode ultralyd) og koronar arterier, vurdert med CT hos pasienter med Revmatoid Artritt, Bechterew og andre inflammatoriske artritter

Rekruttering prosedyre

1) Pasientene, som alle har fått påvist asymptomatiske kolesterol avleiring i halsen pulsåre ved en tidligere undersøkelse av prosjektleder dr Semb og sonografør Anne Eirheim, ved revmatologisk poliklinikk, vil bli kontaktet med forespørsel om å delta i undersøkelsen og får tilbud om å møte prosjektleder og prosjektsykepleier på revmatologisk poliklinikk for nærmere informasjon. Disse pasientene har også fått utført undersøkelse av sine ledd, sykdoms aktivitet, helsestatus, og undersøkelser av sin arterielle kar stivhet og endotheliale funksjon tidligere. De har også tatt EKG, BT og tidligere besvart "hjerte" spørreskjema med dybde intervju vedr. hjerte-kar symptomer og risikofaktorer. Alle pasientene som bli invitert til å delta i studiet, har gjennomført de prosedyrene beskrevet i screening prosedyren i tidsskjemaet.
2) Ved informasjonsmøtet vil pasientene få muntlig og skriftelig informasjon med mulighet for å undertegne et samtykke om deltagelse. Pasientene vil få med seg en signert kopi av samtykket. Pasientene vil også bli informert om at hvis de ikke ønske å delta i studiet, vil dette

ikke få konsekvenser for dem. Det vil si at de vil ha lik mulighet til undersøkelser, vurdering og oppfølgning av revmatolog og hjertespesialist. Fordelen ved å være med i studiet er at de får en grundigere gjennomgang av din hjerte-kar status enn det som er vanlig ved polikliniske konsultasjoner.

Studiedesign

Deltagerne i studien vil møte på revmatologisk poliklinikk ved Diakonhjemmet Sykehus 1 full dag (eller 2 halve dager) og gjennomgå en full klinisk undersøkelse som inneholder:

Sykdomsaktivitetsmål: Deltagerne svarer på spørreskjemaer og blir undersøkt angående sykdomsaktivitetsmål som brukes i rutineoppfølging av pasienter med revmatiske sykdommer (se appendix 1a og 1 b):

Helsestatus mål: deltagerne svarer på spørreskjemaer (se appendiks 1a og 1 b):

Vi vil dessuten ha et spesielt fokus på ***kartlegging av mulige sykdomskomplikasjoner knyttet til hjerte og blodkar:***

- Spørreskjema over forhold som kan være relatert til forekomst av hjerte-karsykdom hos deg eller din familie
- Blodprøver (ca 20 ml).
- EKG
- Undersøkelse av fingertuppens små blodårer ved at en hylse settes på tuppen av en finger (ITAMAR).
- Trykkmåling og måling av blodstrømmen i håndleddets pulsåre samt i lyske Undersøkelsen utføres ved hjelp av en probe som klemmes lett mot huden.
- Måling av blodstrømmen i armens pulsåre etter 5 minutters avklemming ved hjelp av en blodtrykksmansjett som blir pumpet opp rundt overarmen. (Sphygmacor)
- Ultralydmåling av halsens hovedpulsåre med spesielt fokus på kolesterolavleiring, ved hjelp av en probe som holdes mot halsen.
- Ultralyd og doppler undersøkelse av hjertet.
- CT undersøkelse av hjertet (utføres på Ullevål Universitets sykehus) for å kartlegge kolesterolavleiringer i hjertets pulsårer.

De som får påvist kolesterol avleiring i hjertets kransårer vil også få tilbud om å få utført en

- Venstresidig hjertekateterisering og koronar angiografi (se nedenfor) på Ullevål Universitets sykehus. Undersøkelsen gjøres poliklinisk. Undersøkelsen innbefatter ultralyd av kransarteriene.

Alle pasientene som har undertegnet informert samtykke, vil starte med rosuvastatin 20 mg, 1 tablett pd. etter at de har fått utført alle undersøkelser beskrevet under baseline i tidsforløps-skjemaet. Pasienter over 70 år vil få en startdose av rosuvastatin på 5 mg 1 tablett pd. Dosen av rosuvastatin

opptitreres hver 14. dag til LDL nivåer på 1.6-1.8 mmol/L er nådd. For deltagere under 70 år, vil det bli opptitrert til 40 mg x 1. For deltagere under 70 år vil man opptitrere dosen til 40 mg x 1 dersom LDL nivået over på 1.6-1.8 mmol/L ikke er nådd etter 14 dager. For deltagere med alder over 70 år vil opptitrering være dobling av dosen hver 14. dag inntil LDL på 1.6-1.8 mmol/L er nådd, dvs. 10 mg x 1, 20 mg x 1, 40 mg x 1. Målet er at alle deltagere skal ha nådd 1.6-1.8 mmol/L ved 3 måneder etter start i studien. De pasienter som ikke når mål nivåer av LDL vil ikke bli tatt ut av studiet, men fulgt videre per protokoll. Deltagere vil stå på Rosuvastatin medikasjon i totalt 18 måneder.

Alle resultatene vil avidentifiseres før de analyseres forskningsmessig, men de vil også bli en del av pasientens journal. På den måten vil resultatene være en status som kan brukes for å undersøke om helsetilstanden endrer seg senere.

Inklusjonskriterier:

1. Kvinner og menn med RA, Behkterew og andre inflammatorisk artritter med alder 35-80 år
2. Påvist arteria carotis kolesterolplakk ved ultralyd
3. Informert samtykke

Eksklusjonskriterier:

1. Atrieflimmer eller andre med kronisk uregelmessig hjerterytme (pga. CT)
2. Kontraindikasjon mot statin behandling
    - Hypersensitivitet mot statiner
    - Leversykdom med ASAT/ALAT ≥ 2 ganger øvre normal grense
    - Tidligere statin indusert myopati, eller alvorlig hypersensitivitets reaksjoner mot andre statiner
    - Forhøyet kreatinin (pga. kontrastmidlet)
    - Graviditet eller amming. Gravitest (HCG) vil bli utført hos relevante pasienter og når det er mistanke om graviditet før deltagelse og understudiet.
    - Fertile kvinner som ikke bruke kontraceptiva
    - Cyclosporine behandling
    - Behandling med medikamenter som har kjent interaksjon med rosuvastatin
    - Ukontrollert hypothyroidisme definert som TSH > 1.5 ganger ULN ved første besøk (pga sammenhengen mellom myopati og hypothyroidisme ved statin behandling)
    - Nyresvikt med GFR <60 ml/min/1.73 m^2
3. Sekundær hyperlipidemi
    - Primær hyperthyroidisme

- Nefrotisk syndrom, kreatinin > 2 mg/dl
- Ukontrollert diabetes mellitus (HbA1C > 10 %)
- Plasma Triglyserider > 6.8 mmol/l

4. Andre sykdommer eller behandlinger som reduserer sikkerheten eller behandlingen med rosuvastatin og som vil interferere med endepunktene i studien
    - Hjertesvikt; NYHA klasse III B/IV
    - Hemodynamisk betydningsfulle klaffefeil
    - Etablert statin behandling
    - Gastrointestinal sykdom/behandling som kan gi malabsorbsjon av rosuvastatin
    - Cancer
    - Alvorlig psykiatrisk sykdom
    - Livstruende ventrikulære arrhythmier
    - Annen medikasjon som øker risikoen for rhabdomyolyse
    - Kjent alkohol misbruk
    - Deltagelse i andre studier

Studieprosedyrer

1. Blodprøver:

Det vil bli tatt 20 ml venøst blod på tidspunkter som vist i "flow chart". Følgende parametere vil bli analysert i blodet:

- Creatine kinase, ASAT, ALAT
- Hb, Hct, SR, CRP, hviteblodlegemer, trombocytter, elektrolytter, pBNP, kreatinin, GFR, urinsyre,
- Lipidstatus: total kolesterol, HDL, TG, LDL, Apolipoprotein B, ApolipoproteinA-1, Apolipoprotein(a)
- Blodprøve til analyser av biomarkører/RF/anti-CCP

Det vil også bli utført urin stix undersøkelse

2. Sykdomsaktivitetsmål vedr. ledd sykdom: Deltagerne svarer på spørreskjemaer og blir undersøkt angående sykdomsaktivitetsmål som brukes i rutineoppfølging av pasienter med revmatiske sykdommer (se appendix 1a og 1 b):

a. 28 leddtelling (legges inn av lege eller sykepleier i go treat it (GTI), som er et IT basert rapporterings verktøy.
b. Undersøkers total vurdering av sykdomsaktivitet (legges inn i GTI av den som gjør evalueringen)
c. BASDAI (pasient selvrapportering i GTI)

3. Helsestatus: deltagerne svarer på spørreskjemaer (se appendiks 1a og b):

1. MHAQ (pasient selvrapportering i GTI)
2. BASFI (pasient selvrapportering i GTI)
3. Smerte, tretthet, morgenstivhet, pasients total vurdering (pasient selvrapportering i GTI)
4. undersøkelse av sykdomsaktivitet relatert til din leddsykdom

4. Spørreskjema vedr hjerte-kar symptomer, sykdommer og risikofaktorer
(se appendiks 2)

5. Undersøkelser vedr perifere blodkars funksjon:

a. Undersøkelse av fingertuppens små blodårer ved at en hylse settes på tuppen av en finger (ITAMAR), som er et mål på endothelial funksjon.
b. Trykkmåling og måling av blodstrømmen i håndleddets pulsåre samt i lyske Undersøkelsen utføres ved hjelp av en probe som klemmes lett mot huden.
Måling av blodstrømmen i armens pulsåre etter 5 minutters avklemming ved hjelp av en blodtrykksmansjett som blir pumpet opp rundt overarmen. Det vil bli brukt et Sphygmacor apparat til undersøkelsen

6. B-Mode ultralyd undersøkelse av arteria carotis: Standardisert undersøkelse med registrerte vinkler av hodets dreining i forhold til kroppens midtlinje og probens vinkel på halsen. Intima media tykkelse måles i common carotid artery (CCA) 1 cm ovenfor definert start av bulbus carotis over 1 cm. Det vil også bli tatt bilder av plakk i CCA, bulbus og arteria carotis interna. Man vil vurdere:

1) ***Plakk høyde og areal.*** Karotisplakkets høyde vil bli målt som høyden fra fremre kant av den aterosklerotiske protrusjonen inn i lumen til fremre kant Digital standardised plaque area vurderinger beskrevet av Gray-Weale and Nicolaides,[23] and Nicolaides[24]**,** hvor vi vil bruke AMS program[25]

2) ***Plakk morfologi.***
   a. Plakk klasse: AMS program (Artery Measurement System)
   b. Gray Scale Median value (AMS program)[23-25]

3) ***Intima media tykkelse*** vurdert ved AMS program

7. Transthorakal ekkokardiografi: Det vil gjøres standardiserte opptak og mål ved 2D, M-Mode, og doppler (se appendiks 3).

8. Multi Detector CT angiografi (MDCT) angiografi av hjertet for påvisning av koronare plakk vil bli gjort på Ullevål Universitets sykehus, hvor avd. sjef, professor NE Kløw vil være ansvarlig prosjektleder.

Beskrivelse av CT metoden er vedlagt som Appendix 4

Man vil vurdere

1. Coronary calcium score

2. Koronare plakk
3. Koronar stenoser

Det er 3 mulige diagnoser ved MDCT

1) Normale koronar kar

2) Koronare plakk

3) Koronare stenoser

Analyse av CT funnene blir gjort ved Ullevål Universitetssykehus.

9. Koronar angiografi og intravaskulær ultralyd (IVUS)

(prosedyren er vedlagt som appendix 5). Dette vil bli utført på hjertemedisinsk/radiologisk avdeling Oslo Universitets Sykehus, Ullevål, hvor professor NE Kløw vil være ansvarlig prosjektleder. Analysene av IVUS data vil bli prosessert ved Cleveland Clinic hvor professor S. Nicholls er prosjekt ansvarlig.

Pasienter som ved CT får påvist koronare stenoser eller mistanke om stenoser og pasienter som gjennomgår en CT undersøkelse uten tilfredsstillende kvalitet, vil få tilbud om selektiv koronar angiografi med IVUS. Deres atheroma byrde vil bli vurdert og de vil få tilbud om oppfølgende selektiv koronar angiografi og MDCT etter18 mnd. behandling med rosuvastatin (ved studie slutt)

Pasientene vil ikke få utført en intervensjon under koronar angiografi prosedyren. Pasientene vil derimot bli vurdert kontinuerlig og individuelt med tanke på nødvendigheten av intervensjon. Analyse av IVUS data vil bli utført ved Cleveland Clinic.

<u>Primære endepunkter:</u>

Arteria carotis plakk

Reduksjon av plakk høyde, areal og stabilisering av plakkmorfologi, samt reduksjon av intima media tykkelse etter 18 måneder med rosuvastatin.

<u>Sekundære endepunkter:</u>

Effekten av18 måneders behandling med Rosuvastatin på:

1. **Sykdomsaktivitet og Helsestatus**
    i. **Sykdomsaktivitet** vil bli målt ved: 28-swollen-joint count, AIMS2, AIMS2, BASDAI
    ii. **Helsestatus** vil bli målt ved MHAQ, BASFI, Pain VAS, Fatigue VAS, livskvalitet (HRQoL)
2. **Lipoprotein komponenter**: Lipider, apolipoproteiner, størrelse på disse og funksjonsmål av dem. f eks av HDL
3. **Biomarkører/inflammasjonsparametere**

4. **Kardiovaskulære hendelser** som oppstår under studien definert som; hjerteinfarkt (non ST elevasjon infarkt (NSTEMI) og STEMI, nyoppstått angina pektoris, iskemisk hjerneslag, tranitorsik iskemisk attack (TIA)

<u>Tertiært endepunkter:</u>

Koronar arterie plakk

Endringer i utbredelse og volum av koronare plakk etter 18 mnd med rosuvastatin behandling ved CT og IVUS hos pasienter med koronare plakk ved den initiale undersøkelsen. [13,14]

<u>Legemiddelhåndtering</u>

All studiemedisin vil bli levert av AstraZeneca. Det innebærer produksjon, pakking og forsending. Pasientutlevering og oppbevaring av studiemedisin vil bli utført av sykehusapoteket. Studiemedikamentene vil bli oppbevart i romtemperatur (15-25°C), i tørt område som er avlåst (f eks i avlåst skap eller medikament rom, beskyttet fra utilsiktet bruk.

<u>Compliance</u>

Pasienten vil få utlevert nødvendig antall tabletter fra apoteket ved hvert besøk. Påfølgende besøk leveres returmedisin til utprøver. Ut ifra antall returnerte tabletter beregnes compliance i prosent. Kravet for å inkludere pasienten i per-protokolgruppen er over 80 % compliance.

<u>Datainnsamling</u>

All datainnsamling vil bli registrert i case report forms (CRF), som skrives uavhengig av vanlig journalføring i henhold til GCP. Kvalitetskontroll av innsamplet data vil bli utført internt på Revmatologisk avdeling av studiepersonell.

<u>Rapportering av bivirkninger</u>

Registrering av bivirkninger (adverse events; AE) vil bli gjort ved hvert besøk, i tillegg til at pasienten blir oppfordret til å ta kontakt direkte med prosjektansvarlige eller studiesykepleier og rapportere dersom en bivirkning skulle oppstå. Man vil intervjue pasientene om bivirkninger med spørsmål som: "Har du hatt noen helse problemer siden sist besøk (visit)?" Data vil bli dokumentert i CRF og rapportert i henhold til retningslinjene. Utprøver vil melde SLV (med kopi til AstraZeneca) så rask som mulig, men innen 7 dager ved mistenkte uventede alvorlige bivirkninger (SUSAR) og innen 15 dager ved mistenkte uventende bivirkninger etter at utprøver har fått kjennskap til hendelsen.

Interim analyse vil bli foretatt

1. Når 30 pasienter har entret studien og fått utført CT cor og evt selektiv coronar angiografi. Man vil da vurdere antall pasienter som sannsynligvis skal videre til selektiv coronar angiografi.
2. Halvveis i studien dvs. i midten av juni 2011vil man vurdere safety parametere beskrevet under avsnittet: Avslutning fra deltagelse i studiet

Studie størrelse, statistiske analyser og studiets varighet

Man kan forvente 4.2 % reduksjon av plakkareale (arteria carotis) sammenliknet med baseline etter behandling med rosuvastatin i 2.5 år sammenliknet med en placebo gruppe.[26] Flere intravaskulære ultralyd studier har vist at reduksjon av LDL med statiner forsinker progresjonen av atherosklerose og kan indusere regresjon[13] etter 18 måneder med statin behandling dersom LDL nivået reduseres til 1.6-1.8 mmol/L.

Pasienter med kolesterol plakk i arteria carotis, har vaskulær atherosklerotisk sykdom og skal etter norske retningslinjer og internasjonale guidelines, både de Europeiske og Amerikanske har sekundær profylaktisk forebyggende behandlinger hvor mål nivået for LDL er 1.8 mmol/L. Med bakgrunn i dette, kan det ikke gjennomføres en placebo gruppe i dette studiet.

Studien trenger 75 pasienter for å detektere en endring på 4.2 % areal med SD= 13.0 % med 80 % sikkerhet på 5 % signifikansnivå. Realismen av standardisert effekt størrelse på 3.1 virker rimelig ut ifra resultater fra ASTEROID studien som fant netto forskjell mellom behandlings og placebo gruppe i SD enheter lik 3.25. Det vil da være 80 % styrke for å detektere en 4.2 % reduksjon av plakk arealet i arteria carotis hos 100 pasienter, hvis standard deviasjonen (SD) av prosent forandring fra start til slutt av studiet (18 mnd. behandling) er mindre enn 3.1 ganger prosent gjennomsnittelig reduksjon. Man vil legge utføre en interim sjekk av SD etter halvgått løp for å se om forutsetningen (Ratio SD/effekt=3.1) holder. Missing data vil bli håndtert på følgende måte: Pasienter som ikke følger protokollen fullstendig, vil inngå i intention-to-treat analysegruppen (ITT). Eksepler på tilfeller der det vil være aktuelt er compliance under 90 %, protokoll avvik, ikke møtt til studiebesøk osv. Hvis pasienter dropper ut av studien vil last observation carried forward (LOCF) teknikken brukes i intension-to-treat analysen av disse pasientene. Pasienter som følger protokollen fullstendig vil inngå i per-protokoll analysen. De pasienter som avslutter sin deltagelse i studien før planlagt, vil bli fulgt opp med et telefon intervju på tidspunktet for slutt av studien for denne pasienten.

Ett hundre pasienter vil bli rekruttert fortløpende fra pågående studier og vil bli fulgt prospektivt, i en åpen, blindet evaluering av endepunktene studie design, hvor plakk evalueringen i både arteria carotis og koronar arteriene vil bli utført blindet i forhold til pasient identifikasjon og undersøkelses-sekvens.

Man vil bruke:

- Parete undersøkelser for å sammenlikne forandringer i plakkene, lipider og sekundære endepunkter.
- Bivariable undersøkelser vedr assosiasjoner mellom forandringer i plakk og sekundære variabler.
- Longitudinelle analyser (demografiske variabler i "generalized estimating equations (GEE) for å undersøke longitudinelle uavhengige assosiasjoner mellom plakk og sekundære endepunkter.
- Skjevfordelte variabler vil bli undersøkt etter logaritmisk transformasjon.

- Man vil bruke "intention to treat" analyse for pasienter som ikke følger protokollen fullstendig og last observation carried forward- metoden ved beregning av endepunkt.

Etiske vurderinger

Studien vil bli gjennomført i henhold til Helsinkideklarasjonen (WMA, 1964). Studien er godkjent av regionalt etisk komité helse Sørøst (REK), har godkjennelse fra SLV, personvern, datatilsynet og har godkjennelse for biobank. Studien er meldt clinicaltrials.gov. Pasientene vil få skriftlig informasjon om dette i informasjonsbrev før deltagelse i prosjektet.

Pasientpopulasjonen som undersøkes er i utgangspunktet en høyrisiko populasjon med 2-3 ganger høyere risiko for en KV hendelse sammenliknet med den generelle befolkningen. Det vil derfor være klinisk indikasjon for invasive hjerte-kar undersøkelse av disse pasientene. Det finnes ikke kunnskap om sammenhengen mellom arteria carotis plakk og koronare plakk hos pasienter med inflammatorisk leddsykdom. Først vil alle deltagere i studiet få tilbud om CT koronarangiografi. CT undersøkelsen er rapportert å ha høy diagnostisk sikkerhet av koronare stenoser på 50 % hos pasienter med brystsmerter men ikke kjent hjerte-kar sykdom.[27;28] Pasienter som får påvist koronare plakk og stenoser henvises videre til koronar angiografi som inkluderer intravaskulær ultralyd (IVUS) undersøkelse. Ved funn av signifikante stenoser, vil de gjeldende retningslinjer for behandling, vedrørende utblokking av stenosen (PCI) eller bypass kirurgi (CABG) bli fulgt.

Risiko ved CT undersøkelsen er knyttet til bruk av kontrastmiddel og bruk av røntgenstråler. Faren ved kontrastmiddel er minimal hos pasienter med normal nyrefunksjon. Alvorlige allergiske reaksjoner forekommer hos færre enn 1:10.000. Den teoretiske risiko ved strålebelastningen er særlig et anliggende der yngre pasienter undersøkes. Metoden er i utstrakt klinisk bruk.

Risiko ved koronar angiografi og IVUS er særlig knyttet til alvorligheten av pasientens karsykdom og alder. I Asteroide studien[14] som undersøkte 507 pasienter med koronar angiografi og IVUS før og etter 18 måneders behandling med 40 mg rosuvastatin, var det fire pasienter som døde (0.8 %), 10 som fikk hjerteinfarkt (2.0 %), og tre pasienter som fikk slag (0.6 %). Ut fra dette vil man kunne forvente i aktuelle studie med 100 deltagere at det vil oppstå 0.8 dødsfall, 2 hjerteinfarkter og 0.6 slag. Totale komplikasjoner hos aktuelle pasientgruppe ved UUS er godt under 1 %, de fleste er hematomer på stikkstedet. Dødsrisiko ligger på < 1/1000 og faren for slag ca 1/1000.

Avslutning av deltagelse i studiet

1. Tilbaketrekking av informert samtykke.
2. Ethvert tidspunkt hvor CK måles >5 x ULN (øvre normal grense) og/eller er ledsaget av muskel -ubehag, -smerter, -sårhet og/eller -svakhet som er alvorlige selv om CK måles <5 x ULN
3. Persisterende ASAT/ALAT >3 x ULN ved 2 målinger med minst 48 timer mellom.

4. Dersom kreatinin stiger og GFR faller til <60 ml/min/1.73 m^2 ved 2 målinger med minst 48 timer mellom når pasienten bruker rosuvastatin i studien, vil pasienten bli tatt ut av studien.
5. Hvis pasientens helsetilstand blir dårligere, vurdert av utprøver, som krever at studie medikasjonen seponeres.
6. Hvis en bivirkning oppstår, hvor det ifølge utprøver er nødvendig å seponere studie-medikasjonen.
7. Graviditet
8. Dersom utprøver finner det nødvendig.

Forsikring

Alle pasientene vil bli forsikret via Legemiddelansvarforsikringen.

Etikk og konsesjon fra Datatilsynet og Biobank

Studien er anbefalt av Personvernombudet (03.09.09) og SLV (09.10.09). Det sendes søknad til REK med søknad om Biobank 09.11.09.

Fremdrift – milepæler - ansvarsforhold:

Dette er en utprøver initiert studie, hvor utprøverene har alle rettigheter til data materialet og publiserings rettigheter av disse.

 Januar 2010<br>Desember 2010<br>Juni 2012	Datainnsamling:<br>Første pasient inn<br>Siste pasient inn<br>Siste pasientbesøk
2012	Databearbeiding, analyse, og publisering av materialet

(1) Del Rincon I, Williams K, Stern MP, Freeman GL, Escalante A. High incidence of cardiovascular events in a rheumatoid arthritis cohort not explained by traditional cardiac risk factors. Arthritis Rheum 2001; 44(12):2737-2745.

(2) Maradit-Kremers H, Nicola PJ, Crowson CS, Ballman KV, Gabriel SE. Cardiovascular death in rheumatoid arthritis: a population-based study. Arthritis Rheum 2005; 52(3):722-732.

(3) Maradit-Kremers H, Crowson CS, Nicola PJ, Ballman KV, Roger VL, Jacobsen SJ et al. Increased unrecognized coronary heart disease and sudden deaths in rheumatoid arthritis: a population-based cohort study. Arthritis Rheum 2005; 52(2):402-411.

(4) van Halm V, Peters MJ, Voskuyl AE, Boers M, Lems WF, Visser M et al. Rheumatoid arthritis versus diabetes as a risk factor for cardiovascular disease, a cross sectional study. The CARRE Investigation. Ann Rheum Dis 2008.

(5) Dessein PH, Joffe BI, Veller MG, Stevens BA, Tobias M, Reddi K et al. Traditional and nontraditional cardiovascular risk factors are associated with atherosclerosis in rheumatoid arthritis. J Rheumatol 2005; 32(3):435-442.

(6) Simons PC, Algra A, Bots ML, Grobbee DE, van der GY. Common carotid intima-media thickness and arterial stiffness: indicators of cardiovascular risk in high-risk patients. The SMART Study (Second Manifestations of ARTerial disease). Circulation 1999; 100(9):951-957.

(7) Pignoli P, Tremoli E, Poli A, Oreste P, Paoletti R. Intimal plus medial thickness of the arterial wall: a direct measurement with ultrasound imaging. Circulation 1986; 74(6):1399-1406.

(8) Gonzalez-Juanatey C, Llorca J, Martin J, Gonzalez-Gay MA. Carotid Intima-Media Thickness Predicts the Development of Cardiovascular Events in Patients with Rheumatoid Arthritis. Semin Arthritis Rheum 2008.

(9) Roman MJ. Preclinical carotid atherosclerosis in patients with rheumatoid arthritis. . Moeller E, Davis A, Paget SA, Crow MK, Lockshin MD, editors. Ann Intern Med 144, 249-256. 2006.

(10) Riccioni G, Bazzano LA, Bucciarelli T, Mancini B, di IE, D'Orazio N. Rosuvastatin reduces intima-media thickness in hypercholesterolemic subjects with asymptomatic carotid artery disease: the Asymptomatic Carotid Atherosclerotic Disease in Manfredonia (ACADIM) Study. Expert Opin Pharmacother 2008; 9(14):2403-2408.

(11) Pedersen TR, Faergeman O, Kastelein JJ, Olsson AG, Tikkanen MJ, Holme I et al. High-dose atorvastatin vs usual-dose simvastatin for secondary prevention after myocardial infarction: the IDEAL study: a randomized controlled trial. JAMA 2005; 294(19):2437-2445.

(12) LaRosa JC, Grundy SM, Waters DD, Shear C, Barter P, Fruchart JC et al. Intensive lipid lowering with atorvastatin in patients with stable coronary disease. N Engl J Med 2005; 352(14):1425-1435.

(13) Nicholls SJ, Tuzcu EM, Sipahi I, Grasso AW, Schoenhagen P, Hu T et al. Statins, high-density lipoprotein cholesterol, and regression of coronary atherosclerosis. JAMA 2007; 297(5):499-508.

(14) Nissen SE, Nicholls SJ, Sipahi I, Libby P, Raichlen JS, Ballantyne CM et al. Effect of very high-intensity statin therapy on regression of coronary atherosclerosis: the ASTEROID trial. JAMA 2006; 295(13):1556-1565.

(15) Crouse JR, III, Raichlen JS, Riley WA, Evans GW, Palmer MK, O'Leary DH et al. Effect of rosuvastatin on progression of carotid intima-media thickness in low-risk individuals with subclinical atherosclerosis: the METEOR Trial. JAMA 2007; 297(12):1344-1353.

(16) Nezasa K, Higaki K, Takeuchi M, Nakano M, Koike M. Uptake of rosuvastatin by isolated rat hepatocytes: comparison with pravastatin. Xenobiotica 2003; 33(4):379-388.

(17) Marzolini C, Tirona RG, Kim RB. Pharmacogenomics of the OATP and OAT families. Pharmacogenomics 2004; 5(3):273-282.

(18) McCarey DW, McInnes IB, Madhok R, Hampson R, Scherbakov O, Ford I et al. Trial of Atorvastatin in Rheumatoid Arthritis (TARA): double-blind, randomised placebo-controlled trial. Lancet 2004; 363(9426):2015-2021.

(19) Kjekshus J, Apetrei E, Barrios V, Bohm M, Cleland JG, Cornel JH et al. Rosuvastatin in older patients with systolic heart failure. N Engl J Med 2007; 357(22):2248-2261.

(20) Ridker PM, Danielson E, Fonseca FA, Genest J, Gotto AM, Jr., Kastelein JJ et al. Rosuvastatin to Prevent Vascular Events in Men and Women with Elevated C-Reactive Protein. N Engl J Med 2008.

(21) Van Doornum S., McColl G, Wicks IP. Atorvastatin reduces arterial stiffness in patients with rheumatoid arthritis. Ann Rheum Dis 2004; 63(12):1571-1575.

(22) Hermann F, Forster A, Chenevard R, Enseleit F, Hurlimann D, Corti R et al. Simvastatin improves endothelial function in patients with rheumatoid arthritis. J Am Coll Cardiol 2005; 45(3):461-464.

(23) Gray-Weale AC, Graham JC, Burnett JR, Byrne K, Lusby RJ. Carotid artery atheroma: comparison of preoperative B-mode ultrasound appearance with carotid endarterectomy specimen pathology. J Cardiovasc Surg (Torino) 1988; 29(6):676-681.

(24) Nicolaides AN, Shifrin EG, Bradbury A, Dhanjil S, Griffin M, Belcaro G et al. Angiographic and duplex grading of internal carotid stenosis: can we overcome the confusion? J Endovasc Surg 1996; 3(2):158-165.

(25) Geroulakos G, Ramaswami G, Nicolaides A, James K, Labropoulos N, Belcaro G et al. Characterization of symptomatic and asymptomatic carotid plaques using high-resolution real-time ultrasonography. Br J Surg 1993; 80(10):1274-1277.

(26) Spence JD, Eliasziw M, DiCicco M, Hackam DG, Galil R, Lohmann T. Carotid plaque area: a tool for targeting and evaluating vascular preventive therapy. Stroke 2002; 33(12):2916-2922.

(27) Budoff MJ, Dowe D, Jollis JG, Gitter M, Sutherland J, Halamert E et al. Diagnostic performance of 64-multidetector row coronary computed tomographic angiography for evaluation of coronary artery stenosis in individuals without known coronary artery disease: results from the prospective multicenter ACCURACY (Assessment by Coronary Computed Tomographic Angiography of Individuals Undergoing Invasive Coronary Angiography) trial. J Am Coll Cardiol 2008; 52(21):1724-1732.

(28) Ostrom MP, Gopal A, Ahmadi N, Nasir K, Yang E, Kakadiaris I et al. Mortality incidence and the severity of coronary atherosclerosis assessed by computed tomography angiography. J Am Coll Cardiol 2008; 52(16):1335-1343.

RORA Appendix 1 a

Navn: ________________________________ Id.nr. _____ dato: ________

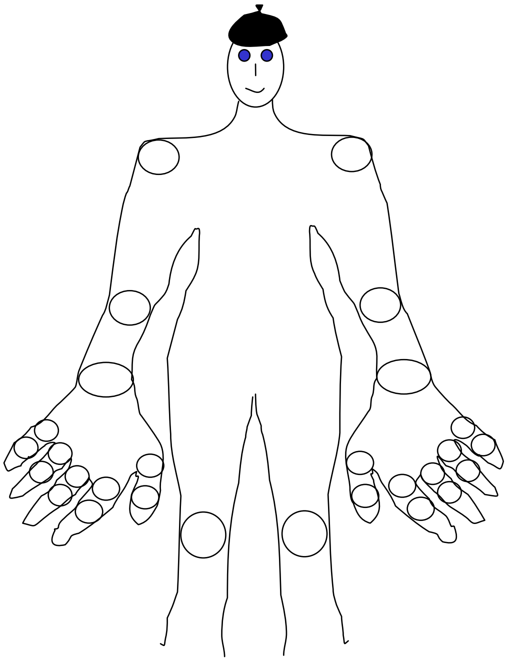

Hovne

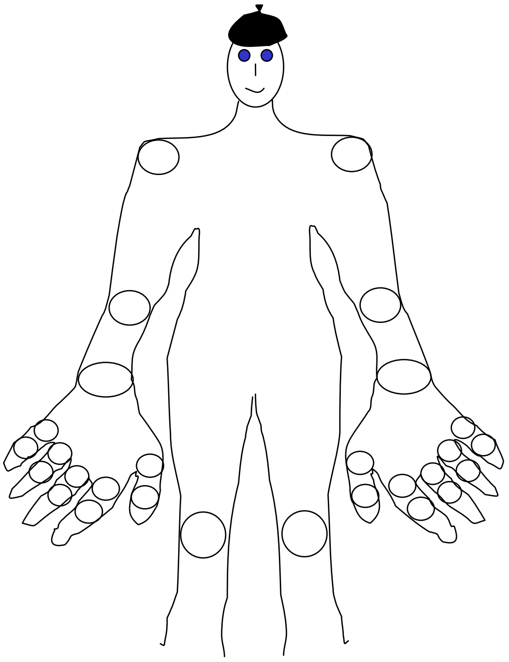

Ømme

Antall hovne ledd (0 - 28) **|__|__|**
Antall ømme ledd (0 - 28) **|__|__|**

Antall ikke evaluerbare ledd (for eksempel pga. proteser) **|__|__|**

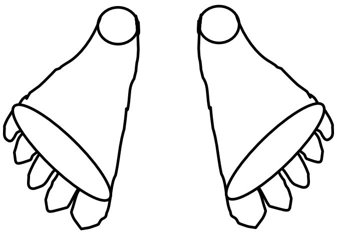

Hovne

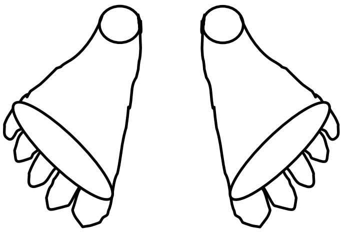

Ømme

**Antall hovne fotledd (0 - 4)** **|__|__|**	**Antall ømme fotledd (0 – 4)** **|__|__|**

Totalvurdering av sykdomsaktivitet (sett strek på tvers av linjen)
Sett strek på tvers av linjen. (Fylles ut av lege/sykepleier).

|---|
Lav Høy

Vekt ☐☐☐ kg Høyde ☐☐☐ cm Livvidde ☐☐☐ cm

Alder ved menopause:

Blodtrykk systolisk	☐☐☐	☐☐☐
Blodtrykk diastolisk	☐☐☐	☐☐☐
Puls	☐☐☐	☐☐☐

						**Seponeringsgrunn**		
**Legemiddel**	Bruker nå	**Aldri brukt**	**Brukt tidl**	**Fra**	**Til**	**Mang-lende effekt**	**Bivirkninger**	**Ukjent/ annet**
Auranofin	1 ☐	1 ☐	2 ☐			1 ☐	2 ☐	3 ☐
Adalimumab	1 ☐	1 ☐	2 ☐			1 ☐	2 ☐	3 ☐
Anakinra	1 ☐	1 ☐	2 ☐			1 ☐	2 ☐	3 ☐
Azatioprin	1 ☐	1 ☐	2 ☐			1 ☐	2 ☐	3 ☐
Cyclosporin	1 ☐	1 ☐	2 ☐			1 ☐	2 ☐	3 ☐
D-penicillamin	1 ☐	1 ☐	2 ☐			1 ☐	2 ☐	3 ☐
Etanercept	1 ☐	1 ☐	2 ☐			1 ☐	2 ☐	3 ☐
Gull-tiomalat	1 ☐	1 ☐	2 ☐			1 ☐	2 ☐	3 ☐
Hydroxyklorokin/klorokin	1 ☐	1 ☐	2 ☐			1 ☐	2 ☐	3 ☐
Infliximab	1 ☐	1 ☐	2 ☐			1 ☐	2 ☐	3 ☐
Leflunomid	1 ☐	1 ☐	2 ☐			1 ☐	2 ☐	3 ☐
Methotrexat	1 ☐	1 ☐	2 ☐			1 ☐	2 ☐	3 ☐
Sulfasalazin	1 ☐	1 ☐	2 ☐			1 ☐	2 ☐	3 ☐
Reumacon	1 ☐	1 ☐	2 ☐			1 ☐	2 ☐	3 ☐
Rituximab	1 ☐	1 ☐	2 ☐			1 ☐	2 ☐	3 ☐
MTX+antimalaria	1 ☐	1 ☐	2 ☐			1 ☐	2 ☐	3 ☐
MTX+antimalaria+sulfasalazin	1 ☐	1 ☐	2 ☐			1 ☐	2 ☐	3 ☐
MTX+sulfasalazin	1 ☐	1 ☐	2 ☐			1 ☐	2 ☐	3 ☐
MTX+cyklosporin	1 ☐	1 ☐	2 ☐			1 ☐	2 ☐	3 ☐
MTX+etanercept	1 ☐	1 ☐	2 ☐			1 ☐	2 ☐	3 ☐
MTX+infliximab	1 ☐	1 ☐	2 ☐			1 ☐	2 ☐	3 ☐
MTX+Adalumimab	1 ☐	1 ☐	2 ☐			1 ☐	2 ☐	3 ☐
MTX+Anakinra	1 ☐	1 ☐	2 ☐			1 ☐	2 ☐	3 ☐
Andre (spesifiser):	1 ☐	1 ☐	2 ☐			1 ☐	2 ☐	3 ☐
Andre (spesifiser):	1 ☐	1 ☐	2 ☐			1 ☐	2 ☐	3 ☐

Andre (spesifiser):	1 ☐	1 ☐	2 ☐			1 ☐	2 ☐	3 ☐
	1 ☐	1 ☐	2 ☐			1 ☐	2 ☐	3 ☐
	1 ☐	1 ☐	2 ☐			1 ☐	2 ☐	3 ☐

EKSTRAARTIKULÆRE KOMPLIKASJONER (nå eller tidligere)

**Komplikasjon**	**Ikke tilstede**	**Mulig tilstede**	**Sikkert tilstede**	**Kommentar**
Vaskulitt indre organer	1 ☐	2 ☐	3 ☐	
Kutan vaskulitt	1 ☐	2 ☐	3 ☐	
Lungefibrose	1 ☐	2 ☐	3 ☐	
Pleuritt	1 ☐	2 ☐	3 ☐	
Perikarditt	1 ☐	2 ☐	3 ☐	
Skleritt	1 ☐	2 ☐	3 ☐	
Polyneuropati/mononeuropati	1 ☐	2 ☐	3 ☐	
Sekundært Sjøgrens syndrom	1 ☐	2 ☐	3 ☐	
Sekundær amyloidose	1 ☐	2 ☐	3 ☐	
Felty syndrom	1 ☐	2 ☐	3 ☐	
Annet	1 ☐	2 ☐	3 ☐	

Diakonhjemmet Sykehus

SPØRSMÅL OM FUNKSJON, SMERTE, TRETTHET, SYKDOMSAKTIVITET OG ANDRE PLAGER (MHAQ)

**I LØPET AV SISTE UKEN, KUNNE DU:**	UTEN problemer	med VISSE problemer	med STORE problemer	kunne IKKE
Kle på deg selv, inkl. å knytte skolisser og å kneppe knapper?	☐	☐	☐	☐
Komme opp i og ut av sengen?	☐	☐	☐	☐
Løfte en full kopp eller et fullt glass til munnen?	☐	☐	☐	☐
Gå utendørs på flat mark?	☐	☐	☐	☐
Vaske og tørke deg over hele kroppen?	☐	☐	☐	☐
Bøye deg for å ta opp klær fra gulvet?	☐	☐	☐	☐
Skru vanlige kraner opp og igjen?	☐	☐	☐	☐
Komme inn og ut av en bil?	☐	☐	☐	☐

Hvordan vil du beskrive de leddsmertene du vanligvis har hatt den siste uken?

Sett strek på tvers av linjen.

|---|

ingen smerter — uutholdelige smerter

Har du hatt problemer med følelse av tretthet den siste uken?

Sett strek på tvers av linjen.

|---|

Tretthet er ikke noe problem — Tretthet er et stort problem

Spørsmål om sykdomsaktivitet

Vi ber deg vennligst vurdere aktiviteten i leddgiktsykdommen i løpet av den siste uken.

Når du tar alle symptomer med i betraktning, hvordan synes du tilstanden er?

|--|

Bra, ingen symptomer — Svært dårlig

Hvordan vurderer du din nåværende tilstand tatt i betraktning ditt generelle funksjonsnivå og de smerter du har nå?

Svært god	God	Tilfresstillende	Dårlig	Svært dårlig
1 ☐	2 ☐	3 ☐	4 ☐	5 ☐

MÅLESKJEMA FOR VIRKNINGER AV GIKTSYKDOM (AIMS)

Rettledning:

Vi ber deg vennligst svare på de følgende spørsmålene om helsen din. De fleste spørsmålene gjelder helsetilstanden **i løpet av den siste måneden**. Alle svarene dine er viktige. De fleste spørsmålene kan besvares ved å sette et kryss. Det er viktig at du svarer på alle spørsmålene.

Sett kryss ved det svaret som passer best til hvert enkelt spørsmål.

Disse spørsmålene dreier seg om **RØRLIGHET**

I LØPET AV DEN SISTE MÅNEDEN...	hver dag	de fleste dager	noen dager	noen få dager	ingen dager
Hvor ofte var du i fysisk stand til å kjøre bil eller bruke offentlige transportmidler?	☐	☐	☐	☐	☐
Hvor ofte var du ute av huset, i hvert fall en del av dagen?	☐	☐	☐	☐	☐
Hvor ofte var du i stand til å gjøre ærender i området der du bor?	☐	☐	☐	☐	☐
Hvor ofte trengte du hjelp med å bevege deg utenfor hjemmet?	☐	☐	☐	☐	☐
Hvor ofte var du i seng eller satt i en stol hele eller det meste av dagen?	☐	☐	☐	☐	☐

Disse spørsmålene dreier seg om **RØRLIGHET I HENDER OG FINGRE**

I LØPET AV DEN SISTE MÅNEDEN...	hver dag	de fleste dager	noen dager	noen få dager	ingen dager
Kunne du lett skrive med penn eller blyant?	☐	☐	☐	☐	☐
Kunne du lett kneppe en skjorte eller en bluse?	☐	☐	☐	☐	☐
Kunne du lett vri om nøkkelen i en lås?	☐	☐	☐	☐	☐
Kunne du lett knytte en knute eller lage en sløyfe?	☐	☐	☐	☐	☐
Kunne du lett åpne et nytt glass med skrulokk?	☐	☐	☐	☐	☐

Disse spørsmålene dreier seg om **KONTAKT MED ANDRE MENNESKER**

I LØPET AV DEN SISTE MÅNEDEN...	hver dag	de fleste dager	noen dager	noen få dager	ingen dager
Hvor ofte var du sammen med venner eller slektninger?	☐	☐	☐	☐	☐
Hvor ofte hadde du venner eller slektninger på besøk?	☐	☐	☐	☐	☐
Hvor ofte besøkte du venner eller slektninger?	☐	☐	☐	☐	☐
Hvor ofte snakket du i telefonen med nære venner eller slektninger?	☐	☐	☐	☐	☐
Hvor ofte var du på møte i en menighet, lag, klubb, gruppe eller en annen forsamling?	☐	☐	☐	☐	☐

Disse spørsmålene dreier seg om **STØTTE FRA FAMILIE OG VENNER**

I LØPET AV DEN SISTE MÅNEDEN...	hver dag	de fleste dager	noen dager	noen få dager	ingen dager
Følte du at familie eller venner ville hjulpet deg hvis du trengte hjelp?	☐	☐	☐	☐	☐
Følte du at familie eller venner var oppmerksomme på dine personlige behov?	☐	☐	☐	☐	☐
Følte du at familie eller venner var interessert i å hjelpe deg med dine problemer?	☐	☐	☐	☐	☐
Følte du at familie eller venner forsto hvordan giktsykdommen virket på deg?	☐	☐	☐	☐	☐

Disse spørsmålene dreier seg om **REVMATISKE SMERTER**

I LØPET AV DEN SISTE MÅNEDEN...	sterke	moderate	milde	svært milde	ingen
Hvordan vil du beskrive de revmatiske smertene du vanligvis hadde?	☐	☐	☐	☐	☐

	hver dag	de fleste dager	noen dager	noen få dager	ingen dager

I LØPET AV DEN SISTE MÅNEDEN...					
Hvor ofte hadde du sterke revmatiske smerter?	☐	☐	☐	☐	☐
Hvor ofte hadde du smerter i to eller flere ledd samtidig?	☐	☐	☐	☐	☐
Hvor ofte varte morgenstivheten lenger enn en time etter at du våknet?	☐	☐	☐	☐	☐
Hvor ofte gjorde smertene det vanskelig for deg å sove?	☐	☐	☐	☐	☐

Disse spørsmålene dreier seg om ARBEID

I LØPET AV DEN SISTE MÅNEDEN...	betalt arbeid	hus-arbeid	skole-arbeid	arbeids-ledig	trygdet	pensjo-nist
Hva har vært ditt viktigste arbeid?	☐	☐	☐	☐	☐	☐

Hvis du svarer arbeidsledig, trygdet eller pensjonist, kan du hoppe over de neste fire spørsmålene og gå til spørsmålene om anspenthet.

I LØPET AV DEN SISTE MÅNEDEN...	hver dag	de fleste dager	noen dager	noen få dager	ingen dager
Hvor ofte klarte du ikke å gjøre noen form for betalt arbeid, husarbeid eller skolearbeid?	☐	☐	☐	☐	☐
Hvor ofte måtte du redusere arbeidstiden de dagene du arbeidet?	☐	☐	☐	☐	☐
Hvor ofte klarte du ikke til å gjøre arbeidet så grundig og så nøye som du selv ville de dagene du arbeidet?	☐	☐	☐	☐	☐
Hvor ofte måtte du endre arbeidsmåten de dagene du arbeidet?	☐	☐	☐	☐	☐

Disse spørsmålene dreier seg om ANSPENTHET

I LØPET AV DEN SISTE MÅNEDEN...	alltid	svært ofte	noen ganger	nesten aldri	aldri

Hvor ofte følte du deg anspent eller amper?	☐	☐	☐	☐	☐
Hvor ofte ble du plaget med nervøsitet eller irritabilitet?	☐	☐	☐	☐	☐
Hvor ofte greide du å slappe av uten vanskelighet?	☐	☐	☐	☐	☐
Hvor ofte følte du deg avslappet og uanspent?	☐	☐	☐	☐	☐
Hvor ofte følte du deg rolig og avbalansert?	☐	☐	☐	☐	☐

Disse spørsmålene dreier seg om SINNSSTEMNING

I LØPET AV DEN SISTE MÅNEDEN...	alltid	svært ofte	noen ganger	nesten aldri	aldri
Hvor ofte gledet du deg over det du holdt på med?	☐	☐	☐	☐	☐
Hvor ofte var du nedtrykt eller langt nede?	☐	☐	☐	☐	☐
Hvor ofte følte du at ingen ting ble slik du ønsket?	☐	☐	☐	☐	☐
Hvor ofte følte du at andre ville hatt det bedre hvis du var død?	☐	☐	☐	☐	☐
Hvor ofte følte du deg så nedfor at ingenting kunne hjelpe på humøret?	☐	☐	☐	☐	☐

Spørsmålene dreier seg om NÅVÆRENDE og FRAMTIDIG HELSE

	utmerket	god	nokså bra	dårlig
Vil du generelt si at DIN HELSE NÅ er utmerket, god, nokså bra eller dårlig? ☐	☐	☐		☐

	svært fornøyd	verken fornøyd eller misfornøyd	litt misfornøyd	misfornøyd	svært misfornøyd
Hvor fornøyd er du med DIN HELSE NÅ?	☐	☐	☐	☐	☐

Dette spørsmålet dreier seg om den TOTALE VIRKNINGEN AV GIKTSYKDOMMEN

	svært bra	bra	tilfreds-stillende	dårlig	svært dårlig
Hvor godt klarer du deg sammenlignet med andre mennesker på din alder, når du tar hensyn til alle de områdene giktsykdommen virker inn på?	☐	☐	☐	☐	☐

Diakonhjemmet Sykehus

SPØRRESKJEMA OM LIVSKVALITET

Vennligst les gjennom alle svaralternativene til hvert spørsmål før du plasserer et kryss (x) for det alternativet som best beskriver din nåværende tilstand. Fortsett på samme måte for alle 14 spørsmålene. Gi bare ett svar på hvert spørsmål.

SPØRSMÅL 1. **BEVEGELIGHET**

1☐ Jeg er i stand til å gå normalt (uten vanskelighet) innendørs, utendørs og i trapper
2☐ Jeg er i stand til å gå uten vanskelighet innendørs, men utendørs og/eller i trapper har jeg litt problemer
3☐ Jeg er i stand til å gå uten hjelp innendørs (med eller uten et hjelpemiddel), men utendørs og/eller i trapper bare med betydelig vanskelighet eller med hjelp fra andre.
4☐ Jeg er i stand til å gå innendørs kun med hjelp fra andre.
5☐ Jeg er fullstendig sengeliggende og ute av stand til å bevege meg omkring.

SPØRSMÅL 2. **SYN**

1☐ Jeg ser normalt, dvs. jeg kan lese aviser og tekst på TV uten vanskelighet (med eller uten briller).
2☐ Jeg kan lese aviser og/eller tekst på TV med litt vansker (med eller uten briller).
3☐ Jeg kan lese aviser og/eller tekst på TV med betydelige vansker (med eller uten briller).
4☐ Jeg kan ikke lese aviser eller tekst på TV hverken med briller eller uten, men jeg kan se godt nok til å gå omkring uten hjelp.
5☐ Jeg kan ikke se godt nok til å gå omkring uten en hjelper, dvs. jeg er nesten eller helt blind.

SPØRSMÅL 3. **HØRSEL**

1☐ Jeg hører normalt, dvs. normal tale (med eller uten et høreapparat).
2☐ Jeg hører normal tale med litt vansker.
3☐ Jeg hører normal tale med betydelige vansker; i samtaler må stemmer være høyere enn normalt.
4☐ Jeg hører selv sterke stemmer dårlig; jeg er nesten døv.
5☐ Jeg er helt døv.

SPØRSMÅL 4. **PUST**

1☐ Jeg er i stand til å puste normalt, dvs. uten å være kortpustet eller ha andre pustevansker.
2☐ Jeg er kortpustet under tungt arbeid eller sport, eller når jeg går raskt på flat mark eller i slak motbakke.
3☐ Jeg er kortpustet når jeg går på flat mark med samme tempo som andre på min alder.
4☐ Jeg blir kortpustet selv etter lett aktivitet, f.eks. når jeg vasker meg eller kler på meg.
5☐ Jeg har pustevansker nesten hele tiden, selv i hvile.

SPØRSMÅL 5. **SØVN**

1☐ Jeg er i stand til å sove normalt, dvs. jeg har ingen problemer med å sove.
2☐ Jeg har lette søvnproblemer, f.eks. vanskelig for å falle i søvn eller våkner av og til om natte
3☐ Jeg har moderate søvnproblemer, f.eks. forstyrret søvn eller føler jeg ikke har sovet nok.
4☐ Jeg har store søvnproblemer, f.eks. må bruke sovemedisiner ofte eller rutinemessig, eller våkner om natten og/eller for tidlig om morgenen.
5☐ Jeg lider av alvorlig søvnløshet, f.eks. er søvn nesten umulig selv med bruk av sovemedisiner, eller jeg forblir våken det meste av natten.

SPØRSMÅL 6. **SPISING**

1☐ Jeg er i stand til å spise normalt, dvs. uten hjelp fra andre.
2☐ Jeg er i stand til å spise selv med mindre vansker (f.eks. langsomt, klønete, skjelvende, 3 eller med spesielle hjelpemidler).
3☐ Jeg trenger noe hjelp fra en annen person for å spise.
4☐ Jeg er ute av stand til å spise selv i det hele tatt, slik at jeg må mates av en annen person.
5☐ Jeg er ute av stand til å spise i det hele tatt, slik at jeg mates enten med slange eller intravenøst.

SPØRSMÅL 7. **TALE**

1☐ Jeg er i stand til å snakke normalt, dvs. klart, hørbart og flytende.
2☐ Jeg har lette vansker med å snakke, f.eks. famler av og til etter ord, mumler eller endrer stemmeleiet.
3☐ Jeg kan gjøre meg forstått, men min tale er f.eks. oppstykket, nølende, stotrende eller stammende.
4☐ De fleste mennesker har store vansker med å forstå hva jeg sier.
5☐ Jeg kan bare gjøre meg forstått med fakter.

SPØRSMÅL 8. **VANNLATING/AVFØRING**

1☐ Min blære og tarm fungerer normalt og uten problemer.
2☐ Jeg har lette problemer med min blære- og/eller tarmfunksjon, f.eks. vansker med å urinere, eller løs eller hard avføring.
3☐ Jeg har betydelige problemer med min blære- og/eller tarmfunksjon, f.eks. "uhell" av og til, eller alvorlig forstoppelse eller diaré.
4☐ Jeg har alvorlige problemer med min blære- og/eller tarmfunksjon, f.eks. regelmessig "uhell", eller behov for kateterisering eller klyster.
5☐ Jeg har ikke kontroll over min blære- og/eller tarmfunksjon.

SPØRSMÅL 9. **VANLIGE AKTIVITETER**

1☐ Jeg er i stand til å utføre mine vanlige aktiviteter (f.eks. arbeid, studier, husarbeid, fritidsaktiviteter) uten vanskelighet.
2☐ Jeg er i stand til å utføre mine vanlige aktiviteter noe mindre effektivt eller med litt vanskelighet.
3☐ Jeg er i stand til å utføre mine vanlige aktiviteter mye mindre effektivt, med betydelig vanskelighet, eller ikke fullt ut.
4☐ Jeg kan bare klare en liten del av mine vanlige aktiviteter fra tidligere.
5☐ Jeg er ute av stand til å klare noen av mine vanlige aktiviteter fra tidligere.

SPØRSMÅL 10. **MENTAL FUNKSJON**

1☐ Jeg er i stand til å tenke klart og logisk, min hukommelse fungerer godt.
2☐ Jeg har litt vansker med å tenke klart og logisk, min hukommelse svikter meg av og til.
3☐ Jeg har merkbare vansker med å tenke klart og logisk, min hukommelse er noe redusert.
4☐ Jeg har store vansker med å tenke klart og logisk, min hukommelse er betydelig nedsatt.
5☐ Jeg er stadig forvirret og desorientert for sted og tid.

SPØRSMÅL 11. **UBEHAG OG SYMPTOMER**

1☐ Jeg har ikke fysisk ubehag eller plager, f.eks. smerte, verk, kvalme, kløe etc.
2☐ Jeg har lett fysisk ubehag eller plager, f.eks. smerte, verk, kvalme, kløe etc.
3☐ Jeg har tydelig fysisk ubehag eller plager, f.eks. smerte, verk, kvalme, kløe etc.
4☐ Jeg har alvorlig fysisk ubehag eller plager, f.eks. smerte, verk, kvalme, kløe etc.
5☐ Jeg har uholdbart fysisk ubehag eller plager, f.eks. smerte, verk, kvalme, kløe etc.

SPØRSMÅL 12. **DEPRESJON**

1☐ Jeg føler meg overhodet ikke trist, melankolsk eller deprimert.
2☐ Jeg føler meg litt trist, melankolsk eller deprimert.
3☐ Jeg føler meg middels trist, melankolsk eller deprimert.
4☐ Jeg føler meg svært trist, melankolsk eller deprimert.
5☐ Jeg føler meg ekstremt trist, melankolsk eller deprimert.

SPØRSMÅL 13. **STRESS**

1☐ Jeg føler meg overhodet ikke engstelig, stresset eller nervøs.
2☐ Jeg føler meg litt engstelig, stresset eller nervøs.
3☐ Jeg føler meg middels engstelig, stresset eller nervøs.
4☐ Jeg føler meg svært engstelig, stresset eller nervøs.
5☐ Jeg føler meg ekstremt engstelig, stresset eller nervøs.

SPØRSMÅL 14. **LIVSKRAFT**

1☐ Jeg føler meg frisk og energisk.
2☐ Jeg føler meg litt sliten, trett eller svak.
3☐ Jeg føler meg middels sliten, trett eller svak.
4☐ Jeg føler meg svært sliten, trett eller svak, nesten utslitt.
5☐ Jeg føler meg ekstremt sliten, trett eller svak, totalt utslitt.

SPØRSMÅL 15. **SEKSUELL AKTIVITET**

1 ☐ Min helsetilstand har ingen ugunstig virkning på min seksuelle aktivitet.
2 ☐ Min helsetilstand har en liten virkning på min seksuelle aktivitet.
3 ☐ Min helsetilstand har en betydelig virkning på min seksuelle aktivitet.
4 ☐ Min helsetilstand gjør seksuell aktivitet nesten umulig
5 ☐ Min helsetilstand gjør seksuell aktivitet umulig.

NOEN FLERE SPØRSMÅL OM LIVSKVALITET

Vis hvilke utsagn som passer best på din helsetilstand i dag ved å sette et kryss i en av rutene utenfor hver av gruppene nedenfor.

Gange

Jeg har ingen problemer med å gå omkring. ☐

Jeg har litt problemer med å gå omkring. ☐

Jeg er sengeliggende. ☐

Personlig stell

Jeg har ingen problemer med personlig stell. ☐

Jeg har litt problemer med å vaske meg eller kle meg. ☐

Jeg er ute av stand til å vaske meg eller kle meg. ☐

Vanlige gjøremål *(for eksempel arbeid, studier, husarbeid, familie- eller fritidsaktiviteter)*

Jeg har ingen problemer med å utføre mine vanlige gjøremål. ☐

Jeg har litt problemer med å utføre mine vanlige gjøremål. ☐

Jeg er ute av stand til å utføre mine vanlige gjøremål. ☐

Smerte/ubehag

Jeg har verken smerte eller ubehag. ☐

Jeg har moderat smerte eller ubehag. ☐

Jeg har sterk smerte eller ubehag. ☐

Angst/depresjon

Jeg er verken engstelig eller deprimert. ☐

Jeg er noe engstelig eller deprimert. ☐

Jeg er svært engstelig eller deprimert. ☐

Diakonhjemmet Sykehus

SF-36 SPØRRESKJEMA OM HELSE

INSTRUKSJON: Dette spørreskjemaet spør om hvordan du ser på din egen helse. Disse opplysningene vil hjelpe oss til å få vite hvordan du har det og hvordan du er i stand til å utføre dine daglige gjøremål.

Hvert spørsmål skal besvares ved å krysse av det alternativet som passer best for deg. Hvis du er usikker på hva du skal svare, vennligst svar så godt du kan.

---

1 Stort sett, vil du si helsen din er:

(Kryss av ett alternativ)
1 ☐ Utmerket
2 ☐ Meget god
3 ☐ God
4 ☐ Ganske god
5 ☐ Dårlig

2 Sammenlignet med for ett år siden, hvordan vil du si helsen din stort sett er nå?

(Kryss av ett alternativ)
1 ☐ Mye bedre nå enn for ett år siden
2 ☐ Litt bedre nå enn for ett år siden
3 ☐ Omtrent den samme som for ett år siden
4 ☐ Litt dårligere nå enn for ett år siden
5 ☐ Mye dårligere nå enn for ett år siden

3 De neste spørsmålene handler om aktiviteter som du kanskje utfører i løpet av en vanlig dag. Er helsen din slik at den begrenser deg i utførelsen av disse aktivitetene nå? Hvis ja, hvor mye?

(Kryss av ett alternativ på hver linje)

	**Ja, begrenser meg mye**	**Ja, begrenser meg litt**	**Nei, begrenser meg ikke i det hele tatt**
a. Anstrengende aktiviteter som å løpe, løfte tunge gjenstander, delta i anstrengende idrett	1 ☐	2 ☐	3 ☐
b. Moderate aktiviteter som å flytte et bord, støvsuge, gå tur eller drive med hagearbeid	1 ☐	2 ☐	3 ☐
c. Løfte eller bære en handlekurv	1 ☐	2 ☐	3 ☐
d. Gå opp trappen flere etasjer	1 ☐	2 ☐	3 ☐
e. Gå opp trappen en etasje	1 ☐	2 ☐	3 ☐
f. Bøye deg eller sitte på huk	1 ☐	2 ☐	3 ☐
g. Gå mer enn to kilometer	1 ☐	2 ☐	3 ☐
h. Gå noen hundre meter	1 ☐	2 ☐	3 ☐
i. Gå hundre meter	1 ☐	2 ☐	3 ☐
j. Vaske deg eller kle på deg	1 ☐	2 ☐	3 ☐

4 I løpet av de siste 4 ukene, har du hatt noen av følgende problemer i ditt arbeid eller i andre av dine daglige gjøremål på grunn av din fysiske helse?

(Kryss av ett alternativ på hver linje)

	**JA**	**NEI**
a. Har du redusert tiden du har brukt på arbeidet ditt eller andre aktiviteter	1 ☐	2 ☐
b. Har du utrettet mindre enn du hadde ønsket	1 ☐	2 ☐
c. Har du vært hindret i visse typer arbeid eller andre aktiviteter	1 ☐	2 ☐
d. Har du hatt vanskeligheter med å utføre arbeidet ditt eller andre aktiviteter (f.eks. fordi det krevde ekstra anstrengelser)	1 ☐	2 ☐

5 I løpet av de siste 4 ukene, har du hatt noen av følgende problemer i ditt arbeid eller i andre av dine daglige gjøremål på grunn av følelsesmessige problemer (f.eks. fordi du har følt deg deprimert eller engstelig)?

(Kryss av ett alternativ på hver linje)

	**JA**	**NEI**
a. Har du redusert tiden du har brukt på arbeidet ditt eller andre aktiviteter	1 ☐	2 ☐
b. Har du utrettet mindre enn du hadde ønsket	1 ☐	2 ☐
c. Har ikke arbeidet eller utført andre aktiviteter like nøye som vanlig	1 ☐	2 ☐

6 I løpet av de siste 4 ukene, i hvilken grad har din fysiske helse eller følelsesmessige problemer hatt innvirkning på din vanlige sosiale omgang med familie, venner, naboer eller foreninger?

(Kryss av ett alternativ)
1 ☐ Ikke i det hele tatt
2 ☐ Litt
3 ☐ En del
4 ☐ Mye
5 ☐ Svært mye

7 Hvor sterke kroppslige smerter har du hatt i løpet av de siste 4 ukene?

(Kryss av ett alternativ)
1 ☐ Ingen
2 ☐ Meget svake
3 ☐ Svake
4 ☐ Moderate
5 ☐ Sterke
6 ☐ Meget sterke

8 I løpet av de siste 4 ukene, hvor mye har smerter påvirket ditt vanlige arbeid (gjelder både arbeid utenfor hjemmet og husarbeid)?

(Kryss av ett alternativ)
1 ☐ Ikke i det hele tatt
2 ☐ Litt
3 ☐ En del
4 ☐ Mye
5 ☐ Svært mye

9 De neste spørsmålene handler om hvordan du har følt deg og hvordan du har hatt det de siste 4 ukene. For hvert spørsmål, vennligst velg det svaralternativet som best beskriver hvordan du har hatt det. Hvor ofte i løpet av de siste 4 ukene har du:

(Kryss av ett alternativ på hver linje)

	**Hele tiden**	**Nesten hele tiden**	**Mye av tiden**	**En del av tiden**	**Litt av tiden**	**Ikke i det hele tatt**
a. Følt deg full av tiltakslyst?	1 ☐	2 ☐	3 ☐	4 ☐	5 ☐	6 ☐
b. Følt deg veldig nervøs?	1 ☐	2 ☐	3 ☐	4 ☐	5 ☐	6 ☐
c. Vært så langt nede at ingenting har kunnet muntre deg opp?	1 ☐	2 ☐	3 ☐	4 ☐	5 ☐	6 ☐
d. Følt deg rolig og harmonisk?	1 ☐	2 ☐	3 ☐	4 ☐	5 ☐	6 ☐
e. Hatt mye overskudd?	1 ☐	2 ☐	3 ☐	4 ☐	5 ☐	6 ☐
f. Følt deg nedfor og trist?	1 ☐	2 ☐	3 ☐	4 ☐	5 ☐	6 ☐
g. Følt deg sliten?	1 ☐	2 ☐	3 ☐	4 ☐	5 ☐	6 ☐
h. Følt deg glad?	1 ☐	2 ☐	3 ☐	4 ☐	5 ☐	6 ☐
i. Følt deg trett?	1 ☐	2 ☐	3 ☐	4 ☐	5 ☐	6 ☐

10 I løpet av de siste 4 ukene, hvor mye av tiden har din fysiske helse eller følelsesmessige problemer påvirket din sosiale omgang (som det å besøke venner, slektninger osv.)?

(Kryss av ett alternativ)
1 ☐ Hele tiden
2 ☐ Nesten hele tiden
3 ☐ En del av tiden
4 ☐ Litt av tiden
5 ☐ Ikke i det hele tatt

11 Hvor RIKTIG eller GAL er hver av de følgende påstander for deg?

(Kryss av ett alternativ på hver linje)

**Påstander om din helse**	**Helt riktig**	**Delvis riktig**	**Vet ikke**	**Delvis gal**	**Helt gal**
a. Det virker som om jeg blir lettere syk enn andre	1 ☐	2 ☐	3 ☐	4 ☐	5 ☐
b. Jeg er like frisk som de fleste jeg kjenner	1 ☐	2 ☐	3 ☐	4 ☐	5 ☐
c. Jeg forventer at helsen min vil bli dårligere	1 ☐	2 ☐	3 ☐	4 ☐	5 ☐
d. Helsen min er utmerket	1 ☐	2 ☐	3 ☐	4 ☐	5 ☐

Appendix 1B
BASDAI

Spørsmålene nedenfor gjelder hvordan du følte deg den siste uken. Marker ditt svar med å krysse i en rute.

1. Hvordan vil du beskrive den generelle graden av utmattelse/tretthet du har erfart?

0	-	1	-	2	-	3	-	4	-	5	-	6	-	7	-	8	-	9	-	10

Ingen — Svært høy

2. Hvordan vil du beskrive den generelle graden av smerter i nakke-, rygg eller hofter i forbindelse med Bekhterev sykdom?

0	-	1	-	2	-	3	-	4	-	5	-	6	-	7	-	8	-	9	-	10

Ingen — Svært høy

3. Hvordan vil du beskrive det generelle nivået av smerter/hevelse du har hatt i andre ledd enn nakken- ryggen eller hoftene?

0	-	1	-	2	-	3	-	4	-	5	-	6	-	7	-	8	-	9	-	10

Ingen — Svært høy

4. Hvordan vil du beskrive den generelle graden av ubehag du har hatt på eventuelle steder som gjør vondt ved berøring eller trykk?

0	-	1	-	2	-	3	-	4	-	5	-	6	-	7	-	8	-	9	-	10

Ingen — Svært høy

5. Hvordan vil du beskrive den generelle graden av stivhet du har opplevd om morgenen fra det tidspunktet du våkner?

0	-	1	-	2	-	3	-	4	-	5	-	6	-	7	-	8	-	9	-	10

Ingen — Svært høy

6. Hvor lenge varer morgenstivheten fra det tidspunktet du våkner?

0	-	1	-	2	-	3	-	4	-	5	-	6	-	7	-	8	-	9	-	10

0 timer — 1 time — 2 timer eller mer

Appendix 1B

BASFI

Spørsmålene nedenfor gjelder hvordan du følte deg <u>den siste uken.</u> Marker ditt svar med å krysse av i en rute.

1. Ta på strømper eller strømpebukser uten assistanse eller ved bruk av hjelpemidler (for eksempel strømpe påtrekker)

0	-	1	-	2	-	3	-	4	-	5	-	6	-	7	-	8	-	9	-	10
Lett																				Umulig

2. Bøye deg fremover fra midjen for å plukke opp en penn fra gulvet uten å bruke et hjelpemiddel

0	-	1	-	2	-	3	-	4	-	5	-	6	-	7	-	8	-	9	-	10
Lett																				Umulig

3. Nå opp til en høythengende hylle uten bruk av hjelpemidler (for eksempel gripetang)

0	-	1	-	2	-	3	-	4	-	5	-	6	-	7	-	8	-	9	-	10
Lett																				Umulig

4. Reise deg fra en spisebordsstol uten armlener eller annen hjelp

0	-	1	-	2	-	3	-	4	-	5	-	6	-	7	-	8	-	9	-	10
Lett																				Umulig

5. Reise deg opp fra liggende stilling på gulvet uten hjelp

0	-	1	-	2	-	3	-	4	-	5	-	6	-	7	-	8	-	9	-	10
Lett																				Umulig

6. Stå oppreist uten støtte i 10 min. uten å få ubehag

0	-	1	-	2	-	3	-	4	-	5	-	6	-	7	-	8	-	9	-	10
Lett																				Umulig

BASFI fortsettelse.

7. Gå opp 12-15 trappetrinn uten å bruke rekkverk eller gåstøtte. En fot på hvert trinn

0 - 1 - 2 - 3 - 4 - 5 - 6 - 7 - 8 - 9 - 10

Lett Umulig

8. Se deg over skulderen uten å vri kroppen

0 - 1 - 2 - 3 - 4 - 5 - 6 - 7 - 8 - 9 - 10

Lett Umulig

9. Utføre fysisk krevende aktiviteter (for eksempel fysioterapiøvelser, hagearbeid eller sport)

0 - 1 - 2 - 3 - 4 - 5 - 6 - 7 - 8 - 9 - 10

Lett Umulig

10. Utføre en hel dags aktiviteter enten hjemme eller på arbeid

0 - 1 - 2 - 3 - 4 - 5 - 6 - 7 - 8 - 9 - 10

Lett Umulig

Reference List

(1) Garrett S, Jenkinson T, Kennedy LG, Whitelock H, Gaisford P, Calin A. A new approach to defining disease status in ankylosing spondylitis: the Bath Ankylosing Spondylitis Disease Activity Index. J Rheumatol 1994 Dec;21(12):2286-91.

(2) Calin A, Garrett S, Whitelock H, Kennedy LG, O'Hea J, Mallorie P, et al. A new approach to defining functional ability in ankylosing spondylitis: the development of the Bath Ankylosing Spondylitis Functional Index. J Rheumatol 1994 Dec;21(12):2281-5.

Diakonhjemmet Sykehus

Appendix 2

Kjære pasient

Som ledd i din konsultasjon hos hjertespesialist, ber vi om at du fyller ut vedlagte skjema og tar det med til undersøkelsen på poliklinikken.

Det er viktig at du setter et kryss enten i Ja eller Nei boksen.

Det at du besvarer disse spørsmålene vil gjøre det lettere og raskere for legen å fokuserer på dine hoved problemer. Samtidig får legen en god oversikt over dine risiko faktorer for hjerte-kar sykdom og din generelle helse tilstand.

Ditt navn: **Fødselsnummer:**

Jeg har (SETT RING RUNDT) **Revmatoid artritt – Behkterew – Psoriasis**

Jeg fikk diagnosen (årstall)_______________
Jeg har hatt symptomer på sykdommen siden (årstall)**:**_________i hvor mange år?_____

Hvor gammel er du nu: _________år
Høyde:_________cm
Vekt:___________kg

	Ja	Nei
For kvinner:<br>Alder når sluttet med menstruasjon _____________år gammel		
Østrogen bruk etter menopause:	☐	☐
Har du brukt P-pille	☐	☐

**SPØRSMÅL OM HJERTESYKDOM** - **Symptomer:**	Ja	Nei
Har du hatt ett eller flere **anfall av flimmerhjerte**? (paroxystisk atrieflimmer)	☐	☐
Har du **kronisk flimmer hjerte** (atrie flimmer)		
Har du fått diagnosen **hjertesvikt?**	☐	☐
Blir du tungpustethet utover det normale ved gange opp trapp/bakke?	☐	☐
Blir du tungpusten av lett husarbeid/gange flatt?	☐	☐
Må du sove med flere puter om natten fordi du er tungpusten?	☐	☐
Har du problemer med hovne ben?	☐	☐
	Ja	Nei
Har du noen gang hatt **angina pektoris** (**brystsmerter/ubehag i brystet**).<br>Dersom nei, gå rett til spm om hjerteinfark på neste side	☐	☐

		Ja	Nei
**Hvis brystsmerter/ubehag** er de i:	Klemmende	☐	☐
	Stikninger	☐	☐
	Trykk	☐	☐
	Skjærende	☐	☐
	Press	☐	☐
	Tungt	☐	☐
	Bånd rund brystet	☐	☐
	Kvalme, uvelhet	☐	☐
**Hvor** er brystsmertene/ubehaget lokalisert?		Ja	Nei

	Midt i brystet, bak brystbenet	☐	☐
	På en av sidene av brystet	☐	☐
	Halsen eller kjeven	☐	☐
	Ut i armene	☐	☐
	Ut i ryggen	☐	☐
**Når** får du brystsmertene/ubehaget ?		Ja	Nei
	Gange i trapp, motbakke	☐	☐
	Gange flatt underlag, innendørs	☐	☐
	Ved lett husarbeide	☐	☐
	I hvile, sitter stille	☐	☐
	Om natten	☐	☐
**Hvor ofte** får du brystsmerter/ubehag?		Ja	Nei
	Månedlig eller sjeldnere	☐	☐
	Ukentlig	☐	☐
	Flere ganger pr uke	☐	☐
	Daglig	☐	☐
	Flere ganger daglig evt. hvor mange ganger pr dag ______		
**Hvor lenge** varer anfallene?		Ja	Nei
	Sekunder	☐	☐
	Få min	☐	☐
	½ time eller mer	☐	☐
Har du forsøkt **Nitroglycerin**?		☐	☐
Gikk brystsmertene over med Nitroglycerin?		☐	☐
Hvis Ja, hvor lang tid tok det	Noen minutter	☐	☐
	Mer enn 15 min	☐	☐
Har du noen gang hatt **hjerteinfarkt**?		☐	☐
	Hvis ja, årstall?________		
Er du blitt **hjerteoperert**?		☐	☐
Hvis Ja, årstall?_________			
Operert med **utblokking** av hjertets kransårer?		☐	☐
	Hvis Ja, årstall?_________		
Operert med **bypassoperasjon**?		☐	☐
	Hvis Ja, årstall?:_________		
Operert på **hjertets klaffer**?		☐	☐
	Hvis Ja, årstall?_________		
Har du **trange pulsårer til bena**, f.eks. smerter i tykkleggen i motbakke som går over når du stopper opp.		☐	☐
Er du operert for dette	Hvis Ja, årstall?_________		
		Ja	Nei
**Har du hatt:**	Hjerneblødning	☐	☐
	Hjerneslag	☐	☐
	Drypp (TIA)	☐	☐
	Difteri	☐	☐
	Skarlagens feber	☐	☐
	Gikt feber	☐	☐

**Arvelige forhold relatert til hjertesykdom**	Ja	Nei
Har din mor /far /søster (re) eller bror (brødre) har hatt hjerteinfarkt?	☐	☐
Hvis ja, hvor gammel var han/hun ved sitt første infarkt ?		
		Alder
Mor	☐	
Far	☐	
Søster	☐	
Bror	☐	
	Ja	Nei
Har din mor /far /søster (re) / bror (brødre) dødd plutselig uten kjent grunn?	☐	☐

Spørsmål om trening

Hvor ofte trener du (øket puls og pust) i minst en halv time (30 minutter)?
Kryss av bare en gang

- ☐ 3 eller flere ganger per uke
- ☐ 1-2 ganger per uke
- ☐ 1-2 ganger per måned
- ☐ Trener ikke regelmessig
- ☐ Kan ikke trene pga nedsatt funksjon/handicap

**Spørsmål om røyking** (Kryss av bare en gang) **:**	**Ja**
Jeg har sluttet > 6 mndr siden	☐
Jeg røyker daglig	☐
Jeg har aldri røkt	☐
**Alkohol forbruk** (Kryss av bare en gang) **:**	
Daglig	☐
Ukentlig	☐
Månedlig	☐
Aldri	☐

Diakonhjemmet Sykehus

Har du / har du hatt andre sykdommer som:

		Ja	Nei
Høyt blodtrykk eller behandling for høyt blodtrykk		☐	☐
Diabetes (sukkersyke)		☐	☐
Tuberkulose		☐	☐
HIV / AIDS		☐	☐
Astma, KOLS, emfysem, annen lungelidelse		☐	☐
Allergi, høysnue, eksem		☐	☐
Medikament allergi		☐	☐
Isjias		☐	☐
Kreftsykdom		☐	☐
Evt Hvor ______________________________			
Nevrologisk sykdom (sykdom i hjerne eller nervevev)		☐	☐
Stoffskiftesykdom		☐	☐
Mental lidelse		☐	☐
Overforbruk av alkohol eller narkotika		☐	☐
Nyresykdom		☐	☐
Leversykdom		☐	☐
Tarmsykdommer	hatt mavesår	☐	☐
	Ulcerøs colitt	☐	☐
	Chron's sykdom	☐	☐
Blodmangel (anemi) eller annen blodsykdom		☐	☐
Epilepsi		☐	☐
Hatt Blodpropp i ben		☐	☐
Hatt Blodpropp i lunge		☐	☐
Regnbuehinne betennelse (r)		☐	☐
	Antall: 1	☐	☐
	2-5	☐	☐
	>5	☐	☐

	Ja	Nei
**Andre immunsykdommer**	☐	☐

Før evt. opp hvilke(n) (Polymylagia revmatika, Sjøgrens sykdom, SLE, Lichen ruber.., Vitiligo osv)

1.

2.

3.

4.

Før opp andre kroniske sykdommer eller operasjoner du har fått utført nedenfor:

1.

2.

3.

Medikamenter **(kryss av i 1 rute)**			
Prednisolon **(sett 1 kryss)**	Aldri brukt:	Brukt tidligere:	Bruker nå:
	☐	☐	☐
Osteoporosemedisiner **(medisiner mot benskjørhet)**	Aldri brukt:	Brukt tidligere:	Bruker nå:
**Østrogener**	☐	☐	☐
**Bisfosfonater** (Aredia, Fosamax Optinate Zometa)	☐	☐	☐
**Forsteo**	☐	☐	☐
**Calcitonin**	☐	☐	☐
**Kalktabletter**	☐	☐	☐
**D-vitamintilskudd (feks i tran, omega-3)**	☐	☐	☐
Betennelsesdempende medisiner			
	Aldri brukt:	Brukt tidligere:	Bruker nå:
**Medikamenter i gruppen:** Naproxen (NaprenE Naprosyn Naproxen)	☐	☐	☐
**Medikamenter i gruppen:** Voltaren Pirox, Mobic, Brufen, Ibux, Ibumetin, Orudis	☐	☐	☐
**Medikamenter i gruppen:** Celebra, Arcoxia, Vioxx, Bextra	☐	☐	☐

Diakonhjemmet
Sykehus

Hjertemedisiner

	Aldri brukt:	Brukt tidligere:	Bruker nå:
**Marevan**	☐	☐	☐
**Albyl- E**	☐	☐	☐
**Plavix**	☐	☐	☐
**Kolesterolsenkende**<br>Sett ring rundt den du bruker nå. Skriv hvor mange **mg** per dag ____________________<br>(eks Simvastatin, Lipitor, Pravachol, Crestor, Lescol, Ezetrol, Niaspan m.m.	☐	☐	☐

	Aldri brukt:	Brukt tidligere:	Bruker nå:
**Betablokker**<br>(eks Selozok/Metoprolol, Emconcor, Tenormin, Kredex, Inderal, Pranolol, Atenolol, Sotalol)	☐	☐	☐
**Calciumblokker**<br>(eks Isoptin, Verapamil, Adalat, Norvasc mm)	☐	☐	☐
**ACE hemmer**<br>(eks Lisinopril/Zestril, Enalapril/Renitec, Ramipiril/Triatec	☐	☐	☐
**evt i komb med vanndrivende**(hydroklorthiazid)<br>(eks Enalapril comp, Zestoretic, Zestoretic mite, Lisinopril Hydroklortiazid, Renitec comp, Renitec comp. mite	☐	☐	☐
**All antagonister**<br>(eks Aprovel, Diovan, Atacand, Cozaar, Teveten, Micardis, Olmetec osv)	☐	☐	☐
**evt i komb med vanndrivende:**<br>(Eks AtacandPlus,CozaarComp, CozaarCompForte, CoAprovel, MicardisPlus etc)	☐	☐	☐
**Vanndrivende**<br>(eks Diural, Furix, Normorix, Burinex, Spironolaktone, Eplerenone mm)	☐	☐	☐

Liste over alle medikamenter:

1.
2.
3.
4.
5.
6.
7.
8.
9.
10.
11.

RORA Appendix 3
Transthorakal ekkokardiografisk prosedyre :

17 forskningsrelaterte looper/bilder. Angitte looper og hjertecycler (bilder) er et MINIMUMSTALL. Alle bilder/looper i 50mm/sec. Alle opptak tas i venstre sideleie i endeekspirasjon mens probanden holder pusten. Viktig mht 2-D strain analyser. 2-D opptakene må derfor også ha mest mulig synlig endocard. Opptakene nedenfor skal IKKE analyseres.

	**Projeksjon**	**Modus**	**Hensikt/Nytte/Målinger som kan gjøres**	**Opptak**
1	Parasternal langakse	2-D	Ve. ventrikkel (VV), hø. ventrikkel (HV), ve. atrium (VA), ao.klaff og ao.ascendens. Pericard. Ekstra opptak dersom ikke gode samtidige VV og VA opptak	3 looper
2	Parasternal langakse	M-mode	Måle dimensjon av VV, HV og VA	1 bilde med minst 5 hjertecycler,
3 - 5	Parasternal kortakse	2-D	Mitralventilnivå, papillenivå og apikalt nivå for å måle rotasjon/torsjon. Apikal loop oppnås enklest ved å gå ut fra 4C, vipp proben ned og før den ofte litt proximalt til du får en god loop. Nesten lukning i endesystole.	3 looper på hvert av de 3 nivåene
6	Parasternal kortakse	M-mode	Papillenivå, Måle dimensjon av VV og VVmasse	Et bilde med minst 5 hjertecycler
7	Apikal 4-kammer (4C)	2-D	Framstille VV, HV, VA og HA (dvs "rene" 4C looper). Måle: volum i endesystole (es) og endediastole (ed) av VV (4C og 2C ad modum Simpson`s), volum av VA i es (areal - lengde, biplan 4C og 2C), areal av HA i es..<br>Måling av 2-D strain (Godt synlig endocard)	3 looper (som regel 2 x 3 looper, dersom HV og HA skal godt med)
8	Apikal 4-kammer	Pulset Doppler	Transmitral E og A for vurdering av VV diastoliske funksjon. Måle max. hastighet av E og A og decelerasjonstiden av E.<br>Obs: Det er svært viktig at målepunktet (sample volume) settes på Mitraltipnivå.	Et bilde minst 5 hjertecycler
9	Apikal 4-kammer	M-mode	TAPSE (tricuspid annular plane systolic excursion) reflekterer systolisk funksjon av HV.	Et bilde minst 5 hjertecycler,
10	Apikal 4-kammer	CW	Dersom Tricuspidalinsuffisiens til stede, forsøk å få fram maximumhastigheten av denne. Ikke bruk mye tid på dette.	Et bilde med minst 5 hjertecycler
11	Apikal 4-kammer	TVI	Mulighet til å vurdere systolisk og diastoliske vevs-Doppler hastigheter i VV og HV. Dersom ikke både HV og VV er med på samme loop, lag et ekstra sett med minst 3 looper, hvor HV kommer fram. Obs: FRP>100/s	3 looper (som regel 2 x 3 looper, dersom HV og HA skal med)
12-13	Apikal 4-kammer	TVI + Pulset Doppler	E` ved septale- og laterale mitralfeste. (Middle disse til beregning av E/E`.)	2 bilder med minst 5 hjertecycler,
14	Apikal 4-kammer	TVI + Pulset Doppler	Systolisk max hastighet (S bølge) ved laterale tricuspidalfeste (reflekterer HV funksjon)	Samme
15	Apikal 2-kammer (2C)	2-D	Måle volum av VV og VA og 2-D strain. Dersom dårlig framstilling av VA, gjør ekstra opptak.	3 looper
16	Apical langakse	2-D	Må med pga AFI 2-D strain metodikken	3 looper
17	Subcostal	2-D	Vurdering av hø. ventrikkel og pericard	3 looper

RORA Appendix 4

Protocol for Multi Detector Computed Tomography coronary angiography:

Vulnerable carotid and coronary artery plaques and **RO**suvastatin treatment in **R**heumatoid **A**rthritis, ankylosing spondylitis and other inflammatory joint diseases

The RORA study

Nils E. Kløw[1], AG Semb[2], Stephen Nicholls[3], Department of Cardiology[1], Oslo University Hospital, Ullevål, Department of Rheumatology[2], Diakonhjemmet Hospital, Oslo, Norway and Department of Cardiovascular Medicine[3], Cleveland Clinic, Cleveland, Ohio, USA

Version 28.09.09

Multi Detector Computed Tomography (MDCT) is a non invasive diagnostic method, and provides valuable information of the coronary artery stenoses, coronary plaques and calcification.

We will analyze:

4. **Coronary calcium score-Agatston score.**
5. **CT coronary angiography to evaluate for stenoses and plaques. The step-and-shoot technique will be the preferred method to keep the exposure as low as possible. Beta blocker and diazepam will be given to scan when the heart rate is below 60 /min. The Step-and-shoot procedure will have a high sensitivity and specificity in approximately 60% of the patients.**
6. **MDCT during the full heart cycle will be performed in those patients with heart rate above 60/min or when the step-and-shoot procedure is suboptimal.**

There are 3 possible outcome of the MDCT:

1) Normal coronary arteries
2) Coronary plaques
3) Stenoses present, suspected stenoses or the study cannot exclude stenoses for technical reasons.

1. **The patients with normal coronary arteries will not have MDCT after 18 months.**
2. **Patients with plaques without suspected stenoses will have follow-up CT after 18 months**
3. **Patients with stenoses present, suspected stenoses or the study cannot exclude stenoses for technical reasons will be offered the opportunity to undergo selective coronary angiography and IVUS before statin therapy. After 18 months follow up coronary angiography and CT will be performed.**

The patients will be not be given an intervention during the coronary angiography, but all patients with significant stenoses will be evaluated individually for possible intervention.

Appendix 5

Protocol for Coronary intravascular ultrasound (IVUS) examination in:

Vulnerable carotid and coronary artery plaques and Rosuvastatin treatment in rheumatoid arthritis, ankylosing spondylitis and other inflammatory joint diseases

The RORA study

Nils E. Kløw[1], AG Semb[2], Stephen Nicholls[3,] Department of Cardiology[1], Oslo University Hospital, Ullevål, Department of Rheumatology[2], Diakonhjemmet Hospital, Oslo, Norway and Department of Cardiovascular Medicine[3], Cleveland Clinic, Cleveland, Ohio, USA

Version 28.09.09

Intravascular ultrasound (IVUS) is the gold standard for evaluation of coronary plaque, lumen, and vessel dimensions. Serial IVUS imaging and measurement can be employed to determine changes in atheroma burden, and recently Nissen et al (JAMA 2006;295:1556) utilized IVUS to demonstrate that statin therapy with high dose Rosuvastatin can regress atherosclerosis in patients with coronary artery disease. However, it is unknown whether this drug can also cause regression of atherosclerosis among patients with rheumatoid arthritis.

Intravascular ultrasound (IVUS) imaging will be performed (Boston Scientific, iLab) at baseline and after 18 months to assess coronary atheroma proliferation/regression. IVUS data will be recorded with a high frequency (40 MHz) ultrasound transducer. Following administration of 200 µg nitroglycerin (intra coronary) the transducer will be positioned at the distal-most segment of the target artery (usually LAD vessel) using standard angiographic techniques before automated pullback is commenced (speed 0.5 mm/second). The start position of the IVUS transducer will be documented on cine film immediately prior to pullback being commenced.

The target coronary artery for IVUS will be decided according to detection of stenoses and plaques on the CT and the coronary angiography.

The total number of frames available for analysis for each patient will vary and be dependent on the length of vessel segment imaged and the frame acquisition rate (number of frames recorded per second). Frames at 1 mm intervals will therefore be selected and measured using an off-line software program (eg QIVUS by Medis).

The following variables will be measured comparing baseline and follow up (18 mo) examinations:

1. Intimal, lumen and external elastic membrane (EEM) areas
2. Total atheroma volume
3. Percent atheroma volume
4. Maximal intimal thickness (MIT) and the number of patients with an increase of MIT > 0.3 mm
5. Virtual Histology tool (Boston Scientific) will also be employed to assess development/progression of atheroma. This recently developed technique allows a

qualitative analysis of the intimal wall composition by spectral analysis of IVUS radio frequency data. In the VH analysis four histological tissue components (fibrous, fibro/fatty, necrotic core, and calcium) are correlated with the radio frequency data and given a colour code (green, greenish-yellow, red, and white, respectively). VH analyses have been found acceptably reproducible and will therefore be applied to data acquired in this study.

Appendiks 6
Translasjons prosjekter relatert til RORA

PROTOKOLL FOR SAMARBEIDSPROSJEKT MED ANNE GRETE SEMB

GRUPPELEDERE: MARIA KRUDTAA DAHLE, HILDE NEBB, GURO VALEN

MEDARBEIDERE: PETTER RISØE, APLE LEI, TORUN FLATEBØ, MARTE BLIKSØEN, LARS HENRIK MARIERO

Hensikt: Studere aktivering av inflammasjonsstatus, Toll Like Receptor (TLR)-mediert immunrespons og inflammasjonsmodulerende mekanismer i pasienter med ankyloserende spondylitt (bechterew) og revmatoid artritt. Inflammasjonsstatus og tre inflammasjonsmodulerende systemer skal studeres i materialet:

1) Inflammasjonsstatus (sirkulerende mitokondrielt DNA, cytokiner, inflammatorisk signalvei i monocytter og polymorfonukleære granulocytter (PMN) (fosfoproteiner, TLR9, NFkB) Ansvar Guro Valen.

2) Kjernereseptorene LXR/RXR-mediert inflammasjonsregulering og uttrykk av kjernereseptorer (mRNA/protein, Chip/EMSA). Ansvar Hilde Nebb.

3) cAMP-mediert inflammasjonsmodulering og uttrykk av adenylyl cyclaser (mRNA/protein) Ansvar Petter Risøe/Maria K. Dahle.

4) Uttrykk av CD300-reseptorer (mRNA/protein). Evt sammenheng til kjente SNPs. Ansvar Maria K. Dahle.

Delmål: Studere om det er forskjell mellom mannlige og kvinnelige pasienter
Delmål: Se om det er forskjeller mellom Bechterew/revmatoid artritt pasienter
Delmål: Se om det er forskjell mellom pasienter med leddsykdom og de som har tilleggsdiagnose med atherosklerose.
Delmål: Se om den presumptive aktiveringen av immunitet kan reverseres ved statiner

Materiale: 50 pasienter (11 med ankyloserende spondylitt, 31 med revmatoid artritt).

Kontroller: Vi skaffer et kontrollmateriale på IMB ved rekruttering av 40 friske frivillige personer over 50 år av begge kjønn. Kontrollen blir kun til basis nivå, ingen kontroll til statin behandling
Blodprøver tas
1) 9 ml blod til fullblodstimuleringsstudie, heparinblod, holdes varm i termos på vippebord
2) 9 ml blod (heparin vakutainer) til isolering av monocytter/PMN/DNA settes i kjøleskap før henting (6 ml blod brukes til positiv seleksjon CD14-kuler, etter å ha brukt polymorfoprep for isolering av PMN for isolering av RNA og protein. De siste 3 ml blod brukes til DNA-isolering fra plasma (total DNA hvor mitoDNA amplifiseres opp). Første prøver tas før statinbehandling, andre prøve tas etter 3 måneder med statiner.

Endepunkter:
<u>9 ml heparinblod til fullblodoppsett:</u>
Blodet fordeles med 0,6 ml per rør

1. DMSO (løsningsmiddel for agonister)
2. LXRα-agonist
3. LXRβ-agonist
4. 9CisRA (RXR-agonist)
5. atRA (RAR-agonist)
6. DMSO
7. LXRα-agonist
8. LXRβ-agonist
9. 9CisRA (RXR-agonist)

10. atRA (RAR-agonist)
11. Forskolin (AC activator)
12. 8-CPTcAMP
13. PGE2 (EP-reseptoragonist)

Etter 60 minutter tilsettes 10ng/ml LPS til prøve 6-11
Plasma høstes ved sentrifugering etter 6 timer. Analyseres for cytokiner med bioplex/ELISA.
9 ml heparinblod til RNA/protein/mitDNA
3 ml blod til mitDNA-isolering fra plasma
6 ml blod tas ut til direkte PMN og monocytt ekstraksjon for RNA/protein-isolering (Norgen kit)

Kontaktpersoner:
Turid: t-agerup@diakonsyk.no
Mobil: 91861877
Telefon: 22454046
Maria Krudtaa Dahle: m.k.dahle@medisin.uio.no
Mobil: 92612718
Guro Valen guro.valen@medisin.uio.no
Mobil 91346071
Hilde Nebb. h.i.nebb@medisin.uio.no
Mobil 41552925

Fremdriftsplan:
Innsamling av pasientprøver begynner august 2010, og 4 pasienter samles inn per uke. Fullblodsstimulering, celleisolering, og isolering av mitokondrielt DNA, protein og RNA gjøres samme dag. Det vil ta 10-11 uker å samle inn alt pasientmateriale før statin behandling. Kontrollmateriale vil samles inn i dager hvor det ikke finnes pasienter. De behandlede pasientene vil være klare til prøve nummer to omtrent når de første prøvene er innhentes, og vare fram til alle har stått på 3 måneders behandling – med tanke på jul vil vi være ferdige med materialinnhenting i slutten av februar.
Noen analyser kan gå parallelt med innsamling av materialet, mens andre analyser ikke kan påbegynnes før alt er ferdig innsamlet (gjelder særlig kvalitative metoder som Chip og EMSA). Vi har som målsetting å ha alt analysert til juli 2011.

Publisering:
Målsettingen er at dataene skal brukes til minst 4 ulike publikasjoner i internasjonale peer-review journaler. Avhengig av hvordan delmålene faller ut (er det kjønnsforskjeller i noen endepunkter, reduserer statiner inflammatoriske responser etc.) kan det tenkes at vi har materiale til enda flere publikasjoner. Vitenskapelig tyngde vil prioriteres framfor å få en stor menge artikler. De første artiklene vil være innsendingsklare høsten 2011.

Appendiks 7

Medikament prosedyre

Alle pasientene som har undertegnet informert samtykke, vil

Pas < eller lik 70 år

Starter med rosuvastatin 20 mg, 1 tablett pd. etter at de har fått utført alle undersøkelser beskrevet under baseline i tidsforløps-skjemaet.

Lipidstatus etter 14. dager. Opptitrere dosen til 40 mg x 1 dersom LDL nivået på 1.6-1.8 mmol/L ikke er nådd.

Pasienter >70 år

Startdose av rosuvastatin på 5 mg 1 tablett pd.

Lipidstatus hver 14. dag til LDL nivåer på 1.6-1.8 mmol/L er nådd. Dosen av rosuvastatin opptitreres hver 14. dag til LDL nivåer på 1.6-1.8 mmol/L er nådd. Dette gjøres ved dobling av dosen dvs. 10 mg x 1, 20 mg x 1, 40 mg x 1.

Målet er at alle deltagere skal ha nådd 1.6-1.8 mmol/L ved 3 måneder etter start i studien. De pasienter som ikke når mål nivåer av LDL vil ikke bli tatt ut av studiet, men fulgt videre per protokoll.

Deltagere vil stå på Rosuvastatin medikasjon i totalt 18 måneder.

Oslo 02.04.10

Endringsprotokoll for prosjektet:

"Kolesterolavleiring i arteria carotis og koronar arterier og effekten av Rosuvastatin ved revmatoid artritt, ankyloserende spondylitt og andre inflammatoriske artritter (RORA-studien)"

Ansvarlig: Jonny Hisdal, Fysiolog, PhD, Sirkulasjonsfysiologisk laboratorium, Oslo Vaskulære Senter, Aker Universitets Sykehus

I tillegg til undersøkelser allerede beskrevet i protokollen som er godkjent av regional etisk komite (2009/2219-1), søkes det herved om å få godkjenning til å utføre undersøkelse av pasientenes endotelfunksjon ved oppstart (besøk 1) og etter 18 måneder (besøk 8). Målingene det søkes om skal utføres på sirkulasjonsfysiologis laboratorium, Oslo universitetssykehus, Aker.

Arteriell endotelfunksjon – målt med FMD-metoden: Endringer i arterienes innerste cellelag, endotelet, regnes som en av de første direkte målbare markører på begynnende arteriosklerose i sirkulasjonssystemet. De seneste årene er det blitt utviklet en ny metode for å undersøke endotelfunksjon. Metoden går i korthet ut på at en ved hjelp av ultralyd måler diameteren på arteria brachialis på overarmen. Deretter klemmes blodstrømmen til armen av i 5 minutter. I avklemningsperioden produserer endotelet i arterien som er avklemt stoffer (blant annet NO) som gjør at blodåren i perioden like etter okklusjonen vil utvide seg (dilatere). Graden av dilatasjon i etterkant av okklusjonen viser seg å være en svært presis markør på endotelets funksjon. Redusert dilatasjon regnes som et tegn på redusert endotelfunksjon og begynnende arteriosklerose. Metoden beskrevet over kalles "Flow mediated dilatation" eller FMD-metoden, og regnes i dag som "gullstandard" for undersøkelse av endotelfunksjon. Metoden har blitt benyttet i en rekke studier siden den ble lansert i på begynnelsen av 1990 tallet. Metoden beskrives i detalj artikkelen "Guidelines for the Ultrasound Assewssment og Endothelial- Dependent Flow-Mediated Vasodilation of the Brachial Artery" av Coretti og medarbeidere publisert i J Am Coll Cardiol 2002;39:257-56. Ved sirkulasjonsfysiologisk laboratoruim, Oslo universitetssykehus, Aker, har metoden blitt benyttet i studier både på friske kontroller, pasienter med søvnapne og på pasienter med perifer arteriell sirkulasjonsvikt.
Selve perioden med avklemming av blodstrømmen til underarmen kan være litt smertefull, men de aller fleste tolererer perioden med avklemming godt. Dersom pasienten opplever smerten som så ubehagelig at han ikke ønsker å fortsette kan målingen raskt avsluttes ved at mansjetten rundt overarmen deflateres og smertene vil umiddelbart opphøre. Vi er ikke kjent med at det har forekommet komplikasjoner i forbindelse med bruk av denne metoden for å måle endotelfunksjon.

Oslo 03.04.10

Endringsprotokoll for prosjektet:

"Kolesterolavleiring i arteria carotis og koronar arterier og effekten av Rosuvastatin ved revmatoid artritt, ankyloserende spondylitt og andre inflammatoriske artritter (RORA-studien)"

Ansvarlig: Hilde Berner Hammer, MD, PhD, ass avd leder, Revmatologisk avd, Diakohjemmet sykehus

I tillegg til undersøkelser allerede beskrevet i protokollen som er godkjent av regional etisk komite (2009/2219-1), søkes det herved om å få godkjenning til å utføre ultralyd (UL) undersøkelse av ledd ved oppstart og etter 3 mnd av pasientene i studien som har revmatoid artritt (RA).
UL undersøkelsene vil foregå ved revmatologisk poliklinikk, Diakonhjemmet sykehus, dvs. samme poliklinikk som mesteparten av de øvrige undersøkelsene.
UL er en metode der lydbølger med høy frekvens benyttes til å avbilde betennelser i ledd. En benytter lydhoder som plasseres over leddene, og det er en stort sett smertefri metode uten kjente bivirkninger. Ved UL får en avbildet omfanget av synovitten i de enkelte ledd, og ved bruk av power Doppler (PD) kan en se graden av patologisk vaskularisering inne i synovitten. Det er funnet sammenheng mellom graden av PD og senere utvikling av skader i leddet.

Studieansvarlig for denne tilleggsundersøkelsen, har utarbeidet et atlas over de fleste ledd med en semi-kvantitativ skåring fra 0 til 3 av både synovitt og PD (0=normalt, 1=litt, 2=moderat, 3=mye). En nylig utført reliabilitetsstudie med bruk av dette atlaset som bakgrunn for skåring av artritten (både synovitt og PD) i 32 ledd viste svært høy reliabilitet. De 32 leddene som ble undersøkt var: MCP 1-5, håndledd (radiocarpal, intercarpal, radioulnar), albue, kne, ankel (talocrural) og MTP 1-5, alt bilateralt. En longitudinell UL studie har nylig vist at av sener er det særlig ekstensor carpi ulnaris og den mediale compartment i ankelen som er affisert ved RA.
Crestor® (Rosuvastatin) er vist å ha inflammasjonsdempende egenskaper. UL er en sensitiv metode for å evaluere inflammatorisk aktivitet ved RA. Det er derfor av stor betydning å kartlegge om en ved bruk av Crestor kan se bedring av inflammasjonen etter 3 mnd. I tillegg vil det være av stor interesse å sammenligne funnene fra den omfattende hjerte/kar-undersøkelsen med funn av inflammasjon vurdert ved UL. En slik studie er aldri tidligere utført, og det vil være av betydning for behandlingen av RA pasientene om en skulle finne sterke assosiasjoner mellom hjerte/kar-patologi og grad av UL patologi.

Praktisk gjennomføring:
Etter at hjerte/kar undersøkelsen er gjennomført ved poliklinikken, undersøkes pasienter som har RA med UL av 32 ledd og 4 sener (som beskrevet over). Denne undersøkelsen vil foregå i et eget rom med pasienten sittende i en stol og armen hvilende på et bord for undersøkelsen av hånd/fingre og albue, og liggende på en benk for undersøkelse av kne, ankel og tåledd. Total tid for UL undersøkelsen er ca 15min.

Oslo04.08.10

Endringsprotokoll for prosjektet:

”Kolesterolavleiring i arteria carotis og koronar arterier og effekten av Rosuvastatin ved revmatoid artritt, ankyloserende spondylitt og andre inflammatoriske artritter (RORA-studien)”

Ansvarlig: David Russel, MD, PhD, professor nevrologisk avd, Oslo Universitets sykehus, Rikshospitalet

Tillegg:
- MR av carotisplakk
- Diffusjonsvektet cerebral MR for påvisning av mikroembolier/små iskemiske lesjoner
- Inflammasjon i aterosklerotiske plakk og ledd hos pasienter med reumatoid artritt karakterisert med FDG-PET/CT

I tillegg til undersøkelser allerede beskrevet i protokollen som er godkjent av regional etisk komité (2009/2219-1), søkes det herved om å få godkjenning til å utføre MR undersøkelse av kolesterolavleiringen i arteria carotis, diffusjonsvektet MR av hjernen, og helkropps FDG-PET/CT hos et utvalg av studiens pasienter ved oppstart av studien og etter 18 mnd. behandling med Rosuvastatin.

Formålet med MR av carotisplakk og hjerne samt FDG-PET/CT vil være å evaluere volumvurdering av aterosklerotiske plakk i arteria carotis, i koronar kar og aorta thorakalis/abdominalis, samt plakkenes beskaffenhet som komposisjon vedr stabilitet/ustabilitet før og etter behandling med Rosuvastatin i 18 mnd. Man vil også evaluere sammenhengen mellom arteria carotis plakk og hjerne slag. I tillegg vil man kunne validere inflammasjonen i ledd hos pasienter med revmatoid artritt. FDG-PET/CT vil være gull standarden ved vurdering av inflammasjon ledd sammenliknet med ultralyd undersøkelse.

MR og PET/CT undersøkelsene vil foregå ved Oslo universitetssykehus, Rikshospitalet.

Magnetresonsanstomografi (MR)
I en MR maskin dannes bildeinformasjon ved innsending av radiosignal i et magnetfelt. Dette gir høyoppløselige bilder.

MR av carotisplakk:
Ved en MR undersøkelse av carotisplakket vil vi få en svært detaljert og objektiv framstilling av karveggen og de ulike komponentene i kolesterolavleiringen sammenlignet med det en ultralydundersøkelse gir. MR undersøkelsen vil bli utført på en 3.0 Tesla Achieva MR (Phillips) med en spesialutviklet spole for carotis avbilding. Ved en MR undersøkelse kan vi få detaljert informasjon om plakkets volum, plakkets komponenter som fettrik/nekrotisk kjerne og blødning samt om plakkets

overflate (fibrøse kappe er tykk eller tynn og sprukken). Den kontrastforsterkede delen av MR undersøkelsen er nødvendig for detaljert framstillingen av den fibrøse kappen og måling størrelsen på den lipidrike nekrotiske kjernen. Kontrastoppladning i plakket kan også være et uttrykk for økt vaskularisering som igjen kan bety en økt inflammasjonsgrad. Flere studier har vist at disse ovenfor nevnte plakk-karakteristika gir informasjon om ustabilitet og risikoen for plakkprogresjon. Ved å gjøre MR av carotis ved oppstart og etter 18 mnd kan vi måle reduksjon av plakkvolum og endring av plakk karakteristika under behandling med Rosuvastatin. Programvaren MRI-PlaqueView (VPDiagnostics, Seattle, WA).vil bli benyttet til automatisert måling av endring i plakkvolum.

Diffusjonsvektet cerebral MR:
En diffusjonsvektet MR sekvens (DWI) av hjernen er sensitiv for deteksjon av små iskemiske forandringer. Kolesterolavleiringer i halspulsåren er en av kildene til arterielle embolier i hjernen. Ved å gjøre DWI ved oppstart og etter 18 mnd kan vi måle evt. antall nye iskemiske lesjoner på grunn av mikroembolier under behandling med Rosuvastatin.

DWI undersøkelsene vil bli vurdert etter følgende skala: (i) normal (ingen cerebrale lesjoner), (ii) grensetilfelle (lesjoner med diameter<5 mm), and (iii) patologisk (lesjoner med diameter ≥5 mm). Kriteriet for en signifikant endring fra før til etter behandling er en eller flere nye fokale høyintense lesjoner (diameter ≥2.0mm).

Praktisk gjennomføring av MR undersøkelse:
Pasienten må fylle ut en sjekkliste før MR undersøkelsen. Da det brukes et sterkt magnetfelt kan en ikke ha operert inn metall i kroppen. Selve undersøkelsen tar ca 10 min for avbilding av hjernen noe lenger for avbilding av halspulsåren. Det er ingen kjente bivirkninger ved en MR undersøkelse. Risiko ved MR undersøkelsen er knyttet til bruk av kontrastmiddel og er minimal hos pasienter med normal nyrefunksjon. Kontrastmiddelet som brukes ved MR av halspulsåren er i allmenn klinisk bruk. Alvorlige bivirkninger er sjeldne (<1:1000).

FDG PET/CT
Ved positron-emisjons-tomografi (FDG-PET) undersøkelse injiseres pasienten med radioaktivt merket sukker (glukose) kalt 18F-FDG (FDG). FDG tas opp i områder i kroppen med høy metabolisme. FDG gir opphav til fotoner med høy energi som går ut av kroppen og detekteres i PET-maskinen. Dette gjør det mulig å danne bilder av områder i kroppen med høyt FDG/glukose -opptak. PET med glukoseanalogen FDG benyttes hyppigst for diagnostikk av kreft, men har også vist stor nytte innen diagnostikk av inflammatoriske tilstander. FDG tas opp av celler med høy metabolsk aktivitet, inkludert celler som er aktive i den inflammatoriske prosessen. Ny kunnskap indikerer at ved aterosklerotiske plakk (kolesterolavleiringer) er plakkinflammasjonen den sentrale prosessen som forklarer progresjon og at plakket blir symptomgivende (dvs. gir hjerneslag eller hjerteinfarkt). Multiple studier har vist at FDG opptak i aterosklerotiske plakk korresponderer med makrofagrike områder av plakket. Studier har også vist samsvar mellom symptomer og grad av FDG opptak i plakk i halspulsåren og kransarteriene. RORA-studiens pasienter har økt risiko for både hjerneslag og hjertesykdom ved at de har kolesterol plakk. FDG-PET vil gi viktig tilleggsinformasjon om graden av

inflammasjon i de kolesterolplakkene som er påvist ved strukturell/anatomisk diagnostikk ved ultralyd av halskar og CT av koronarkar. I tillegg vil FDG-PET gi informasjon om grad av aktivitet/inflammasjon i pasientens grunnsykdom ved at leddene vil bli avbildet (flere studier har korrelert økt FDG opptak med andre mål på aktiv leddinnflammasjon). Det er uklart hvorvidt og evt. hvordan inflammasjonsgraden i pasientens grunntilstand/leddsykdom har påvirkning på inflammasjonen/progresjonen i den aterosklerotiske sykdommen. FDG-PET vil gi muligheten til å studere denne samvariasjonen. FDG-PET undersøkelse av leddene vil også bli sammenholdt med UL/Doppler undersøkelse av leddinflammasjonen og klinikk.

Crestor® (Rosuvastatin) er vist å ha inflammasjonsdempende egenskaper. FDG-PET/CT er en sensitiv metode for å evaluere inflammatorisk aktivitet både i aterosklerotiske plakk og i ledd. Små studier har vist redusert FDG opptak i aterosklerotiske plakk i aorta og arteria carotis etter statin behandling. Dette har ikke vært studert i koronarkar, og heller ikke hos pasienter med systemiske kroniske inflammatoriske tilstander. Det er derfor av stor betydning å kartlegge om en ved bruk av Crestor® kan se bedring av inflammasjon i både kar og ledd hos denne pasientgruppen etter 18 mnd. I tillegg vil det være av stor interesse å sammenligne PET undersøkelsen med funnene fra den strukturelle avbildingen av plakkene (UL halskar og CT coronar angio).

Praktisk gjennomføring PET/CT:

FDG PET/CT vil bli utført med Biograph 64 (TruePoint PET·CT, Siemens Medical Solutions). Pasientforberedelse vil bestå i en fett-og proteinrikt diett det siste døgnet før undersøkelsen, faste de siste 6 timer og inntak av et glass vegetabilsk olje 1 time før injeksjon av FDG (5MBq/kg). Dette er for å unngå FDG opptak i kroppens muskulatur generelt (faste), og i hjertemuskulatur (diett og faste) som vil ødelegge for avbildingen av karene. Avilding starter ca. 60-90 min etter injeksjon fra tær til skuldernivå. Deretter vil det bli gjort et eget bildeopptak over arteria carotisbifurkaturen og tilslutt et eget hjerteopptak på ca.10 min med både EKG- og respirasjonsjustering. PET bildene vil bli vurdert ved visuell bedømming og semikvantitativ (SUV-standardized uptake value) måling av FDG opptaket i aorta thorakalis/abdominalis, i plakk i carotis, i veggforandringer i koronarkar dersom det er påvist og i alle aktuelle perifere ledd.

Forespørsel om deltakelse i legemiddelutprøving

"Konsekvenser av kolesterolsenkende behandling på kolesterol avleiring i halspulsårer og hjertets kransårer hos pasienter med artritter"

Bakgrunn og hensikt

Dette er en forespørsel til deg om å delta i et forskningsprosjekt som innebærer utprøving av legemiddelet: Crestor®, (Rosuvastatin). Hensikten er å kartlegge effekten av kolesterolsenkende behandling på selve kolesterol avleiringen i halsens pulsåre, men også undersøke generelt hjerte-kar status hos personer som tidligere er behandlet for Revmatoid artritt, Bekhterev sykdom eller annen form for artritt. Du får denne henvendelsen fordi du tidligere har vært undersøkt/behandlet ved revmatologisk avdeling, Diakonhjemmet Sykehus og hvor vi har påvist kolesterolavleiring i halsens pulsåre hos deg. Crestor® er godkjent til bruk i alle land i Europa og ble godkjent i Norge for bruk i oktober 2008. I Norge avventer man godkjenning for markedsføring av Crestor. Mennesker som har en betennelsesaktig leddsykdom har forhøyet risiko for hjerte-kar sykdom. Kolesterol-avleiring i pulsårer gir også forhøyet risiko for hjerte-kar sykdom, dvs. hjerteinfarkt og hjerneslag. Ved å behandle med kolesterolsenkende medisin, ønsker vi å se på effekten på selve kolesterol avleiringen, samt effekten på hjertets krans årer (pulsårer) og hjertets funksjon. Diakonhjemmet Sykehus AS er ansvarlig for studien.

Hva innebærer studien?

Deltagelse i studien innebærer at en kolesterolsenkende medisin vil bli foreskrevet og du vil bli fulgt opp i 1.5 år vedr kolesterolavleiringen i halsens og hjertet pulsårer, effekten på mindre pulsårer (på armen og i fingrene) og hjertefunksjonen. Det vil bli tatt blodprøver i studien og du vil bli bedt om å fylle ut noen spørreskjemaer. Deltagelse innebærer også at opplysninger fra din journal blir brukt i studien. Eventuelle opplysninger om deg i Oslo RA-register, Euridiss registeret, TAK-studien eller Bekhterev-registeret blir også brukt i studien.
Deltagelse i studien innebærer at du får samme medikamentelle behandlings tilbud, som du ville ha fått dersom du ikke var med i studien. Fordelen ved å være med i studiet er at du vil få grundigere oppfølgning. CT undersøkelse av kransårene benyttes i dag hos pasienter med mistenkt sykdom i kransårene og kan være en aktuell undersøkelse også om du ikke blir med i studien. Dersom du ikke ønsker å være med i studiet, vil dette derfor ikke ha konsekvenser for din videre oppfølging og behandling av dine sykdommer. Du vil ha lik tilgang til revmatologisk spesialist, hjertemedisinsk vurdering og behandling, og eventuelle tilleggsundersøkelser. Se ellers detaljer om hva som skjer i studien i Kapittel A nedenfor.

Mulige fordeler, ulemper og alvorlige bivirkninger

Fordelene ved deltagelse i dette studiet er at du får undersøkt din hjerte-kar -status og -risiko samt behandlet ditt kolesterol. Hvis det er indikasjon for behandling av forsnevring på hjertets kransårer vil du få utført dette.

Vi har valgt å bruke den kolesterolsenkende medisinen; Crestor, av flere grunner. Crestor er vist å kunne skrumpe kolesterol avleiringer i hjertets kransårer i et annet studie. I tillegg er Crestor generelt godt tolerert, men kan ha bivirkninger som muskel plager, tretthet, mavesmerter, kvalme (0,1 %) og hodepine og forstoppelse. Disse er som regel milde og forbigående. I noen tilfeller (0,2 %) kan leverblodprøver bli forøket. Eggehvite i urin kan forekomme, men i de fleste tilfeller er dette forbigående. Alvorlig skjellet muskel påvirkning oppstår sjelden (0,01 %). For å følge opp dette vil det

taes det blodprøver/urin prøve hver 3. måned. Sikkerhetsprofilen for Crestor er lik den for de andre statiner som er på markedet (Lipitor/Simvastatin/Zocor/Pravachol og Leschol.

Hva skjer med prøvene og informasjonen om deg?

Prøvene tatt av deg og informasjonen som registreres om deg skal kun brukes slik som beskrevet i hensikten med studien. Alle opplysningene og prøvene vil bli behandlet uten navn og fødselsnummer eller andre direkte gjenkjennende opplysninger. En kode knytter deg til dine opplysninger og prøver gjennom en navneliste. Listen som kan koble ditt navn til koden vil kun bli oppbevart på legesenteret/sykehuset og bare personell med ansvar for studien har tilgang til denne. Listen vil oppbevares i 15 år etter at sluttrapport fra studien er skrevet. Det forventes at listen vil slettes i 2025, men lengre oppbevaring kan være nødvendig grunnet krav fra legemiddelmyndighetene. Det vil ikke være mulig å identifisere deg i resultatene av studien når disse publiseres.

Frivillig deltakelse

Det er frivillig å delta i studien. Du kan når som helst og uten å oppgi noen grunn trekke deg fra studien uten at det får konsekvenser for din videre behandling.

Dersom du ønsker å delta inviterer vi deg til et møte der vi vil gi muntlig informasjon om studien og der det er anledning til å få svar på det du lurer på om studien. Dersom du etter dette fortsatt ønsker å delta vil vi be deg om å signere en erklæring.

Har du spørsmål til studien, ta kontakt med forskningskoordinator Tone Omreng, tlf 22 45 17 47, som vil formidle kontakt til prosjektansvarlige som er Avdelingssjef/professor Tore K Kvien og forsker/overlege/dr med/ hjertespesialist Anne Grete Semb

Ytterligere informasjon om studien finnes i kapittel A – *utdypende forklaring av hva studien innebærer.*

Ytterligere informasjon om biobank, personvern, økonomi og forsikring finnes i kapittel B – *Personvern, biobank, økonomi og forsikring.*

Samtykkeerklæring følger etter kapittel B. – *Signeres av både dem som samtykker til å delta i studien. Personen, som har informert om studien, kan bekrefte at informasjonen er gitt..*

Kapittel A- utdypende forklaring om hva studien innebærer

Alle med revmatisk leddlidelse og påvist kolesterolplakk i halsens pulsåre som tidligere er undersøkt hos oss vil kunne få delta i denne studien, som er mellom 35 og 80 år gamle, som ikke står på kolesterol senkende medikasjon fra før. De som har ande alvorlige sykdommer eler bruker andre medisiner hvor man ikke samtidig kan bruke Crestor, kan ikke være med i studien. Gravide og ammende kan ikke delta i studien. Vi ber også om opplysning om bruk av sikker prevensjon. Vi vil få klarhet i faktorer rund dette, gjennom en samtale på informasjonsmøtet med deg. De som deltar i studien vil få en full hjerte-kar undersøkelse og vurdering samt bli fulgt opp vedr funn som krever videre undersøkelser og behandling.

Dersom du ikke ønsker å delta i studien vil dette ikke få konsekvenser for deg. Det vil si at du vil ha lik mulighet til undersøkelser, vurdering og oppfølgning av revmatolog og hjertespesialist. Fordelen ved å være med i studiet er at du får en grundigere gjennomgang av din hjerte-kar status enn det som er vanlig ved polikliniske konsultasjoner.

Alle som velger å være med i studien, vil du få den kolesterolsenkende behandlingen med Crestor i 18 måneder, i tillegg til en rekke undersøkelser beskrevet nedenfor. De forskjellige undersøkelsene vil bli utført etter tidsskjema nedenfor:

**Tids forløp**	screening	Baseline: besøk 1	1,5 - 2 - 2,5 uker	3 mnd	6 mnd	12 mnd	18 mnd
Blod prøver	X	X	X	X	X	X	X
Crestor 20 mg X 1, for de eldre enn 70 år 5mg X 1		start	Evt. opptitrering av Crestor dosen	Evt. opptitrering av Crestor dosen			
Undersøkelse av sykdomsaktivitet vedr ledd sykdom		X					X
Utfylling av skjemaer vedr 1) hjertekar 2) livskvalitet		X					X
EKG	X	X		X			X
Blod trykk/pulsåre stivhet		X					X
Ultralyd: Halspulsårene	X	X		X			X
Ultralyd: Hjertet	X	X		X			X
Blod prøver til biomarkører	X	X		X			X
CT røntgen av hjertets kransårer		X					X
Evt. koronar-angiografi med IVUS		X					X
RH: MR halsen+hodet PET scan							
bivirknings registr.			X	X	X	X	X

Hvis du sier ja til å delta i studien betyr det at du vil møte på revmatologisk og hjertemedisinsk poliklinikk ved Diakonhjemmet Sykehus 1 full dag (eller 2 halve dager) og gjennomgå en full klinisk undersøkelse som inneholder:

- svare på spørreskjemaer angående sykdomsaktivitet, fysisk funksjon og vaner for fysisk aktivitet (ca 20 minutter)
- undersøkelse av sykdomsaktivitet relatert til din leddsykdom

Vi vil dessuten ha et spesielt fokus på kartlegging av mulige sykdomskomplikasjoner knyttet til hjerte og blodkar:

- Spørreskjema over forhold som kan være relatert til forekomst av hjerte-karsykdom hos deg eller din familie
- Blodprøver (ca 20 ml).
- EKG undersøkelse hvor man fester elektroder på brystet for å måle hjertets elektriske aktivitet.
- Undersøkelse av fingertuppens små blodårer ved at en hylse settes på tuppen av en finger.
- Trykkmåling og måling av blodstrømmen i håndleddets pulsåre samt i lyske Undersøkelsen utføres ved hjelp av en probe som klemmes lett mot huden.

- Måling av blodstrømmen i armens pulsåre etter 5 minutters avklemming ved hjelp av en blodtrykksmansjett som blir pumpet opp rundt overarmen.
- Tilsvarende måling av blodstrømmen i armens pulsåre etter å ha fått en tablett nitroglyserin. Nitroglyserin gir en forbigående avslapning av blodårene og er svært vanlig brukt i behandlingen av hjertekrampe. Medikamentet har ingen alvorlig bivirkningen men kan gi en forbigående hodepine eller ubehag forårsaket av blodtrykksfallet hos noen.
- Ultralydmåling av halsens hovedpulsåre med spesielt fokus på kolesterolavleiring, ved hjelp av en probe som holdes mot halsen.
- Ultralyd og doppler undersøkelse av hjertet
- CT undersøkelse av hjertet (utføres på Ullevål Universitets sykehus) for å kartlegge kolesterolavleiringer i hjertets pulsårer.

De som får påvist kolesterol avleiring i hjertets kransårer vil også få tilbud om å få utført en

- Venstresidig hjertekateterisering og koronar angiografi (se nedenfor) på Ullevål Universitets sykehus. Undersøkelsen gjøres poliklinisk. Undersøkelsen innbefatter ultralyd av kransarteriene.

Alle resultatene vil avidentifiseres før de analyseres forskningsmessig, men de vil også bli en del av din sykehusjournal. På den måten vil resultatene være en status som kan brukes for å undersøke om din helsetilstand endrer seg senere.

Beskrivelse av undersøkelsene og deres risiko og ubehag

Undersøkelsene ved Diakonhjemmet foregår utenpå huden har ingen risiko for komplikasjoner. CT undersøkelsen er en røntgenundersøkelse der det blir gitt kontrastmiddel. Undersøkelsen er i vanlig bruk og innebærer liten risiko knyttet til bruk av røntgen og til bruken av kontrastmiddel. Koronar angiografi er også en undersøkelse i vanlig bruk hos pasienter med avleiringer i kransarteriene. Et tynt plastrør (hylse) legges inn i en pulsåre ved håndleddet (vanligst) eller i lysken etter at området er lokalbedøvet. Gjennom dette, fører legen et annet tynt rør, et kateter, opp til hjertet. Kateteret brukes til å sprøyte inn en væske, røntgenkontrast. Dette gjør det mulig å se innsiden av hjertets kransårer ved røntgengjennomlysning og røntgenfilming. Risikoen for komplikasjon ved koronar angiografi er liten (<1/100) og skyldes oftest små blødninger ved stikstedet. Alvorlige komplikasjoner som hjerteinfarkt og hjerneslag forekommer svært sjelden (2-3 promille). I forbindelse med undersøkelse av kransårene vil det bli utført en tilleggsundersøkelse med innvendig ultralydsundersøkelse av åren (IVUS).

Dersom undersøkelsene gir funn som krever behandling, vil vi ta dette opp med deg og fortrinnsvis gjennomføre behandlingen på et senere tidspunkt. Ved utbredt kransåresykdom kan det være aktuelt med utblokking med ballong eller en hjerteoperasjon med bypass.

Den kolesterolsenkende medisinen; Crestor, er godkjent til bruk i Norge fra 7. oktober 2008, men avventer markedsførings tillatelse.

Du vil bli opplyst så raskt som mulig dersom ny informasjon blir tilgjengelig som kan påvirke pasientens/forsøkspersonens/deltakerens villighet til å delta i studien.
Du vil bli opplyst så raskt som mulige om beslutninger/situasjoner som gjør at din deltagelse i studien kan bli avsluttet tidligere enn planlagt.

Pasientens/studiedeltagerens ansvar vil være å rapportere bivirkninger og/eller si ifra dersom man ønsker å avslutte deltagelse i studiet

Kapittel B - Personvern, biobank, økonomi og forsikring

Personvern
Opplysninger som registreres om deg er relevante opplysninger fra din pasientjournal ved Diakonhjemmet sykehus. Dersom du tidligere er registrert i følgende registre vil opplysninger også innhentes fra Oslo RA- register, Euridiss registeret, TAK-studien eller Bekhterev-registeret. Databehandlingsansvarlig for studien er Diakonhjemmet sykehus AS.

Representanter fra Statens legemiddelverk og kontrollmyndigheter i inn- og utland kan få utlevert studieopplysninger og gis innsyn i relevante deler av din journal. Formålet er å kontrollere at studieopplysningene stemmer overens med tilsvarende opplysninger i din journal. Alle som får innsyn i informasjon om deg har taushetsplikt.

Forskningsbiobank
Blodprøvene som blir tatt og informasjonen utledet av dette materialet vil bli lagret i en forsknings biobank ved Diakonhjemmet Sykehus. Hvis du sier ja til å delta i studien, gir du også samtykke til at det biologiske materialet og analyseresultater inngår i biobanken. Avdelingssjef/avdelingsoverlege Tore K Kvien ved revmatologisk avdeling er ansvarlig for biobanken, som er planlagt å vare til 31. desember 2025. Etter dette vil materiale og opplysninger bli destruert/slettet etter interne retnings-linjer.

Behandling av materiale og opplysninger hos andre
Hvis du sier ja til å delta i studien, gir du også ditt samtykke til at røntgen bilder og avidentifiserte opplysninger blir behandlet hos samarbeidspartnere i utlandet når det er nødvendig for å gjennomføre studien. Dette kan være virksomheter som befinner seg i land utenfor EU/EØS-området der personvernlovene er svakere enn i Norge.

Innsynsrett og oppbevaring av materiale
Hvis du sier ja til å delta i studien, har du rett til å få innsyn i hvilke opplysninger som er registrert om deg. Du har videre rett til å få korrigert eventuelle feil i de opplysningene vi har registrert. Dersom du trekker deg fra studien, vil det ikke samles inn flere opplysninger eller mer materiale. Opplysninger som allerede er innsamlet fra deg vil ikke bli slettet.

Finansiering
Denne studien er finansiert gjennom forskningsmidler fra Helse Sør-Øst og fra Diakonhjemmet Sykehus. AstraZeneca A/S produserer den kolesterolsenkende medisinen Crestor og vil gi medikamentet kostnadsfritt til studiet. Prosjektansvarlig og andre som arbeider med prosjektet har ingen form for økonomisk vinning knyttet til prosjektet. Deltagere i studien vil ikke bli økonomisk kompensert. Biobanken er finansiert av forskningsmidler fra Helse SørØst.

Forsikring
Du er forsikret i henhold til Lov om produktansvar i Legemiddelforsikringen.

Resultater
Når resultatene foreligger og er publisert vil alle deltagere i studien få tilsendt informasjon.

Informasjonsmøte om studien

"Konsekvenser av kolesterolsenkende behandling på kolesterol avleiring i halspulsårer hos pasienter med artritter"

Denne siden returneres i vedlagte svarkonvolutt

*Jeg,*____*(navn blokkbokstaver)*

ønsker mer informasjon og vil gjerne *delta på et informasjonsmøte om studien.*

*Signatur*____*Dato*________________________________
(sign. prosjektdeltaker) *(datert av prosjektdeltaker)*

Vennligst oppgi det eller de telefonnumre som vi kan bruke for å gjøre avtaler om tidspunkt møte.

Fast telefon………………………………… Mobil……………………………………

Samtykke for deltakelse i studien

Jeg er villig til å delta i studien

(Signert av prosjektdeltaker, dato)

Bekreftelse på at informasjon er gitt deltakeren i studien

Jeg bekrefter å ha gitt informasjon om studien

(Signert, rolle i studien, dato)

Diakonhjemmet Sykehus

Forespørsel om deltakelse i tileggsundersøkelser i forbindelse med forskningsprosjektet:

"Konsekvenser av kolesterolsenkende behandling på kolesterol avleiring i halspulsårer og hjertets kransårer hos pasienter med artritter"

Bakgrunn

Dette er et spørsmål til deg om å delta i tilleggsundersøkelser i forskningsstudien: "Konsekvenser av kolesterolsenkende behandling på kolesterol avleiring i halspulsårer og hjertets kransårer hos pasienter med artritter" (se eget skriv for detaljer om hovedstudien).

I tillegg til de undersøkelsene du er blitt spurt om å delta i tidligere, får du denne henvendelsen fordi vi ønsker å tilføre studien MR undersøkelser av hjernen og halspulsåren, og en helkropps FDG-PET/CT. Dette vil gi et enda bedre mål på kolesterolavleiringer og din eventuelle sykdomsaktivitet samt Crestors® (Rosuvastatin) effekt på disse.

En MR maskin danner bildeinformasjon ved innsending av radiosignal i et magnetfelt. Dette gir høyoppløselige bilder. MR undersøkelsen av halspulsåren vil gi oss svært detaljert framstilling av kolesterolavleiringen i åreveggen og om denne er blitt mindre etter behandling med Crestors®. MR undersøkelsen av hjernen vil kunne vise om kolesterolavleiringer i blodårene har gitt asymptomatisker små blodpropper til hjernen.

Grad av betennelsesreaksjon både i ledd og i kolesterolavleiringer kan undersøkes med FDG-PET/CT. Ved en PET-undersøkelse injiseres et radioaktivt merket sukker (glukose) kalt 18F-FDG i en blodåre i armen din. FDG (sukker) samler seg i celler i kroppen med høy aktivitet, inkludert aktive betennelsesceller. Dette gjør det mulig å danne bilder av hvilke områder i kroppen som har høy betennelsesaktivitet.

Kolesterolavleiringer med betennelse gir høyere risiko for hjerte-karsykdom enn de uten. FDG-PET vil gi oss informasjon om det er sammenheng mellom betennelsesaktiviteten i ledd og kolestereolavleiringer samt om hvilke av betennelsestilstandene Crestor har effekt på. MR undersøkelsene vil vise oss om Crestor kan redusere størrelsen og alvorlighetsgraden av kolesterolavleiringen samt om den kan redusere antallet nye små blodpropper i hjernen.

Hva innebærer studien?

Som deltaker i tilleggsstudien vil du i tillegg til undersøkelsene i hovedstudien bli spurt om å gjennomføre en MR undersøkelse av hjernen og halspulsåren samt en PET/CT undersøkelse av hele kroppen inkludert ekstraopptak av hjertets kransarterier. Disse undersøkelsene vil bli utført ved behandlingsstart og etter 1,5 år. Undersøkelsene vil bli gjennomført på Rikshospitalet.

Mulige fordeler og ulemper og alvorlige bivirkninger
Fordelene ved deltagelse i dette studiet er at du får undersøkt din hjerte-kar status og behandlet ditt kolesterol. Hvis det er indikasjon for behandling av forsnevring på hjertets kransårer vil du få utført dette. Hvis vi oppdager andre tilstander som trenger videre utredning eller behandling vil du få tilbud om dette. Din deltakelse kan være til hjelp for andre pasienter i fremtiden.
Det er ingen kjente bivirkninger ved selve MR undersøkelsen. Kontrastmiddelet er det samme som brukes i rutinediagnostikk på MR. Risiko ved bruk av MR kontrastmiddel er minimal hos pasienter med normal nyrefunksjon. Da det brukes et sterkt magnetfelt kan du ikke ha operert inn metall i kroppen. Selve undersøkelsen tar ca 10 min av hjernen, noe lenger for avbilding av halspulsåren.

Ved en FDG-PET undersøkelse benyttes et radioaktivt sporstoff. Dette innebærer at kroppen utsettes for en liten mengde stråling på linje med om du skulle tatt et CT bilde av magen. Du vil bli spurt om å ha en protein- og fettrik diett siste døgn samt faste de siste 6 timer før undersøkelsen. Etter at du har fått injeksjon av FDG må du vente ca 90 min til avbilding. Selve avbildingen tar 60-90min (inkludert halspulsårer, hjerte og alle ledd).

Hva skjer med prøvene og informasjonen om deg?

Undersøkelsene/prøvene tatt av deg og informasjonen som registreres om deg skal kun brukes slik som beskrevet i hensikten med studien. Alle opplysninger og prøvene vil bli behandlet uten navn og fødselsnummer eller andre direkte gjenkjennende opplysninger. En kode knytter deg til dine opplysninger og prøver gjennom en navneliste. Listen som kan koble ditt navn til koden vil kun bli oppbevart på legesenteret/sykehuset og bare personell med ansvar for studien har tilgang til denne. Listen vil oppbevares i 15 år etter at sluttrapport fra studien er skrevet. Det forventes at listen vil slettes i 2025, men lengre oppbevaring kan være nødvendig grunnet krav fra legemiddelmyndighetene. Det vil ikke være mulig å identifisere deg i resultatene av studien når disse publiseres.

Frivillig deltakelse

Det er frivillig å delta i studien. Du kan når som helst og uten å oppgi noen grunn trekke deg fra studien uten at det får konsekvenser for din videre behandling.

Har du spørsmål til studien, ta kontakt med forskningskoordinator Turid Agerup, tlf 22 45 40 46, som vil formidle kontakt til prosjektansvarlige som er forsker/overlege/dr med/ hjertespesialist Anne Grete Semb.

**Samtykkeerklæring: Jeg er villig til å delta i studien**	Jeg bekrefter å ha gitt informasjon om studien:
---	--
(Signert av prosjektdeltaker, dato)	(Signert, rolle i studien, dato)
