# Supplementary material for: Sustained Improvement of Arterial Stiffness and Blood Pressure after Long-Term Rosuvastatin Treatment in Patients with Inflammatory Joint Diseases: Results from the RORA-AS Study
Source: PLoS One. 2016 Apr 19;11(4):e0153440. doi: 10.1371/journal.pone.0153440 (PMC4836743; doi:10.1371/journal.pone.0153440)
Supplement: S1 Table — RA: Rheumatoid arthritis, AS: Ankylosing spondylitis, PsA: Psoriatic arthritis, Paired samples t-test by diagnose. (DOCX) [file pone.0153440.s004.docx]

|  | **RA patients** | | | **AS patients (n=23)** | | | **PsA patients (n=11)** | | |
| --- | --- | --- | --- | --- | --- | --- | --- | --- | --- |
|  | Baseline  (Mean+SD) | 18 months  (Mean+SD) | Difference during study (Mean+SD) | Baseline  (Mean+SD) | 18 months  (Mean+SD) | Difference during study (Mean+SD) | Baseline  (Mean+SD) | 18 months  (Mean+SD) | Difference during study (Mean+SD) |
| **AIx** | 29.8+7.4 | 28.1+7.9 | 1.8+7.7 (p=0.09) | 22.3+6.2 | 20.8+7.7 | 1.5+6.4 (p=0.28) | 29.1+6.2 | 27.5+5.9 | 1.6+4.0 (p=0.22) |
| **aPWV** | 8.10+1.56 | 7.71+1.36 | 0.39+1.37 (p=0.06) | 8.05+1.37 | 7.90+1.78 | 0.16+1.46 (p=0.66) | 8.26+1.98 | 7.82+1.36 | 0.44+1.20 (p=0.26) |
| **sBP** | 139.4+18.5 | 134.7+15.8 | 4.7+15.7 (p<0.001) | 141.9+13.9 | 136.4+17.2 | 5.47+19.9 (p=0.07) | 144.6+28.3 | 136.3+10.7 | 8.4+22.2 (p=0.32) |
| **dBP** | 81.6+8.5 | 78.5+8.2 | 3.10+8.8 (p=0.001) | 84.1+7.1 | 80.7+9.9 | 3.47+6.29 (p=0.05) | 85.8+11.9 | 84.8+6.2 | 1.0+12.9 (p=0.83) |
