# Supplementary material for: Sustained Improvement of Arterial Stiffness and Blood Pressure after Long-Term Rosuvastatin Treatment in Patients with Inflammatory Joint Diseases: Results from the RORA-AS Study
Source: PLoS One. 2016 Apr 19;11(4):e0153440. doi: 10.1371/journal.pone.0153440 (PMC4836743; doi:10.1371/journal.pone.0153440)
Supplement: S2 Table — Δ: Change from baseline to study end, aPWV: aortic pulse wave velocity, AIx: Augmentation index, sBP: Systolic blood pressure, dBP: Diastolic blood pressure, bMARDs: Biological disease-modifying anti-rheumatic drugs, sDMARDs: synthetic disease-modifying anti-rheumatic drugs, NSAIDs: Nonsteroidal anti-inflammatory drugs, CRP: C-reactive protein, ESR: Erytrocyte sedimentation rate, DAS28: Disease activity score 28 joints, ASDAS: Ankylosing spondylitis disease activity score, LDL-c: Low-density lipoprotein cholesterol, Unadjusted linear regression analyses. (DOCX) [file pone.0153440.s005.docx]

| **Baseline variable** |  | **Arterial stiffness** |  | **Blood pressure** |
| --- | --- | --- | --- | --- |
|  |  | **β (95% CI) p-value** |  | **β (95% CI) p-value** |
| **Age**  (years) | ΔAIx | 0.07 (-0.10, 0.25 p=0.41 | ΔsBP | -0.32 (-0.73, 0.09) p=0.12 |
|  | ΔaPWV | -0.05 (-0.09, -0.01) p=0.01 | ΔdBP | -0.25 (-0.48, -0.03) p=0.03 |
| **Gender**  (male) | ΔAIx | 1.32 (-1.74, 4.39) p=0.39 | ΔsBP | -1.19 (-9.16, 6.77) p=0.77 |
|  | ΔaPWV | -0.18 (-0.81, 0.46) p=0.58 | ΔdBP | 1.02 (-3.49, 5.52) p=0.65 |
| **bDMARDs** | ΔAIx | 0.35 (-2.79, 3.49) p=0.82 | ΔsBP | -2.54 (-10.25, 5.17) p=0.51 |
|  | ΔaPWV | 0.26 (-0.93, 0.42) p=0.45 | ΔdBP | -0.07 (-4.40, 4.53) p=0.98 |
| **sDMARDs** | ΔAIx | -0.004 (-0.01, 0.003) p=0.27 | ΔsBP | <0.001 (-0.14, 0.15) p=0.95 |
|  | ΔaPWV | <0.001 (-0.001, 0.001) p=0.66 | ΔdBP | 0.001 (-0.01, 0.01) p=0.74 |
| **NSAIDs** | ΔAIx | 1.33 (-1.68, 4.35) p=0.88 | ΔsBP | -1.89 (-9.16, 5.38) p=0.61 |
|  | ΔaPWV | 0.003 (-0.62, 0.63) p=0.99 | ΔdBP | 0.85 (-3.22, 4.92) p=0.68 |
| **AIx**  (%) | ΔAIx | -0.34 (-0.52, 0.16) p<0.001 | ΔsBP | -0.004 (-0.49, 0.48) p=0.99 |
|  | ΔaPWV | -0.07 (-0.99, 0.85) p=0.88 | ΔdBP | 0.01 (-0.27, 0.28) p=0.97 |
| **aPWV**  (m/s^2^) | ΔAIx | -0.01(-0.05, 0.04) p=0.81 | ΔsBP | -4.79 (-7.08, -2.50) p<0.001 |
|  | ΔaPWV | -0.45 (-0.62, 0.28) p<0.001 | ΔdBP | -2.71 (-4.00, -1.42) p<0.001 |
| **sBP**  (mmHg) | ΔAIx | 0.002 (-0.08, 0.08) p=0.96 | ΔsBP | -0.50 (-0.66, -0.34) p<0.001 |
|  | ΔaPWV | -0.02 (-0.03, 0.00) p=0.05 | ΔdBP | -0.23 (-0.33, -0.13) p<0.001 |
| **dBP**  (mmHg) | ΔAIx | -0.10 (-0.27, 0.06) p=0.21 | ΔsBP | -0.71 (-1.10, -0.31) p=0.001 |
|  | ΔaPWV | -0.003 (-0.04, 0.03) p=0.86 | ΔdBP | -0.53 (-0.73, -0.32) p<0.001 |
| **CRP**  (mg/L) | ΔAIx | 0.05 (-0.26, 0.37) p=0.73 | ΔsBP | -0.13 (-0.86, 0.61) p=0.73 |
|  | ΔaPWV | 0.01 (-0.05, 0.08) p=0.71 | ΔdBP | -0.11 (-0.52, 0.31) p=0.61 |
| **ESR**  (mm/h) | ΔAIx | 0.06 (-0.09, 0.22) p=0.42 | ΔsBP | 0.13 (-0.26, 0.52) p=0.52 |
|  | ΔaPWV | -0.01 (-0.04, 0.03) p=0.68 | ΔdBP | -0.03 (-0.25, 0.19) p=0.81 |
| **DAS28** | ΔAIx | 0.23 (-0.07, 0.52) p=0.13 | ΔsBP | -0.14 (-0.89, 0.61) p=0.71 |
|  | ΔaPWV | 0.02 (-0.06, 0.09) p=0.67 | ΔdBP | -0.11 (-0.56, 0.33) p=0.62 |
| **ASDAS** | ΔAIx | -0.20 (-1.51, 1.11) p=0.75 | ΔsBP | 0.06 (-2.58, 2.69) p=0.97 |
|  | ΔaPWV | -0.13 (-0.45, 0.19) p=0.40 | ΔdBP | 0.58 (-1.24, 2.39) p=0.51 |
| **LDL-c**  (mmol/L) | ΔAIx | 0.01 (-1.61, 1.63) p=0.99 | ΔsBP | 0.02 (-3.86, 3.89) p=0.99 |
|  | ΔaPWV | 0.13 (-0.20, 0.47) p=0.43 | ΔdBP | -0.05 (-2.24, 2.15) p=0.97 |
| **Uric acid**  (µmol/L) | ΔAIx | -0.01 (0.03, 0.01) p=0.40 | ΔsBP | 0.04 (-0.09, 0.002) p=0.06 |
|  | ΔaPWV | -0.003 (0.01, 0.001) p=0.19 | ΔdBP | -0.01 (-0.04, 0.12) p=0.29 |
