# Supplementary material for: Sustained Improvement of Arterial Stiffness and Blood Pressure after Long-Term Rosuvastatin Treatment in Patients with Inflammatory Joint Diseases: Results from the RORA-AS Study
Source: PLoS One. 2016 Apr 19;11(4):e0153440. doi: 10.1371/journal.pone.0153440 (PMC4836743; doi:10.1371/journal.pone.0153440)
Supplement: S3 Table — Δ: Change from baseline to study end, AIx: Augmentation index, aPWV: aortic pulse wave velocity, sBP: systolic blood pressure, dBP: diastolic BP, BMI: Body mass index, Anti-HT: Antihypertensive medication (beta receptor antagonist, calcium channel antagonists, angiotensin converting enzyme inhibitors, angiotensin II receptor antagonists, diuretics), AntiHT change: New antihypertensive medication, drug switch or dose adjustment, Physical activity: ≥ 1 per week, reported on patient questionnaire, CRP: C-reactive protein, AUC: Area under the curve, ESR: Erytrocyte sedimentation rate, DAS28: Disease activity score 28 joints, ASDAS: Ankylosing spondylitis disease activity score, LDL-c: Low-density lipoprotein cholesterol, c-IMT: Carotid intima-media thickness, CP: Carotid plaque. Unadjusted linear regression analyses. (DOCX) [file pone.0153440.s006.docx]

|  | | **Arterial stiffness**  **β (95% CI) p-value** |  | **Blood pressure**  **β (95% CI) p-value** |
| --- | --- | --- | --- | --- |
| **Anti-HT change** | ΔAIx | -0.17 (-4.51, 4.17) p=0.94 | ΔsBP |  |
|  | ΔaPWV | -0.16 (-1.08, 0.76) p=0.74 | ΔdBP |  |
| **ΔBMI** | ΔAIx | -0.54 (-1.77, 0.68) p=0.38 | ΔsBP | 2.09 (-0.83, 5.00) p=0.16 |
|  | ΔaPWV | 0.13 (-0.16, 0.42) p=0.37 | ΔdBP | 1.10 (-0.55, 2.75) p=0.19 |
| **ΔPhysical activity** | ΔAIx | -0.44 (-2.48, 1.59) p=0.67 | ΔsBP | -1.28 (-6.26, 3.70) p=0.61 |
|  | ΔaPWV | -0.06 (-0.49, 0.37) p=0.78 | ΔdBP | -0.67 (-3.45, 2.12) p=0.63 |
| **∆AIx**  (%) | ΔAIx |  | ΔsBP | -0.04 (0.56, 0.48) p=0.89 |
|  | ΔaPWV |  | ΔdBP | -0.05 (-0.35, 0.24) p=0.71 |
| **∆PWV**  (m/s^2^) | ΔAIx |  | ΔsBP | 6.69 (4.18, 9.21) p<0.001 |
|  | ΔaPWV |  | ΔdBP | 2.80 (1.26, 4.33) p=0.001 |
| **∆sBP**  (mmHg) | ΔAIx | 0.03 (-0.06, 0.11) p=0.56 | ΔsBP |  |
|  | ΔaPWV | 0.02 (0.01, 0.04) p=0.01 | ΔdBP |  |
| **∆dBP**  (mmHg) | ΔAIx | -0.03 (-0.19, 0.13) p=0.71 | ΔsBP |  |
|  | ΔaPWV | 0.05 (0.02, 0.08) p=0.001 | ΔdBP |  |
| **∆CRP**  (mg/L) | ΔAIx | 0.003 (-0.12, 0.13) p=0.96 | ΔsBP | -0.06 (-0.50, 0.38) p=0.80 |
|  | ΔaPWV | -0.02 (-0.04, 0.01) p=0.16 | ΔdBP | -0.05 (-0.29, 0.20) p=0.71 |
| **AUC CRP** | ΔAIx | 0.04 (-0.17, 0.25) p=0.72 | ΔsBP | -0.21 (-0.71, 0.29) p=0.41 |
|  | ΔaPWV | -0.03 (-0.08, 0.01) p=0.10 | ΔdBP | -0.02 (-0.30, 0.26) p=0.90 |
| **∆ESR**  (mm/h) | ΔAIx | -0.02 (-0.15, 0.10) p=0.70 | ΔsBP | -0.12 (-0.46, 0.21) p=0.47 |
|  | ΔaPWV | -0.01 (-0.04, 0.01) p=0.35 | ΔdBP | 0.01 (-0.18, 0.21) p=0.88 |
| **AUC ESR** | ΔAIx | 0.04 (-0.10, 0.17) p=0.60 | ΔsBP | 0.01 (-0.31, 0.34) p=0.95 |
|  | ΔaPWV | -0.02 (-0.04, 0.01) p=0.26 | ΔdBP | 0.05 (-0.13, 0.23) p=0.60 |
| **∆DAS28** | ΔAIx | -0.28 (-0.73, 0.17) p=0.21 | ΔsBP | 0.13 (-1.10, 1.36) p=0.83 |
|  | ΔaPWV | -0.06 (-0.18, 0.05) p=0.28 | ΔdBP | -0.03 (-0.74, 0.69) p=0.95 |
| **∆ASDAS** | ΔAIx | 0.96 (-0.52, 2.43) p=0.18 | ΔsBP | 0.58 (-2.67, 3.83) p=0.70 |
|  | ΔaPWV | -0.20 (-0.65, 0.25) p=0.34 | ΔdBP | 0.04 (-2.29, 2.36) p=0.97 |
| **∆LDL-c**  (mmol/L) | ΔAIx | -0.53 (-2.37, 1.31) p=0.57 | ΔsBP | -0.56 (-4.92, 3.80) p=0.80 |
|  | ΔaPWV | -0.20 (-0.60, 0.19) p=0.31 | ΔdBP | 0.22 (-2.25, 2.69) p=0.86 |
| **Δc-IMT** (mm) | ΔAIx | 4.52 (-13.26, 22.30) p=0.62 | ΔsBP | -29.32 (-73.48,14.84) p=0.21 |
|  | ΔaPWV | 0.98 (-2.75, 4.72) p=0.60 | ΔdBP | -14.38 (-39.44,10.69) p=0.26 |
| **Δ CP height**  (mm) | ΔAIx | 3.10 (-1.48, 7.68) p=0.18 | ΔsBP | 3.63 (-6.83, 14.09) p=0.49 |
|  | ΔaPWV | 0.06 (-0.91, 1.02) p=0.91 | ΔdBP | 1.94 (-4.05, 7.93) p=0.52 |
| **Rosuvastatin dose** (mg) | ΔAIx | -0.06 (-0.16, 0.04) p=0.25 | ΔsBP | -0.05 (-0.34, 0.24) p=0.74 |
|  | ΔaPWV | 0.003 (-0.02, 0.02) p=0.78 | ΔdBP | 0.003 (-0.16, 0.17) p=0.97 |
| **∆Uric acid**  (µmol/L) | ΔAIx | 0.01 (-0.02, 0.04) p=0.63 | ΔsBP | -0.01 (-0.06, 0.09) p=0.76 |
|  | ΔaPWV | 0.03 (-0.003, 0.01) p=0.35 | ΔdBP | 0.003 (-0.04, 0.05) p=0.88 |
